# Evidence that phytochrome functions as a protein kinase in plant light signalling

**DOI:** 10.1038/ncomms11545

**Published:** 2016-05-13

**Authors:** Ah-Young Shin, Yun-Jeong Han, Ayoung Baek, Taeho Ahn, Soo Young Kim, Thai Son Nguyen, Minky Son, Keun Woo Lee, Yu Shen, Pill-Soon Song, Jeong-Il Kim

**Affiliations:** 1 Department of Biotechnology and Kumho Life Science Laboratory, Chonnam National University, Gwangju 500-757, Korea; 2 Division of Applied Life Science (BK21 Plus Program), Systems and Synthetic Agrobiotech Center (SSAC), Plant Molecular Biology and Biotechnology Research Center (PMBBRC), Research Institute of Natural Science (RINS), Gyeongsang National University, Jinju 660-701, Korea; 3 College of Veterinary Medicine, Chonnam National University, Gwangju 500-757, Korea; 4 BioVision Inc., Milpitas, California 95035, USA; 5 Faculty of Biotechnology and Subtropical Horticulture Research Institute, Jeju National University, Jeju 690-756, Korea

## Abstract

It has been suggested that plant phytochromes are autophosphorylating serine/threonine kinases. However, the biochemical properties and functional roles of putative phytochrome kinase activity in plant light signalling are largely unknown. Here, we describe the biochemical and functional characterization of *Avena sativa* phytochrome A (AsphyA) as a potential protein kinase. We provide evidence that phytochrome-interacting factors (PIFs) are phosphorylated by phytochromes *in vitro*. Domain mapping of AsphyA shows that the photosensory core region consisting of PAS-GAF-PHY domains in the N-terminal is required for the observed kinase activity. Moreover, we demonstrate that transgenic plants expressing mutant versions of AsphyA, which display reduced activity in *in vitro* kinase assays, show hyposensitive responses to far-red light. Further analysis reveals that far-red light-induced phosphorylation and degradation of PIF3 are significantly reduced in these transgenic plants. Collectively, these results suggest a positive relationship between phytochrome kinase activity and photoresponses in plants.

## Abstract (web summary)

Phytochromes regulate plant responses to environmental light conditions but despite extensive research the initial events in phytochrome signaling remain uncertain. Here, Shin *et al*. provide evidence that phytochrome phosphorylates target proteins via kinase activity in the N-terminal core domain.

## Graphical abstract

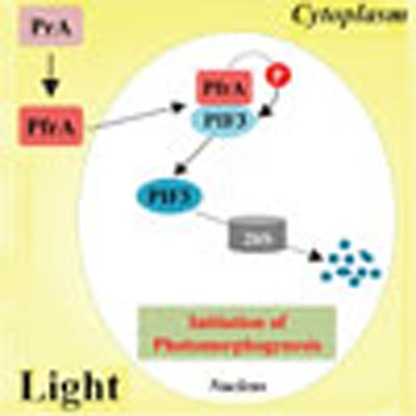

Phytochromes are red- and far-red-absorbing photoreceptors that regulate plant growth and development in response to environmental light conditions^1^,^2^. They exist as dimers with each monomer possessing a covalently linked chromophore, and there are two spectrally distinct red-absorbing Pr and far-red-absorbing Pfr forms. Phytochromes are biosynthesized as the Pr form in the dark, which can be phototransformed into the Pfr form on exposure to red light. The phototransformation between the two forms induces a highly regulated signalling network for photomorphogenesis, which includes translocation of phytochromes into the nucleus, interaction of phytochromes with a wide array of signalling partners, regulated proteolysis of phytochromes and signalling targets, and transcriptional regulation of various photoresponsive genes^3^,^4^,^5^,^6^,^7^. Despite these advances in establishing the phytochrome-mediated light signalling networks in plants, the initial biochemical mechanisms of phytochrome function have not been fully elucidated.

A few decades ago, it was proposed that phytochromes might act as light-regulated protein kinases^8^. This hypothesis was supported by the observation that purified oat phytochrome A (phyA) catalysed the phosphorylation of serine residues on the photoreceptor itself, that is autophosphorylation^9^. Subsequently, it was suggested that eukaryotic phytochromes are histidine kinase paralogs with serine/threonine specificity^10^. Moreover, several proteins were reported to be phosphorylated by phytochromes *in vitro*, such as histone H1, PKS1 (phytochrome kinase substrate 1), cryptochromes, Aux/IAA proteins and FHY1 (far-red elongated hypocotyl 1) (refs 8, 11, 12, 13, 14). While these works suggested that oat phyA is an autophosphorylating serine/threonine kinase, the site of its catalytic activity and the *in vivo* functional role of this kinase activity remain unknown.

The similarity between phytochromes and prokaryotic protein kinases has long been known^15^. Subsequently, a cyanobacterial phytochrome (Cph1) was shown to be a light-regulated histidine kinase^16^, implying that the histidine kinase-related domain (HKRD) in the C-terminal half of plant phytochromes could be responsible for its kinase activity^10^. However, it has been suggested that the HKRD of plant phytochromes is non-functional, because the critical conserved histidine residue for histidine kinase activity is absent and mutating several critical residues required for ATP-binding activity did not qualitatively affect its signalling activity^17^,^18^. It is therefore conceivable that the region responsible for the catalytic activity of plant phytochromes resides in domains other than the HKRD.

Recently, phytochromes have been reported to induce rapid *in vivo* phosphorylation and degradation of PIFs (phytochrome-interacting factors)^19^,^20^,^21^. PIFs are a small subset of basic helix–loop–helix transcription factors that are known to be central players in phytochrome-mediated signalling networks^7^,^22^. The physical interaction of phytochromes with PIFs is known to lead to the latter's phosphorylation, and subsequent degradation via the 26S proteasome. Altogether, these processes permit rapid regulation of gene expression in response to fluctuations in environmental light. Therefore, the phytochrome-induced phosphorylation of downstream signalling components, such as PIFs, may play a direct role to target these and other proteins for proteasome-mediated degradation.

In this study, we first provide evidence that PIFs are phosphorylated by phytochromes *in vitro*. Then, to investigate the *in vivo* function of phytochrome kinase activity, we determine the likely kinase domain of *Avena sativa* phyA (AsphyA) using PIF3 as a substrate and obtain AsphyA mutants displaying reduced kinase activity. Subsequently, we demonstrate that transgenic *phyA-201* plants expressing AsphyA mutants with reduced kinase activity show reduced photoresponses to far-red light, accompanied by reduced phosphorylation and degradation of PIF3 as compared with transgenic plants expressing wild-type (WT) AsphyA. Therefore, our results support the hypothesis that phytochromes act as protein kinases in plant light signalling and suggest that phytochrome-mediated phosphorylation participates in the initial steps of phytochrome signalling.

## Results

### Phosphorylation of PIFs by phytochromes *in vitro*

**Figure 1:**
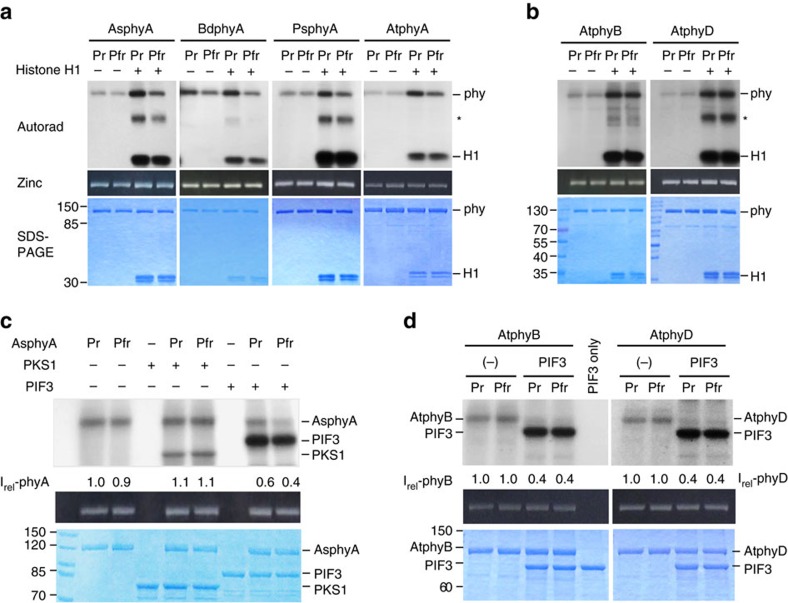
Evidence that PIF3 is phosphorylated by phytochromes. (**a**,**b**) Autophosphorylation and protein kinase activities of type I (**a**) and type II (**b**) phytochromes. Histone H1 (H1) was included as a substrate for phytochrome kinase activity, and also used to stimulate phytochrome autophosphorylation. Autoradiograms (top), zinc-fluorescence assays (middle), and SDS-PAGE gels (bottom) are shown. The asterisk indicates a protein band that originated from histone H1 (see Supplementary Fig. 1). (**c**,**d**) Phosphorylation of PIF3 by AsphyA (**c**), and by AtphyB and AtphyD (**d**). In all, 1.0 μg of GST/strep-fused PIF3 (∼0.3 μM) was included as a substrate and the reactions were performed in the absence of histone H1. PKS1 (phytochrome kinase substrate 1) was included as a control. Intensities (I_rel_) of AsphyA, AtphyB and AtphyD autophosphorylation are expressed relative to the first lanes (that is, Pr forms in the absence of a substrate). AsphyA, *Avena sativa* (oat) phyA; AtphyA, AtphyB and AtphyD, *Arabidopsis thaliana* phyA, phyB and phyD; BdphyA, *Brachypodium distachyon* phyA; *Pisum sativum* phyA; Pr/Pfr, red/far-red light-absorbing forms of phytochromes.

Phytochromes are suggested to be autophosphorylating protein kinases, but the protein kinase activity of plant phytochromes has been demonstrated mainly with oat phyA^2^. To examine whether other phytochromes are also protein kinases, we investigated autophosphorylation and kinase activities of various plant phytochromes (Supplementary Note 1). The results showed that all phytochromes tested displayed autophosphorylation activity that was stimulated in the presence of histone H1 (Fig. 1a,b), a result similar to that previously found with oat phyA^10^,^23^. As a control experiment, we confirmed that no phosphorylation was detected in the histone H1 sample only (Supplementary Fig. 1). Furthermore, we observed histone H1 phosphorylation by all the phytochromes tested here. Therefore, these results provide evidence that a broad range of plant phytochromes possess intrinsic autophosphorylation and kinase activities.

It has been reported that interaction with phytochromes triggers *in vivo* phosphorylation of PIFs before proteasome-mediated degradation^19^,^20^,^21^, suggesting that PIFs might be directly phosphorylated by phytochromes. To examine this possibility, we first performed kinase activity assays using purified recombinant AsphyA and GST/strep-fused PIF3 proteins (Supplementary Fig. 2a,b). In this assay, the first reported phytochrome kinase substrate, PKS1 (ref. 12) was also included as a control. The results showed that both PKS1 and PIF3 were phosphorylated in the presence of AsphyA, while no phosphorylation was detected in samples that contained PIF3 or PKS1 alone (Fig. 1c). Under our experimental conditions, the extent of PKS1 and PIF3 phosphorylation was similar for the Pr and Pfr forms. However, the phosphorylation of PIF3 was greater than that of PKS1. More importantly, the AsphyA autophosphorylation reduced in the presence of PIF3, but not in the presence of PKS1. Additional experiments also revealed that both histone H1 phosphorylation and histone H1-stimulated autophosphorylation were significantly reduced in the presence of PIF3 (Supplementary Fig. 3). These results suggest that PIF3 is favored by AsphyA over other substrates, such as AsphyA itself or histone H1. Since PIF3 is also known to interact with phyB, we investigated whether PIF3 could be phosphorylated by AtphyB. Our results suggested that AtphyB could mediate PIF3 phosphorylation (Fig. 1d). Moreover, PIF3 phosphorylation was also mediated by AtphyD, and the autophosphorylation of AtphyB and AtphyD was reduced in the presence of PIF3 (Fig. 1d). Altogether, these results support direct phosphorylation of PIF3 by phytochromes *in vitro*.

It is well-known that phytochromes interact with PIF3 in a Pfr-preferential manner. Our results were, therefore, unexpected in that the extent of PIF3 phosphorylation was similar between Pr and Pfr forms. We would expect that the weaker interaction of the Pr form with PIF3 would require more time to reach a similar phosphorylation level to that of the Pfr form. However, the time-dependent phosphorylation of PIF3 showed no significant difference between Pr and Pfr forms (Supplementary Fig. 3a). To examine the other possibility that the PIF3 interaction with AsphyA differs in the buffer conditions, protein–protein interaction analysis were performed in the kinase assay buffer. In contrast with the Pfr-specific interaction seen in pull-down assay buffer, we found that both Pr and Pfr forms similarly interacted with PIF3 in the kinase assay buffer (Supplementary Fig. 4a). Further studies showed that addition of MgCl_2_ and to a lesser extent ATP, to the pull-down buffer supported interaction of the Pr form with PIF3 (Supplementary Fig. 4b) although it is not clear at this stage how these buffer components influence the interaction.

**Figure 2:**
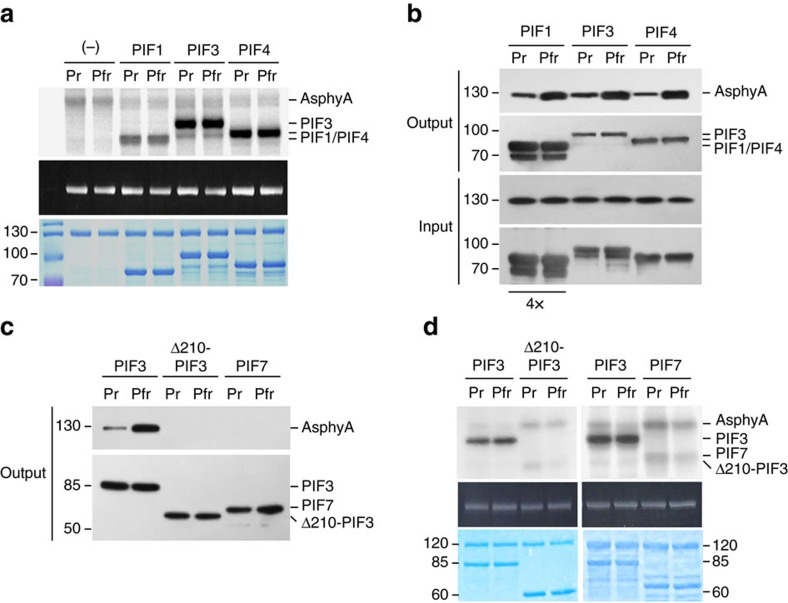
Analysis of AsphyA-PIF interactions and kinase activity. (**a**) Protein kinase activities of AsphyA on other PIFs. In all, 1 μg of full-length AsphyA (∼0.2 μM) and 1 μg of each phyA-interacting PIF (PIF1, PIF3 and PIF4 as substrates) were used for this analysis. AsphyA autophosphorylation without substrate was included as a control. (**b**) Interaction of AsphyA with PIF1, PIF3 and PIF4. The concentration of PIF1 proteins used in this assay was four times higher than that of PIF3 and PIF4. Both Pr and Pfr forms of full-length AsphyA proteins were incubated with PIFs, and glutathione bead-bound proteins were then analysed by western blot analysis with anti-AsphyA (oat22) or anti-GST antibody. (**c**) Protein–protein interaction analysis between AsphyA and APA/APB motif-deleted PIF3 (Δ210-PIF3) or PIF7. (**d**) Phosphorylation of Δ210-PIF3 and PIF7 by AsphyA. PIF3 was included as controls. APA, active phyA-binding motif; APB, active phyB-binding motif.

In addition to PIF3, PIF1 and PIF4 can also interact physically with phyA^24^,^25^,^26^. Therefore, we purified GST/strep-fused *Arabidopsis* PIF1 (NM_179665) and PIF4 (NM_180050), and used them as kinase substrates as well. These studies suggest that AsphyA can support phosphorylation of both substrates (Fig. 2a). As controls, no phosphorylation was confirmed in samples that contained the PIF alone (Supplementary Fig. 5). Our results also suggest that the extent of PIF1 phosphorylation by AsphyA is less than that of PIF3 and PIF4. To explain the different degrees of phosphorylation among the PIFs, we evaluated the protein–protein interaction between AsphyA and PIFs *in vitro* and found that the interaction of AsphyA with PIF1 is weaker than that with PIF3 and PIF4 (Fig. 2b). To obtain a similar level of AsphyA pull-down, approximately four times more PIF1 than PIF3 or PIF4 was needed under our experimental conditions. These results suggest that the physical interaction of PIFs with AsphyA is important for their phosphorylation. To further confirm the importance of physical interaction for phosphorylation, we generated a PIF3 mutant that lacked active phytochrome-binding motifs (that is, Δ210-PIF3). In addition, we also tested PIF7, a protein known not to interact with phyA^27^. Indeed, we found that both Δ210-PIF3 and PIF7 proteins interacted poorly with AsphyA (Fig. 2c) and were found to be poor AsphyA substrates (Fig. 2d). Moreover, we generated an APA mutant of PIF3 (FF/AA; F203 and F209 to alanines)^19^ and confirmed that the APA mutant showed significantly reduced interaction with AsphyA and barely detectable phosphorylation by AsphyA (Supplementary Fig. 6), further indicating that physical interaction of PIFs with AsphyA is correlated with their phosphorylation.

### Kinase activity of the photosensory core of AsphyA

**Figure 3:**
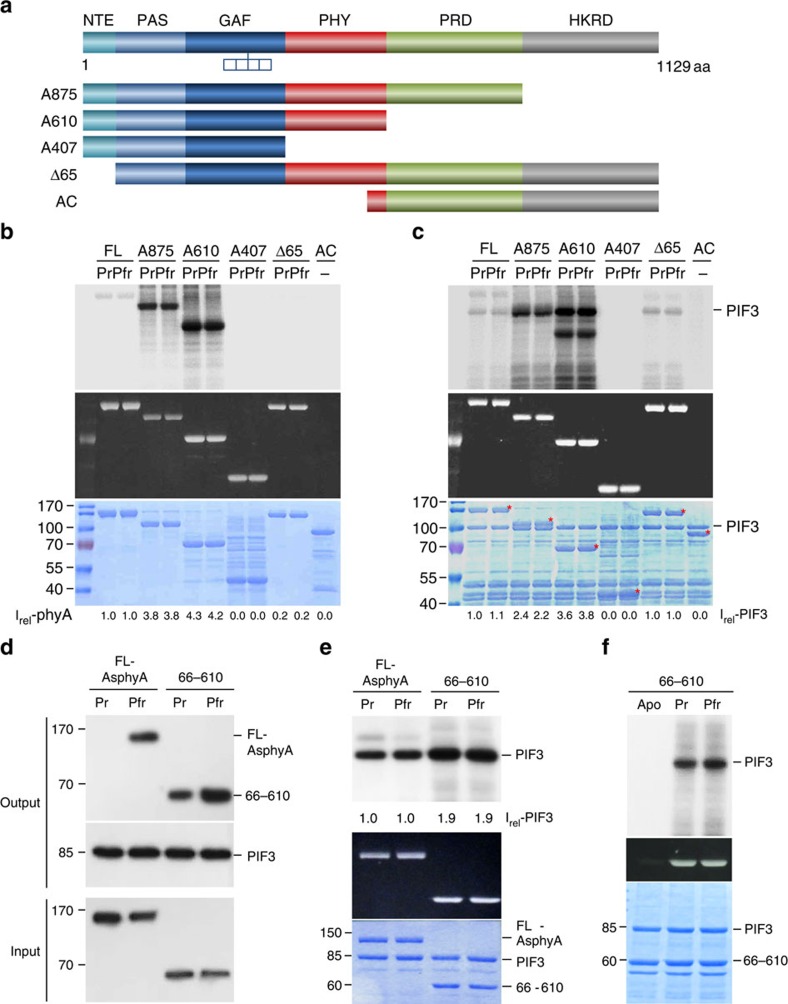
The proposed protein kinase domain of AsphyA resides in the N-terminal photosensory core composed of PAS-GAF-PHY tri-domain. (**a**) Constructs used for kinase domain mapping of AsphyA. (**b**,**c**) Autophosphorylation (**b**) and kinase activity (**c**) assays of domain-deletion AsphyA mutants. PIF3 was used as the substrate for the kinase assays. Asterisks on SDS-PAGE in **c** indicate the corresponding AsphyA protein bands. Intensities (I_rel_) of AsphyA autophosphorylation and PIF3 phosphorylation are normalized on the basis of same molar concentrations and expressed relative to the first lanes (that is, Pr forms of FL-AsphyA). (**d**) Protein–protein interaction analysis between PIF3 and the photosensory core. 1.0 μg of full-length AsphyA (FL-AsphyA) or the photosensory core (66–610aa fragment of AsphyA) was incubated with 1.0 μg of GST/strep-fused PIF3. (**e**) Kinase activity assays of the photosensory core using PIF3 as a substrate. 4 pmol of full-length AsphyA or the photosensory core (66–610) was reacted with 4 pmol of PIF3 for these analyses (that is, ∼0.2 μM for each protein). Intensities of PIF3 phosphorylation are expressed relative to lane 1 (Pr form of FL-AsphyA). (**f**) Kinase activity assays of apo- and holo-proteins of the photosensory core. The apo-proteins (Apo) were prepared from *P. pastoris* cells without addition of chromophore (phycocyanobilin), and the holo-proteins (Pr and Pfr) were prepared by adding phycocyanobilin to purified apo-proteins. Zinc-fluorescence assay (Zinc) was shown to confirm the chromophore-assembled holo-proteins. GAF, cGMP phosphodiesterase/Adenylate cyclase/FhlA domain; NTE, N-terminal extension; PAS, Per/Arnt/Sim domain; PHY, phytochrome-specific GAF-related domain; PRD, PAS-related domain.

The phytochrome molecule is known to be composed of two major functional regions (Supplementary Note 2), including the photosensory core composed of PAS-GAF-PHY architecture that is required for photosensory activity^28^,^29^. To determine the structural basis of kinase activity, we constructed domain-deletion mutants of AsphyA, including A875 (HKRD-deleted), A610 (PRD/HKRD-deleted), A407 (PHY/PRD/HKRD-deleted), Δ65 (NTE-deleted) and AC (C-terminal region only; Fig. 3a). With purified domain-deletion mutant proteins, we performed AsphyA autophosphorylation and PIF3 kinase assays. Surprisingly, both C-terminal deletion mutants A875 and A610 exhibited strong autophosphorylation and PIF3 kinase activities (Fig. 3b,c). Compared with the full-length AsphyA, A875 and A610 showed approximately fourfold more autophosphorylation and about two to fourfold more PIF3 phosphorylation, respectively (I_rel_ in Fig. 3b,c). Under the same conditions, A407 and AC exhibited little autophosphorylation and kinase activities. These results indicate that the N-terminal region, not the C-terminal region, is important for the phytochrome kinase activity we observe. To address this, we also prepared the N-terminal region of AtphyB (1–651aa; B651) and confirmed its kinase activity by autophosphorylation analysis (Supplementary Fig. 7). By comparison, the NTE-deleted Δ65 mutant supported PIF3 phosphorylation similar to full-length AsphyA, but did not show autophosphorylation (Fig. 3b,c). The latter can be explained by the fact that autophosphorylation sites located in the NTE are missing in the Δ65 mutant^23^. In addition, Δ65 also mediated the phosphorylation of PIF1 and PIF4 (Supplementary Fig. 5). Overall, these results suggest that the photosensory core of AsphyA is required for both autophosphorylation and PIF3 kinase activities, whereas the C-terminal domains and the NTE are dispensable.

Since our results demonstrated that the photosensory core is responsible for the phytochrome kinase activity we observe, we prepared truncated AsphyA proteins of the photosensory core (that is, 66–610aa; Supplementary Fig. 2c) and used it for PIF3 kinase activity assays. Our results provide evidence that the photosensory core interacted with PIF3 in a Pfr-preferential manner (Fig. 3d), and was sufficient to exhibit kinase activity on PIF3 (Fig. 3e). The photosensory core also exhibited no autophosphorylation activity, which can also be explained by the absence of the NTE. When we compared PIF3 phosphorylation from reactions with the same molar concentrations, the photosensory core phosphorylated PIF3 approximately two times more than full-length AsphyA. To test the possibility that the PIF3 kinase activity observed with the photosensory core is due to a co-purifying kinase(s) from *P. pastoris*, we purified recombinant A610 proteins from *E. coli* and again observed PIF3 phosphorylation activity (Supplementary Fig. 8). Furthermore, we also examined the effect of chromophore binding on the kinase activity using *apo*- and *holo*-proteins of the photosensory core. The results showed that only chromophore-assembled *holo*-proteins phosphorylated PIF3, but not *apo*-proteins of the photosensory core (Fig. 3f). To test whether protein kinase contamination originated from the phycocyanobilin sample, we generated a chromophore-binding-site mutant (C322S) of AsphyA and found that the photosensory core of C322S-AsphyA failed to phosphorylate PIF3 even with the addition of phycocyanobilin (Supplementary Fig. 9). Taken altogether, our data suggest that the chromophore-assembled PAS-GAF-PHY photosensory core is necessary and sufficient for the observed kinase activity on PIF3.

We further investigated the ATP-binding in the photosensory core by performing photoaffinity labelling experiments using 8-N_3_-ATP-biotin-long chain-hydrazone (8-azido-ATP). On the basis of control experiments of the photoaffinity labelling reactions (Supplementary Fig. 10), 10 μM 8-azido-ATP and ultraviolet irradiation of 90 s were applied for the reactions with AsphyA proteins. The results showed that the photosensory core was labelled with 8-azido-ATP and both Pr and Pfr forms were labelled similarly (Supplementary Fig. 11). Moreover, we revealed that 8-azido-ATP-labelled AsphyA exhibited little autophosphorylation and significantly reduced kinase activity (Supplementary Fig. 11d), suggesting that the photosensory core of AsphyA possesses an ATP-binding site. Finally, we tested the number of binding sites (*n*) and dissociation constant (*K*_d_) of ATP in AsphyA proteins by Scatchard plots using BODIPY-FL-ATP. The *n* values of both full-length and the photosensory core of AsphyA were calculated as approximately one per polypeptide, suggesting one ATP-binding site per AsphyA monomer (Supplementary Fig. 12). The *K*_d_ values were calculated as ∼0.87 × 10^−6^ M for full-length AsphyA and 0.72 × 10^−6^ M for the photosensory core.

### Generation of AsphyA mutants with impaired kinase activity

**Figure 4:**
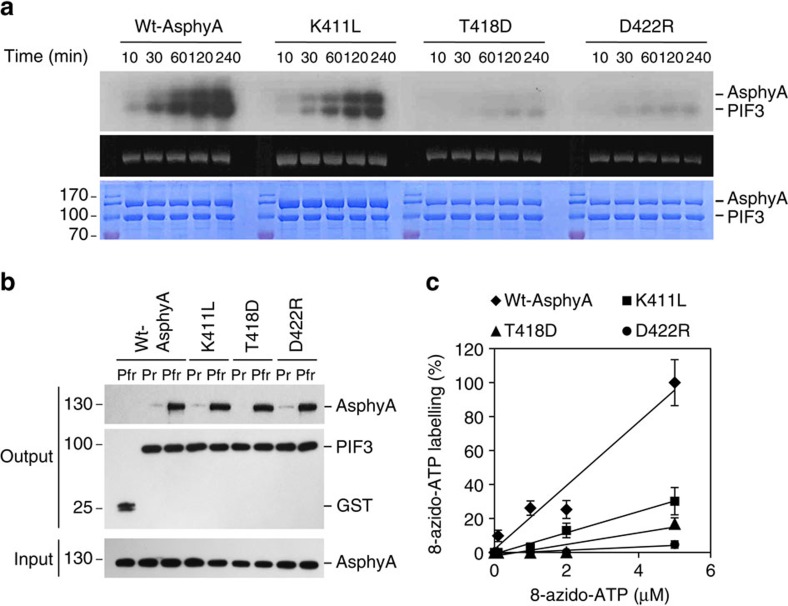
Phosphorylation analyses of AsphyA mutants with reduced kinase activity. (**a**) Kinase activity assays of the AsphyA mutants. In all, 1.0 μg of GST/strep-fused PIF3 (∼0.3 μM) was added as a substrate in reaction mixtures with 1.0 μg of either full-length WT or a mutant (K411L, T418D or D422R) AsphyA protein (∼0.2 μM). (**b**) Protein–protein interaction analysis between PIF3 and the AsphyA kinase mutants. GST was included as a negative control. (**c**) ATP-binding affinity assays of AsphyA kinase mutants using photoaffinity labelling with 8-azido-ATP. 1.0 μg of full-length AsphyA protein (∼80 nM) was labelled with the indicated concentrations of 8-azido-ATP. The percentages of 8-azido-ATP labeling were obtained where the labelling of WT AsphyA with 5 μM azido-ATP was assumed as 100%. Error bars represent s.d. from three independent measurements.

To test the potential functional roles of this protein kinase activity *in vivo*, we sought AsphyA mutants with reduced kinase activity. On the basis of the results of proteomics analysis (Supplementary Note 3) and a previous report^30^, several highly conserved amino acids, including E410, K411, L414, T418, D422 and L424, were chosen for mutagenesis to reduce or impair the ATP binding (Supplementary Fig. 13). After preparing purified full-length AsphyA mutants, kinase activity assays led to identification of three mutants with reduced kinase activity, while other mutants such as E410Q, L414Q and L424E showed similar kinase activities to WT AsphyA (Supplementary Fig. 14). The K411L mutant showed significantly reduced kinase activity, while T418D and D422R mutants showed little kinase activities (Fig. 4a, Supplementary Fig. 15a).

To account for the reduced kinase activity of the AsphyA mutants, we first examined the absorption and difference spectra, and demonstrated that the photochemical properties of all three mutants were very similar to those of WT AsphyA (Supplementary Fig. 16a). We also confirmed no difference in dark reversions between the WT and the kinase mutants (Supplementary Fig. 16b). We further investigated the dimerization of AsphyA proteins by size-exclusion chromatography, and the molecular masses of WT, K411L, T418D and D422R AsphyA were estimated to be ∼364, 388, 365 and 384 kDa, respectively, suggesting that all three AsphyA kinase mutants existed as dimers. Furthermore, we confirmed that all three mutants bound to PIF3 in a Pfr-specific manner, similar to WT AsphyA (Fig. 4b). Finally, we performed the photoaffinity labelling experiments with 8-azido-ATP, which suggest that ATP-binding affinity was significantly decreased in all three kinase mutants (Fig. 4c, Supplementary Fig. 15b). Comparing the labelling with 5 μM 8-azido-ATP, K411L showed ∼30% labelling, T418D showed ∼17%, and D422R showed ∼5% of the WT AsphyA. Moreover, we obtained the *K*_d_ values of ∼1.32 × 10^−6^ M for K411L and 4.76 × 10^−6^ M for T418D (Supplementary Fig. 12). In the case of D422R, we could not obtain the *K*_d_ value at micromolar level, possibly due to too low ATP binding. These results of *K*_d_ values were well-correlated with the reduced kinase activities of AsphyA mutants. Collectively, these results suggest that the kinase mutants obtained in this study have a reduced binding affinity to ATP, which might explain why these mutants exhibited reduced kinase activities.

In addition to full-length AsphyA, we confirmed that the D422R mutant of A610 (DR/A610) had significantly reduced autophosphorylation and PIF3 kinase activities with reduced 8-azido-ATP labelling (Supplementary Fig. 15c,d). These results indicate that the mutation causing impaired kinase activity in full-length AsphyA was also effective within the N-terminal region only. However, we could not rule out the importance of the C-terminal region for the kinase activity, since deletion of the C-terminal region increased autophosphorylation and PIF3 kinase activities (Fig. 3b,c). Indeed, the autophosphorylation and PIF3 kinase activities of DR/A610 were higher than those of the D422R mutant of full-length AsphyA.

### Light responses in plants expressing mutant AsphyA

**Figure 5:**
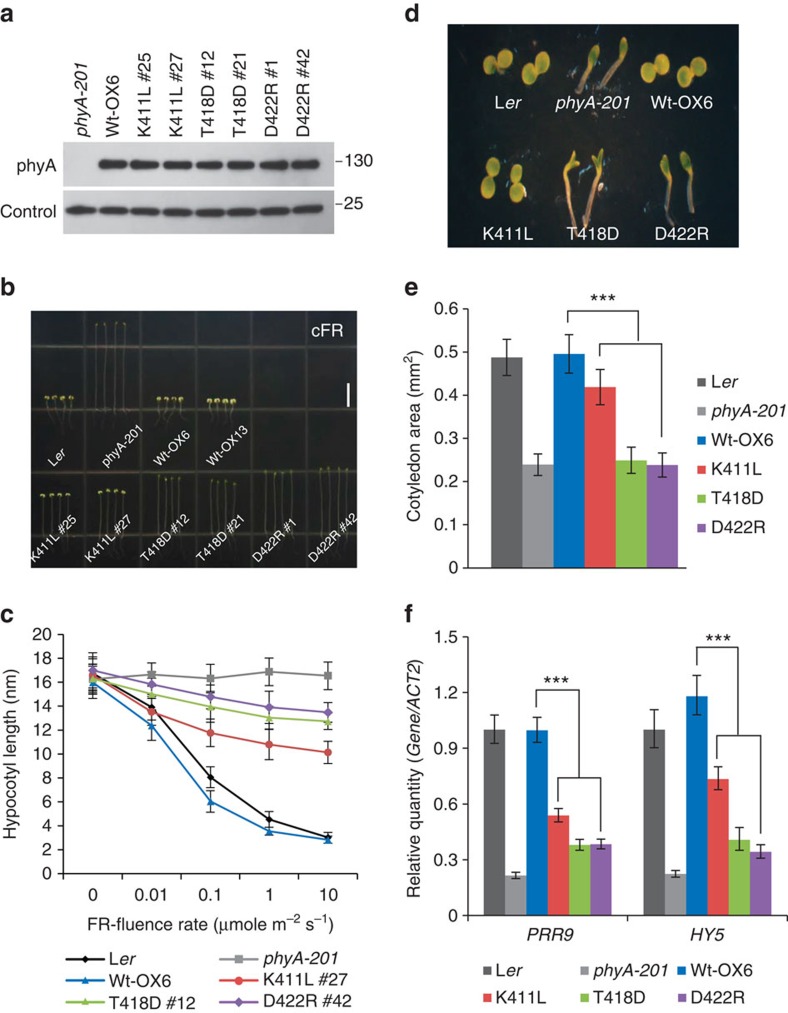
Photoresponse analyses of transgenic *phyA-201* plants with AsphyA kinase mutants under far-red light. (**a**) Western blot analysis to show the protein levels of AsphyA in transgenic plants. Loading controls are shown in the lower panel. *phyA-201*, phyA-deficient *Arabidopsis thaliana* (L*er* ecotype); WT-OX6, transgenic *phyA-201* plant with WT AsphyA; K411L, T418D and D422R, transgenic *phyA-201* plants with corresponding AsphyA kinase mutant. (**b**) Hypocotyl de-etiolation of representative seedlings grown for 5 days under cFR light condition (10 μmol m^−2^ s^−1^). Scale bar, 5 mm. (**c**) Far-red fluence-rate response curves for the inhibition of hypocotyl growth. Data are expressed as means±s.d. (*n*≥30). (**d**) Cotyledon expansion of 4-day-grown seedlings under cFR. (**e**) Comparisons of cotyledon areas. Data are expressed as means±s.d. (*n*≥15). Statistically significant changes compared with WT-OX6 are indicated (***P<0.001, as determined using Tukey's test). (**f**) Quantitative RT-PCR analysis of *PRR9* and *HY5* in 3-day-old etiolated seedlings exposed to 2 h far-red (10 μmol m^−2^ s^−1^). Statistically significant changes compared with WT-OX6 are indicated (***P<0.001, Tukey's test, *n*=3 replicates).

To investigate the potential *in vivo* functional roles of phytochrome kinase activity, we produced transgenic lines (*phyA-201* background) expressing *AsphyA* kinase mutant genes under the control of the 35S promoter. We then selected transgenic lines showing a comparable level of AsphyA protein expression to that of a WT AsphyA transgenic line (WT-OX6) (ref. 23) for further analysis (Fig. 5a). Since phyA is known to participate exclusively in the far-red-induced inhibition of hypocotyl elongation^31^, we investigated the seedling de-etiolation responses in continuous far-red light (cFR). Hypocotyl lengths of all the transgenic lines expressing the AsphyA kinase mutants were longer than those of both WT *Arabidopsis* (L*er*) and transgenic lines with WT AsphyA (WT-OX6 and -OX13; Fig. 5b,c). Transgenic lines with D422R showed the longest hypocotyls, while K411L transgenic lines showed shorter hypocotyls than D422R and T418D transgenic lines, but still much longer than L*er* and WT-OXs. T418D transgenic lines showed similarly long hypocotyls to D422R transgenic plants, but they were slightly shorter than the D422R plants. These far-red-sensitivity results correlated to the degree of ATP-binding in the AsphyA kinase mutants, in which D422R had the weakest ATP-binding affinity, followed by T418D and K411L (see Fig. 4c). Moreover, transgenic seedlings with AsphyA kinase mutants showed significantly reduced cotyledon opening and expansion under cFR (Fig. 5d,e). These results suggest that the AsphyA mutants with reduced kinase activity were hypoactive in plants, thus decreasing the sensitivity of the seedlings to far-red.

In addition to the hypocotyl growth response to cFR (that is, a far-red high-irradiance response), we further examined a far-red very low fluence response (FR-VLFR) with hourly 5-min pulses of far-red. The results showed that the transgenic lines with AsphyA kinase mutants exhibited longer hypocotyls than those with L*er* and WT-OX6, in which the FR-VLFR of the transgenic plants were also correlated to the ATP-binding ability of the mutants (Supplementary Fig. 17). These results suggest that the AsphyA kinase activity is important for FR-VLFR as well as far-red high-irradiance response. Moreover, we investigated the expression of photoresponsive genes in the transgenic plants, because phytochromes are known to mediate seedling photomorphogenesis through regulation of gene expression^32^. Since the expression of *PRR9* and *HY5* is rapidly induced by phyA in response to far-red^33^,^34^, we examined the far-red-induced *HY5* and *PRR9* expression and found that their induction were both significantly lowered in the transgenic plants with AsphyA kinase mutants (Fig. 5f). Collectively, these results indicate that the mutations which reduce AsphyA kinase activity diminish far-red signalling, thus suggesting that AsphyA kinase activity plays a positive role in plant light signalling. In addition, our results also suggest a positive relationship between the magnitude of AsphyA kinase activity and far-red photoresponses in plants.

### Far-red light-induced phosphorylation and degradation of PIF3

It is well-known that the translocation of phyA into the nucleus is one of the most critical steps for its signalling in plants^4^. Thus, the reduced far-red-responses of transgenic plants with AsphyA kinase mutants could occur as a result of the impaired translocation into the nucleus. To test this hypothesis, we investigated nuclear localization of AsphyA using transgenic *phyA-201* plants expressing eGFP-fused AsphyA kinase mutants. The results showed that all the AsphyA kinase mutants localized to the nucleus in a light-dependent manner, similar to WT AsphyA (Supplementary Fig. 18a). Next, we compared the protein–protein interactions of AsphyA with FHY1 and FHL that participate in the nuclear localization of phyA. All three of the AsphyA kinase mutants interacted with both FHY1 and FHL in a Pfr-specific manner, similar to WT AsphyA (Supplementary Fig. 18b). These results suggest that the nuclear localization of the AsphyA kinase mutants is normal. In addition, decreases in protein stability might be the reason for the reduced far-red-responses. Thus, we investigated light-dependent degradation of the AsphyA kinase mutants in the transgenic plants, and found that the kinase mutants were degraded under white light and far-red at similar rates to WT AsphyA (Supplementary Fig. 18c). Therefore, we suggest that their hyposensitivity to far-red was due to impaired ATP-binding and reduced kinase activity.

**Figure 6:**
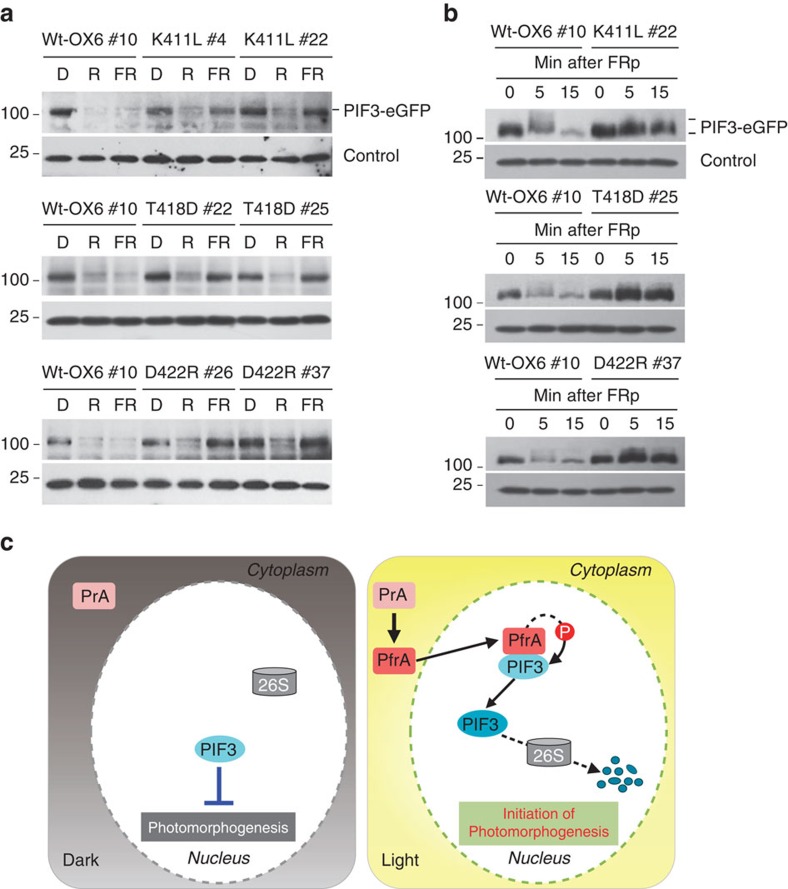
Far-red light-mediated phosphorylation and degradation of PIF3 is prevented in transgenic plants with AsphyA kinase mutants. (**a**) PIF3 degradation under different light conditions. 4-day-old dark-grown seedlings were exposed to either cR (10 μmol m^−2^ s^−1^) or cFR (10 μmol m^−2^ s^−1^) for 30 min and 2 h, respectively. Loading controls are shown in the lower panels. (**b**) Far-red-induced phosphorylation of PIF3. Four-day-old dark-grown seedlings were exposed to FRp (7,500 μmol m^−2^) and incubated in the dark for the time indicated before collecting for protein extraction. (**c**) A proposed model for the function of phyA as a protein kinase. For simplicity, PIF3 and phyA are depicted in this model as monomers, but they exist as dimers. In the dark, PIF3 proteins accumulate in the nucleus and regulate the expression of light-responsive genes to prevent photomorphogenesis. In the light, we propose that PfrA in the nucleus interacts and phosphorylates PIF3 directly, which induces PIF3 degradation via the ubiquitin/26S proteasome protein-degradation pathway. Thus, PIF3 phosphorylation by phyA and its subsequent degradation might induce the signal for the initiation of photomorphogenesis. 26S, 26S proteasome complex; P, phosphate; PfrA, Pfr form of phyA; PrA, Pr form of phyA.

Photoactivated phytochrome is known to induce rapid PIF3 phosphorylation and degradation via the 26S proteasome^19^. Here, we observed that phytochromes support PIF3 phosphorylation *in vitro* (see Fig. 1c,d, Supplementary Fig. 3). Therefore we hypothesized that direct phosphorylation of PIF3 by phytochrome could be responsible for proteasome-mediated degradation of PIF3. To examine this possibility, we obtained transgenic lines co-expressing eGFP-fused PIF3 and either WT AsphyA (PIF3/WT-OX6) or one of the AsphyA kinase mutants (PIF3/K411L, PIF3/T418D or PIF3/D422R). We then selected transgenic lines that exhibited similar levels of PIF3-eGFP proteins (Supplementary Fig. 19a), and analysed the PIF3 protein levels under red or far-red light. Consistent with a previous report^19^, we detected PIF3 degradation in PIF3/WT-OX6 transgenic plants under both red and far-red irradiation (Fig. 6a). In contrast, significant amounts of PIF3 remained in the transgenic plants with PIF3/K411L, PIF3/T418D and PIF3/D422R under far-red light. Under red light, PIF3 was degraded almost completely in all the transgenic plants. Altogether, our results suggest that AsphyA kinase mutants are less effective than WT AsphyA in targeting the degradation of PIF3 under far-red light.

To verify whether the prevention of far-red-induced PIF3 degradation in the transgenic plants correlates with reduced phosphorylation, we further investigated PIF3 phosphorylation *in vivo*. The results showed that pulsed far-red (FRp) induced a mobility shift of PIF3 within 5 min, possibly due to multiple phosphorylation, and PIF3 degradation progressed rapidly in PIF3/WT-OX6 plants (Fig. 6b). In contrast, both the mobility shift and subsequent degradation of PIF3 were significantly prevented in transgenic plants expressing the AsphyA kinase mutants. When we examined the mobility shift of PIF3 under red light conditions, all the transgenic plants with PIF3/AsphyA kinase mutants as well as PIF3/WT-OX6 showed similar shifts of PIF3 bands (Supplementary Fig. 19b). To further examine whether the mobility shift was due to the phosphorylation of PIF3, CIAP (calf intestinal alkaline phosphatase) was treated to PIF3 protein sample that was prepared from FRp-exposed seedling extracts by immunoprecipitation with anti-GFP antibody. The result showed that the mobility-shifted PIF3 bands were eliminated in the sample treated with CIAP (Supplementary Fig. 20a), suggesting that the slow-migrating bands were due to its phosphorylation. In addition, we investigated the phosphorylation and degradation of PIF3 with the treatment of a proteasome inhibitor, MG132. As expected, the PIF3 mobility shift was clearly observed in the PIF3/WT-OX6 plant, whereas the shift was barely detected in the transgenic plants of PIF3/T418D or PIF3/D422R (Supplementary Fig. 20b,c). In the case of the PIF3/K411L plant, a slight shift of PIF3 was observed. When we compared the degree of PIF3 degradation, ∼73% of PIF3 had been degraded in the FRp-treated PIF3/WT-OX6 plant after 15 min dark incubation, while little PIF3 degradation was observed in the PIF3/T418D or PIF3/D422R plants and about 30% of PIF3 had been degraded in the PIF3/K411L plant (Supplementary Fig. 20b). Collectively, our results provide evidence that AsphyA can function as a far-red-responsive protein kinase that triggers PIF3 phosphorylation and its subsequent degradation.

## Discussion

There are >20 known signalling components that physically interact with phytochromes, and phytochromes are believed to regulate downstream processes by protein–protein interactions^3^. Because it has been proposed that phytochromes have kinase activity, it is possible that some of these phytochrome-interacting proteins could be phosphorylated by phytochromes. Here, we provide evidence that PIFs are phosphorylated directly by phytochromes *in vitro* and that physical interaction of PIFs with phytochromes is critical for this phosphorylation (Figs 1 and 2). This is consistent with the previous results that direct physical interaction with photoactivated phytochromes is necessary for phosphorylation of PIFs *in vivo*^19^,^21^. Importantly, we found that autophosphorylation of phytochromes, including AsphyA, AtphyB and AtphyD, was significantly reduced in the presence of PIF3 (Fig. 1c,d). Furthermore, the addition of PIF3 also reduced histone H1 phosphorylation by AsphyA (Supplementary Fig. 3a). This suggests PIF3 as the preferred substrate of phytochromes.

There are concerns about contaminating protein kinase(s) in the purified recombinant protein samples. However, several results of the present study support that the kinase activity originates from phytochromes. First, histone H1 sample used in the phosphorylation assays had no detectable kinase activity (Supplementary Fig. 1). Second, no phosphorylation was observed in reactions that only contained PIF proteins (Fig. 1c,d, Supplementary Fig. 5). There is another possibility that a kinase could be co-purified with phytochromes, especially when expressed in yeast. However, photoaffinity labelling experiments with the phytochrome samples purified from *P. pastoris* did not show any contaminating 8-azido-ATP-labelled protein except AsphyA (Supplementary Fig. 10). In addition, not all of the AsphyA proteins exhibited the kinase activity (Fig. 4, Supplementary Fig. 14). More importantly, A610 purified from *E. coli* exhibited the kinase activity on PIF3 (Supplementary Fig. 8). In the *E. coli* genome, four putative protein kinases have been reported^35^, but no homologue exists in either yeast or higher eukaryotes, including plants. Thus, we believe the likelihood that a similar kinase was purified from both *P. pastoris* and *E. coli* is probably very small. We also detected no contaminating protein kinase in the phycocyanobilin sample (Supplementary Fig. 9). Finally, we showed that apo-proteins of the photosensory core did not phosphorylate PIF3, whereas holo-proteins did (Fig. 3f). Therefore, it is unlikely that a contaminating kinase is associated with phytochromes, so we believe that the observed kinase activity originates from phytochrome itself.

Using PIF3 as a substrate, we demonstrate that the PAS-GAF-PHY photosensory core retains the observed kinase activity (Fig. 3). Moreover, in addition to 8-azido-ATP labelling (Supplementary Fig. 11), our results show that the photosensory core possesses an ATP-binding site and a *K*_d_ value of 0.72 μM, which is lower than that of full-length AsphyA (0.87 μM; Supplementary Fig. 12). These results suggest that the photosensory core is the kinase domain in AsphyA. The tertiary structure of the cyanobacterial phytochrome Cph1 suggests that the photosensory core region is similar to the regulatory region of cyclic nucleotide phosphodiesterases and adenylyl cyclases^36^. These proteins utilize nucleoside phosphates for their reactions, thus supporting a chance of ATP-binding in the photosensory core region. Indeed, we could suggest a putative ATP-binding site in the PHY domain of the photosensory core by homology modelling using the crystal structures of AtphyB (PDB code: 4OUR;) (ref. 37) and PaBphP (PDB code: 3C2W) (ref. 38) as templates (Supplementary Note 4, Supplementary Figs 21–26 and Supplementary Table 1).

In the present study, we obtained three AsphyA mutants with impaired kinase activities (Fig. 4, Supplementary Fig. 14), and confirmed that the transgenic *phyA-201* plants with these kinase mutants showed hyposensitive responses to far-red light (Fig. 5, Supplementary Fig. 17). To identify the reason for far-red hyposensitivity in the transgenic plants, we confirmed no obvious defects of the AsphyA kinase mutants in photochemical properties including dark reversion, dimerization, interaction with downstream components, nuclear localization and protein stability (Fig. 4b, Supplementary Figs 16 and 18). Thus, we propose that the hyposensitive responses of the transgenic plants to far-red are due to impaired ATP-binding of the kinase mutants (Fig. 4c, Supplementary Figs 12 and 15b). Moreover, we confirmed that far-red-induced PIF3 degradation and phosphorylation was significantly inhibited in transgenic plants expressing AsphyA kinase mutants (Fig. 6a,b and Supplementary Fig. 20b,c). In contrast, red-induced PIF3 degradation and phosphorylation occurred normally (Supplementary Fig. 19b), which might be due to the activity of the other phytochromes (phyB-E). Therefore, our results suggest that far-red-induced PIF3 phosphorylation and degradation were directly related to the kinase activity of AsphyA, and that AsphyA regulates far-red-induced PIF3 degradation via direct phosphorylation *in vivo* by its kinase activity.

When comparing the results of *in vitro* and *in vivo* phosphorylation of PIF3, we noted that the slow-migrating PIF3 proteins due to multiple phosphorylation were observed in far-red- and red-treated plants (Fig. 6b, Supplementary Figs 19b and 20), but not *in vitro* (Fig. 1). This suggests that phytochromes alone are not enough to mediate the multiple phosphorylation of PIF3 and that, although phytochromes may be able to initiate PIF3 phosphorylation, additional protein kinase(s) such as casein kinase II (ref. 39) might be necessary to induce multiple phosphorylation of PIF3. More recently, the N-terminal domain of AtphyB (that is, NG-GUS-NLS containing B651) has shown to mediate PIF3 phosphorylation but not its degradation, suggesting that C-terminal domain is necessary for the PIF3 degradation^40^. The B651 mutant also showed much less multiple phosphorylation of PIF3 than full-length AtphyB. Thus, the C-terminal domain of AtphyB is likely to be required for the light-dependent multiple phosphorylation of PIF3, despite being dispensable for the induction of light responses^41^. In this regard, an interesting result of the present study is that the C-terminal domain-deleted mutants displayed significantly increased autophosphorylation and kinase activities (Fig. 3, Supplementary Fig. 7), suggesting that the C-terminal domain might play a role in regulating phytochrome kinase activity. Since the observed kinase activity of phytochromes can be stimulated by other factors, such as histone H1, it is possible that the kinase activity might be regulated through the conformational changes in the C-terminal domain caused by the interaction with signalling components, such as PKS1 and phytochrome-associated protein phosphatase 5 (ref. 42) that are reported to interact with the HKRD of phytochromes. It is also possible that the C-terminal domain promotes PIF3 degradation by recruiting additional protein kinase(s) or ubiquitin E3 ligase(s), as has been suggested previously^40^. Indeed, there was a recent report that light-induced phosphorylation of PIF3 is necessary for the degradation of the PIF3-AtphyB complex via recruitment of LRB (light-response Bric-a-Brack/Tramtrack/Broad) E3 ubiquitin ligases^43^. However, further studies will be necessary to elucidate the potential functional role of the C-terminal domain in regulating phytochrome kinase activity.

In combination with previous reports, our results provide a possible model for the functional role of phyA kinase activity on PIF3 (Fig. 6c). In the dark, phyA proteins are synthesized and accumulated as the Pr forms in the cytosol, while PIF3 proteins are accumulated in the nucleus and prevent photomorphogenesis by regulating the expression of light-responsive genes. On illumination, the Pr forms of phyA are photoactivated to the Pfr forms that translocate from the cytosol into the nucleus. Considering our findings suggest that AsphyA protein kinase activity is not light-regulated, we believe that the light-regulated nuclear localization and/or light-regulated interaction with substrates are important steps for the phytochrome function. Once localized in the nucleus, phyA may be able to interact and phosphorylate PIF3 directly, which induces PIF3 degradation via the ubiquitin/26S proteasome pathway. This PIF3 phosphorylation by phyA and its subsequent degradation may then contribute to the initiation of photomorphogenesis. Therefore, the protein kinase activity of phyA might play a crucial role in controlling the number of active PIF3 to elicit plant light signalling.

## Methods

### Preparations of recombinant phytochrome proteins

Full-length phytochrome genes of *AsPHYA*, *Brachypodium distachyon* phyA (*BdPHYA*), *Pisum sativum* phyA (*PsPHYA*), *Arabidopsis thaliana* phyA (*AtPHYA*), *AtPHYB* and *AtPHYD* were cloned into pPIC3.5K (Invitrogen)^44^ for the expression of recombinant phytochrome proteins. In addition, A875 (1–875aa), A610 (1–610aa), A407 (1–407aa), Δ65 (66–1129aa), AC (572–1129aa) and the PAS-GAF-PHY photosensory core (66–610aa) were also cloned into pPIC3.5K for the kinase-domain-mapping experiments. To generate phyA mutants with impaired kinase activity, mutagenesis was performed with full-length AsphyA as a template using the QuickChange Site-Directed Mutagenesis System (Stratagene) and mutagenic primers. The site-mutants, B651 (1–651aa of AtphyB) and DR/A610 (D422R mutant of A610) were also cloned into pPIC3.5K for the expression of recombinant proteins. The primers used for these constructs are all shown in Supplementary Table 2.

The *Pichia pastoris* protein-expression system (Invitrogen) and streptavidin affinity chromatography (IBA) were used to express and purify recombinant phytochrome proteins. A ten-amino acid streptavidin-affinity tag from pASK75 vector (Biometra)^45^ was attached to the 3′-end of the phytochrome genes which were subcloned into pPIC3.5K vector (Invitrogen). The pPIC3.5K constructs with phytochrome genes were transformed into *P. pastoris* GS115 cells by electroporation. Recombinant phytochrome proteins were then expressed in *P. pastoris* cells and purified using the streptavidin-affinity chromatography, according to the manufacturer's recommendations. Briefly, *P. pastoris* cells grown in MGY media (1.34% yeast nitrogen base, 4 × 10^−5^% d-biotin and 1% glycerol) were collected and transferred to MM media (1.34% yeast nitrogen base, 4 × 10^−5^% d-biotin and 0.5% methanol) with the final OD_600_=0.8–1.0. The cells were then incubated for 24 h at 30 °C with vigorous shaking. After collecting *P. pastoris* cells, crude extracts were prepared by breaking cells in liquid nitrogen using a homogenizer (Nihonseiki Kaisha). Phytochrome samples were precipitated by adding 0.23 g ml^−1^ of ammonium sulfate, and resuspended in a TE buffer (100 mM Tris-HCl, pH 7.8 and 1 mM EDTA). For *in vitro* reconstitution to generate chromophore-assembled holo-phytochrome proteins, phycocyanobilin was prepared from lyophilized *Spirulina* cells by methanolysis. The lyophilized cells were resuspended into water, and the supernatant obtained by centrifugation at 8,000*g* for 15 min was mixed with trichloroacetic acid (1%, w/v). After incubation for 1 h at 4 °C in darkness, the collected precipitate was washed three times with methanol and resuspended in methanol (12 ml per 3 g of cells) containing 1 mg ml^−1^ HgCl_2_. After incubation for 24 h at 40 °C in darkness, HgCl_2_ was removed by addition of β-mercaptoethanol (1 μl ml^−1^) and the bilin-containing methanol solution was obtained by centrifugation at 8,000*g* for 5 min. After extraction with chloroform and evaporation, the phycocyanobilin was dissolved in DMSO and stored at −80 °C until use. The concentration of phycocyanobilin was quantified by absorption spectroscopy in HCl (2%)/methanol using the extinction coefficient of 37,900 M^−1^ cm^−1^ at 690 nm. For chromophore-assembled holo-phytochromes, phycocyanobilin was added to the ammonium sulfate-precipitated phytochrome samples at a final concentration of 10 μM, and the mixture was incubated on ice for 1 h. After dialysis to remove free chromophores, the samples were loaded to the streptavidin affinity chromatography and recombinant phytochrome proteins were eluted with the TE buffer containing 5 mM desthiobiotin.

We also purified the recombinant A610 protein from *E. coli*. For this, the A610 gene with the streptavidin affinity-tag at the 3′-end was PCR-amplified with 5′-CCGCTCGAGATGGATGCTATTCATTCATTGC-3′ (forward, *Xho*I) and 5′-ATATGCGGCCGCTTAACCACCGAACTGCGGGTGACGCCA-3′ (reverse, *Not*I) using pPIC3.5K-A610/strep as a template. The PCR product was then subcloned into an *E. coli* expression vector pBAD-MycHisC (Invitrogen) to produce pBAD-A610/strep. *E. coli* strain LMG194 (Invitrogen) was transformed with the pBAD-A610/strep to express A610 apo-protein or co-transformed with pPL-PCB (ref. 46) to produce phycocyanobilin-assembled A610 holo-protein. Recombinant expression of phycocyanobilin-assembled A610 protein was induced by adding 1 mM IPTG and 0.002% (w/v) arabinose in *E. coli*, and A610 protein was purified by the streptavidin-affinity chromatography.

### Preparation of phytochrome-interacting proteins

For recombinant phytochrome-interacting proteins including PIF1, PIF3, Δ210-PIF3 (that is, a phytochrome-binding motif-deleted PIF3 mutant), PIF4, PIF7, PKS1, FHY1 and FHL, and the glutathione *S*-transferase and streptavidin-affinity tags (GST/strep) were fused to their N- and C-termini, respectively. They were subcloned into pGEX 4T vector (GE Healthcare) with primer sets (Supplementary Table 2), and transformed into *E. coli* strain BL21 or BL21-CodonPlus (Invitrogen). For the induction of recombinant phytochrome-interacting proteins, *E. coli* cells grown in LB medium containing 50 μg ml^−1^ of ampicillin were transferred to RB medium (0.5% yeast extract, 1% tryptone, 0.5% NaCl, 0.2% glucose, pH 7.5) supplemented with antibiotics and incubated at 37 °C until OD_600_ reached ∼0.6. Expression of the recombinant proteins was then induced by adding IPTG to a final concentration of 1 mM, and the culture was further incubated for 3–4 h at 37 °C. After centrifugation, the cell pellet was resuspended in ice-cold TE buffer in a ratio of 2 ml g^−1^ of *E. coli* cells (wet pellet) and lysed by repeated sonication. After the homogenates were clarified by centrifugation, the supernatant was passed through a 0.45 μm microfilter (Corning) to remove any insoluble particles, and loaded onto the streptavidin-affinity chromatography for purification. The concentrations of recombinant proteins were determined using a Quant-iT Protein Assay Kit (Invitrogen).

### Zinc fluorescence and spectroscopic analysis

For zinc-fluorescence assays to assess the chromophore ligation, the phytochrome samples were analysed on SDS-polyacrylamide gels and the gels were then soaked in 20 mM zinc acetate/150 mM Tris-HCl (pH 7.0) for 5–30 min at room temperature with gentle shaking. Zinc fluorescence of holo-phytochromes was then visualized under ultraviolet light (312 nm). All of the spectroscopic experiments were carried out under the green safety light condition, which consisted of a white fluorescent lamp equipped with a plastic filter (Rosco) with a maximal transmittance at 500 nm. Absorption spectra were recorded by a diode array ultraviolet /visible spectrophotometer (Cary) after red or far-red light irradiation. A fibre optic illuminator system (Cole-Parmer) equipped with 656 and 730 nm interference filters (Oriel) was used as a light source, and the light intensity was 8 W m^−2^ for red light and 6 W m^−2^ for far-red light. The samples were illuminated with red or far-red light for 15 min. A differential spectrum was obtained by subtracting the Pfr spectrum from the Pr spectrum. The concentrations of the holo-phytochrome samples were calculated from Pr absorbance peak with an extinction coefficient of 132,000 M^−1^ cm^−1^. To examine non-photochemical reversion of Pfr to Pr (that is, dark reversion), phytochromes were phototransformed to Pfr forms by irradiation with red light for 15 min and the amounts of Pfr (%) were estimated from the absorbance peak in the dark for 120 min relative to original Pfr peak as 100%.

### Phytochrome autophosphorylation and kinase assays

For autophosphorylation assays, reaction mixtures (20 μl) contained kinase buffer (25 mM Tris-HCl, pH 7.8, 0.2 mM EDTA, 4 mM DTT and 5 mM MgCl_2_) and purified recombinant phytochromes (0.5–1.0 μg) in either Pr or Pfr forms. Phytochrome samples were irradiated with red or far-red light for 15 min before the start of the reaction. Because histone H1 is known to stimulate phytochrome autophosphorylation activity^23^, the same amount of histone H1 (0.5–1.0 μg) was added to the phytochrome samples in the indicated experiments. The conditions for kinase assays were similar to those of phytochrome autophosphorylation experiments, except for addition of a substrate (0.5–1.0 μg). Reactions were started by adding 150 μM ATP containing 10 μCi of (γ-^32^P) ATP and incubated at 30 °C for 1 h. Proteins were resolved on SDS-polyacrylamide gels and dried under vacuum before autoradiography by exposing on x-ray films. To verify the proteins loaded, Coomassie blue protein staining and zinc-fluorescence assay were performed before drying. For quantification of the phosphorylation, dried gels were exposed to Fuji imaging plates, and the relative signals of phyA autophosphorylation and PIF3 phosphorylation by phyA were quantitated with a Typhoon FLA 7000 phosphor-imager (GE Healthcare).

### *In vitro* protein–protein interaction assay

To examine the protein–protein interaction between phytochromes and phytochrome-interacting proteins *in vitro*, pull-down experiments were performed. Phytochromes (1.0 μg) and GST/strep-fused phytochrome-interacting proteins (1.0 μg) were incubated for 60 min at 4 °C in 500 μl of pull-down buffer (100 mM Tris-HCl, pH 7.8, 1 mM EDTA, 150 mM NaCl and 100 μg ml^−1^ BSA). Glutathione resin was then added and incubated for an additional 30 min. Phytochromes and GST/strep-fused phytochrome-interacting proteins in the supernatant and precipitate were detected using an ECL western blotting analysis system (Thermo Scientific) with 1:5,000 oat phyA-specific monoclonal antibody (oat22; ref. 47) and 1:2,000 GST-specific antibody (sc-138, Santa Cruz Biotechnology), respectively.

### Photoaffinity labelling of phyA with an azido-ATP analogue

To investigate ATP-binding to AsphyA proteins, photoaffinity labelling experiments were carried out using 8-N_3_-ATP-biotin-long chain-hydrazone (8-azido-ATP), which were purchased from Affinity Labeling Technologies, Inc. Purified phytochrome proteins (1.0 μg) were pre-incubated for 30 min on ice in 50 μl of photoaffinity labelling buffer (20 mM Tris-HCl, pH 7.0, 2 mM EDTA, 2 mM MgCl_2_ and 150 μM ATP), and then 8-azido-ATP was applied with indicated concentrations. After incubation for 5 more min on ice, ultraviolet light at 254 nm was then irradiated for 90 s using a hand-held ultraviolet lamp at 4 cm from the surface of the reaction mixtures. Immediately after photoaffinity labelling, reactions were stopped by adding SDS sample buffer, and the 8-azido-ATP-labelled phytochromes were detected with 1:2,000 avidin-HRP (A-115, Boston Biochem). After development, the images were scanned and opened by ImageJ, a Java-based image-processing software, to quantitate the photoaffinity labelling with azido-ATP analogues. Background signal was subtracted before normalization. The 8-azido-ATP analogue labelling signals were normalized to AsphyA protein levels, and the percentages of 8-azido-ATP labelling were calculated as the 8-azido-ATP labelling of WT AsphyA with 5 μM 8-azido-ATP.

### Determination of binding parameters of ATP in AsphyA

To estimate dissociation constant (*K*_d_) and the number of binding sites (*n*) of ATP in AsphyA proteins, an ATP analogue BODIPY-FL-ATP (BODIPY FL 2′-(or-3′)-O-(*N*-(2-aminoethyl)urethane) adenosine triphosphate; Molecular Probes) was used. 0.5 μM AsphyA proteins were incubated with increasing concentration of BODIPY-FL-ATP (0.5–5 μM) for 10 min at 30 °C, and free (unbound) ATP analogue was separated from the ATP analogue-protein complex by using Amicon Ultra Centrifugal Filter with MWCO of 10,000 Da for 5 min at 4,000*g*. The spectrophotometric absorbance was then measured to determine the BODIPY-FL-ATP concentrations within flow-through and the complex solutions by using Shimadzu UV-1800 spectrophotometer. As a control experiment, the same procedure described above was performed in the absence of protein to test the possibility that BODIPY-FL-ATP could bind to membrane filter, which did not show any measurable amount of the membrane-bound BODIPY-FL-ATP. The concentrations of BODIPY-FL-ATP were also double-checked by measuring the emission intensity of BODIPY fluorophore at the excitation wavelength of 500 nm and emission wavelength of 515 nm. The fluorescence of free BODIPY-FL-ATP in the absence of any protein component was used as a linear standard curve. The *K*_d_ and *n* values were then calculated by a Scatchard plot of *r*/[BODIPY-FL-ATP]_free_ versus *r*, where *r* is the molar ratio of [BODIPY-FL-ATP]_bound_ to [AsphyA]_total_.

### Photoresponse analyses of transgenic *Arabidopsis* plants

Full-length *AsPHYA* mutant (*K411L*, *T418D* and *D422R*) genes were cloned into pBI121, and each construct was introduced into phyA-deficient *Arabidopsis* (*phyA-201*). After obtaining the homozygous lines, western blot analysis was carried out with crude extracts from 5-day-old dark-grown seedlings to assess the expression of AsphyA proteins using AsphyA-specific monoclonal antibody (oat22). For loading controls in this analysis, we used 1:10,000 polyclonal antibody against *Arabidopsis* translationally controlled tumour protein (AtTCTP; At3g16640) (ref. 48). For the photoresponse analyses of seedlings, seeds were sown on half strength MS medium and cold treated for 5 days in the dark. Hypocotyl lengths and cotyledon areas were then measured with 5- or 4-day-grown transgenic seedlings under continuous far-red light (cFR) with various fluence rates or at 10 μmol m^−2^ s^−1^, respectively. In addition, for the analysis of a FR-VLFR, seeds were exposed to red light (20 μmol m^−2^ s^−1^) for 1 h and transferred to dark condition at 21 °C for 1 day. Then, the seedlings were grown with hourly 5 min pulse of far-red light (0.5 μmol m^−2^ s^−1^) or in the dark for 3 days. The hypocotyls and cotyledons were photographed with a digital camera (Nikon), and then measured with Scion Image analysis software.

### Gene expression analysis

Total RNA was extracted from 3-day-old etiolated seedlings exposed to 2 h far-red (10 μmol m^−2^ s^−1^) and quantitative real-time RT-PCR analysis was carried out using Stratagene Mx3005P with Brilliant III Ultra-Fast SYBR Green Q-PCR Master Mix (Agilent Technologies). The expression of *PRR9* and *HY5* was normalized to the expression of *ACT2*. Primers used in this analysis are 5′-GCCAGAGAGAAGATGCATTGA-3′ and 5′-CCTGCTCTGGTACCGAACCTT-3′ for *PRR9*; 5′-CAAGCAGCGAGAGGTCATCA-3′ and 5′-ATCGCTTTCAATTCCTTCTTTGA-3′ for *HY5;* 5′-TCGGTGGTTCCATTCTTGCT-3′ and 5′-GCTTTTTAAGCCTTTGATCTTGAGAG-3′ for *ACT2*.

### Confocal microscopy analysis

An eGFP (enhanced GFP) expression cassette under the control of CsVMV (cassava vein mosaic virus) promoter from pCsVMV-eGFP-N-999 vector was cloned into pBI121 using *Hind*III and *Pvu*II to generate pBI121-eGFP vector. Then, WT and kinase mutant genes of AsphyA were subcloned into the pBI121-eGFP vector using *Eco*RI and *Bam*HI. After confirmation of the constructs by DNA sequencing, all the constructs were transformed into *phyA-201.* To investigate the nuclear localization of AsphyA kinase mutants, 4-day-old dark-grown seedlings were kept in the dark or exposed to white light for 5 min just before the nuclear localization analysis. The seedlings were then transferred onto a microscope slide immersed under a cover slip and observed using a Laser Scanning Confocal Microscope (Leica TCS SP5 AOBS/Tandem) at the Korea Basic Science Institute, Gwangju Center.

### *In vivo* phytochrome-degradation assay

The transgenic plant seeds were germinated, and grown for 4 days in the dark. Seedlings of each transgenic plant were exposed to continuous white light (150 μmol m^−2^ s^−1^) or far-red light (5 μmol m^−2^ s^−1^) and collected at the indicated times. The collected seedlings were stored in liquid nitrogen and ground using TissueRuptor (Qiagen) in an extraction buffer (70 mM Tris-HCl, pH 8.3, 35% ethylene glycol, 98 mM (NH_4_)_2_SO_4_, 7 mM EDTA, 14 mM sodium metabisulfite, 0.07% polyethyleneimine and protease inhibitors). 40 μg of total protein extracts was used for western blot analysis to detect AsphyA with 1:5,000 oat22 monoclonal antibody.

### Transgenic plants co-expressing AsphyA and PIF3-eGFP

The expression cassette of CsVMV_pro:_*eGFP* was cloned into pCAMBIA3300 to generate pCAMBIA3300-eGFP construct, and *PIF3* was inserted into the pCAMBIA3300-eGFP vector. The construct was then introduced into transgenic plants with WT AsphyA (WT-OX6) or AsphyA kinase mutants, and we obtained transgenic plants co-expressing WT-OX6 and PIF3-eGFP (PIF3/WT-OX6) or AsphyA kinase mutants and PIF3-eGFP (PIF3/K411L, PIF3/T418D and PIF3/D422R). Transgenic lines segregating ∼3:1 for herbicide resistance in the T_2_ generation were selected, and independent homozygous lines of each transgenic plant that showed similar levels of PIF3-eGFP proteins were selected for further analysis by western blot analysis of 5-day-old dark-grown transgenic seedlings with 1:500 GFP-specific antibody (sc-9996, Santa Cruz Biotechnology).

### *In vivo* PIF3 phosphorylation and degradation

Transgenic plants co-expressing WT AsphyA and PIF3-eGFP (PIF3/WT-OX6) or AsphyA kinase mutants and PIF3-eGFP (PIF3/K411L, PIF3/T418D and PIF3/D422R) were obtained by transforming the PIF3-eGFP construct into the transgenic plants with WT or AsphyA kinase mutants. To detect light-induced protein degradation of PIF3, the transgenic lines were grown in the dark for 4 days and transferred the seedlings to 10 μmol m^−2^ s^−1^ of continuous red light (cR, bandwidth=25 nm, *λ*_max_=654 nm) that can activate phyB or 10 μmol m^−2^ s^−1^ of continuous far-red light (cFR, bandwidth=42 nm, *λ*_max_=738 nm) that can activate phyA for 30 min or 2 h, respectively. Proteins were then immediately extracted using an extraction buffer (100 mM Tris-HCl, pH 7.8, 4 M Urea and protease inhibitors). To detect the light-induced phosphorylation of PIF3 in the transgenic plants, 4-day-old dark-grown seedlings were exposed to far-red pulse (FRp; 25 μmol m^−2^ s^−1^ for 5 min, 7,500 μmol m^−2^) and incubated in the dark for the times indicated or red light (10 μmol m^−2^ s^−1^) for 20 min before collecting for protein extraction. 30–60 μg of total proteins were separated on 6% SDS-polyacrylamide gel, and the level of PIF3-eGFP proteins was determined by western blot analysis with 1:500 anti-GFP antibody (sc-9996, Santa Cruz Biotechnology).

To detect far-red-induced phosphorylation of PIF3 more clearly, 4-day-old dark-grown seedlings were pretreated with 50 μM MG132 in liquid GM medium for 4 h, then exposed to far-red pulse (FRp, 7,500 μmol m^−2^) and incubated in the dark for the times indicated before collecting for protein extraction using the extraction buffer containing 20 μM MG132. For CIAP (Roche) treatment, PIF3-eGFP proteins were immunoprecipitated from crude extracts of FRp-exposed seedlings with anti-GFP antibody and protein A/G PLUS-agarose (Santa Cruz Biotechnology), following incubation at 4 °C for 90 min. After washing, the pellets were resuspended in CIAP buffer (100 mM Tris, pH 9.0, 50 mM MgCl_2_, 100 mM NaCl and protease inhibitors) and then treated with 100 units of CIAP at 37 °C for 30 min. Pellets were then boiled in 1 × SDS sample buffer and subjected to western blot analysis.

### Homology modelling and simulations

Homology modelling was carried out to build the three-dimensional (3D) structure of AsphyA. During the development of homology models, sequence database search was carried out with *blastp* and *position-specific iterative basic local alignment search tool* (PSI-BLAST) in NCBI to identify the homologues of AsphyA as structure templates from the PDB. Consequently, the crystal structures of AtphyB (PDB code: 4OUR) (ref. 37) and *Pseudomonas aeruginosa* bacteriophytochrome (PaBphP, PDB code: 3C2W) (ref. 38) were selected as the suitable templates to construct the homology model. From sequence alignments, the amino acid sequence of AsphyA displays 57.7% identity and 75.3% similarity to the AtphyB, and 25.4% identity and 43.3% similarity to the PaBphP. In addition, the sensory module of AtphyB was generated using MODELLER because some missing parts were existed in the template structure. AsphyA and kinase mutant models were then constructed by *Build homology model* protocol within *Discovery studio 3*.5 (DS 3.5).

After having homology models of the photosensory core, molecular docking simulation was carried out to predict the binding mode of ATP into the putative ATP-binding sites in WT and mutant models using *Genetic Optimization for Ligand Docking* (GOLD) 5.1 program that utilizes genetic algorithm to dock ligands into a binding site of enzyme. The putative ATP-binding sites of WT and mutant models were defined by *Define and Edit Binding site* tools in DS 3.5. The conformation of ATP molecule was generated for a set of input ligands by *Generate Conformations* protocol in DS 3.5. Then, generated ATP molecule was used for molecular docking study. A radius was taken as 20 Å around the center of defined binding site of WT and mutant models. The predicted poses were ranked by GOLD fitness score including hydrogen-bonding energy, van der Waals energy and ligand torsion strain. The best-docked poses of compounds were selected on the basis of the GoldScore ranking.

Molecular dynamics (MD) simulations were carried out with WT and mutant systems in the presence of explicit solvent using *Groningen MAchine for Chemical Simulation* (GROMACS) 4.6.6 package. The topology files for ATP and phycocyanobilin were generated using *SwissParam*. To gain a refined side chain orientation of protein and correct arrangement of the atoms, we used CHARMM27 force field. The cubic simulation box which is surrounding the WT and mutant systems was filled with transferable intermolecular potential 3P (TIP3P) water, and the entire systems were neutralized by adding Na^+^ counter-ions by replacing some of the water molecules. The systems were subjected to a steepest descent algorithm to remove possible bad contacts from initial structures until a tolerance of 1,000 kJ mol^−1^. The equilibration phases consist of the two steps. The first phase, NVT equilibration, was carried out 100 ps at 300 K. The temperature was maintained through V-rescale thermostat. In the second step, heavy atoms were restrained and the solvent molecules with counter-ions were allowed to move during the 100 ps under 300 K and normal pressure (1 bar) using Parrinello–Rahman barostat. The production run was carried out for 10 ns under periodic boundary conditions with NPT ensemble. The library of integrated network-based cellular signatures (LINCS) algorithm was used to constrain the bonds between corresponding hydrogen atoms and heavy atoms. From the trajectories during last 4 ns of the MD simulation, the representative structure was selected by clustering analysis.

All of the original scan images of films, gels and immunoblots are shown in Supplementary Fig. 27.

## Additional information

**How to cite this article:** Shin, A.-Y. *et al*. Evidence that phytochrome functions as a protein kinase in plant light signalling. *Nat. Commun.* 7:11545 doi: 10.1038/ncomms11545 (2016).

## Supplementary Material

Supplementary Information Supplementary Figures 1-27, Supplementary Tables 1-2, Supplementary Notes 1-4 and Supplementary References
