# Supplementary material for: Evidence that phytochrome functions as a protein kinase in plant light signalling
Source: Nat Commun. 2016 May 13;7:11545. doi: 10.1038/ncomms11545 (PMC4869175; doi:10.1038/ncomms11545)
Supplement: Supplementary Information — Supplementary Figures 1-27, Supplementary Tables 1-2, Supplementary Notes 1-4 and Supplementary References [file ncomms11545-s1.pdf]

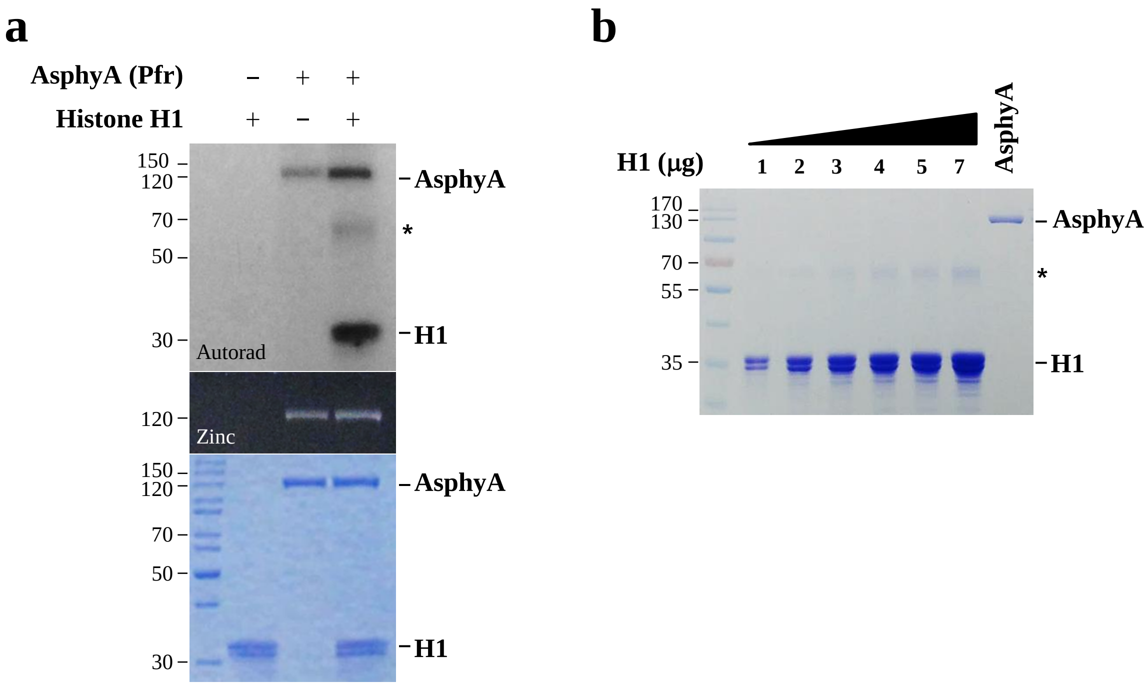

Supplementary Figure 1. Phosphorylation analysis of the histone H1 sample used in this study. (**a**) Phosphorylation of histone H1 in the absence or presence of the Pfr form of AsphyA. Histone H1 was purchased from Roche Korea Co., Ltd. (Cat. no. 10223549001), and phosphorylation assays were conducted with 1 µg histone H1 and 10 µCi [γ-^{32}P]-ATP for 1 h at 30°C. No phosphorylation was detected in the histone H1 sample only, whereas histone H1 was phosphorylated in the presence of AsphyA. PageRuler unstained protein ladder (Thermo Scientific) is included in the first lane of SDS-PAGE gel. (**b**) Coomassie gel showing the protein bands of histone H1. The histone H1 sample showed two major bands near the 35 kDa protein standard and an additional minor band between the 70 and 55 kDa protein standards. The minor band was consistent with the phosphorylated band indicated by an asterisk in (a) (see also asterisks in Fig. 1). AsphyA was included as a control. PageRuler prestained protein ladder (Fermentas) is included in the first lane of the SDS-PAGE gel.

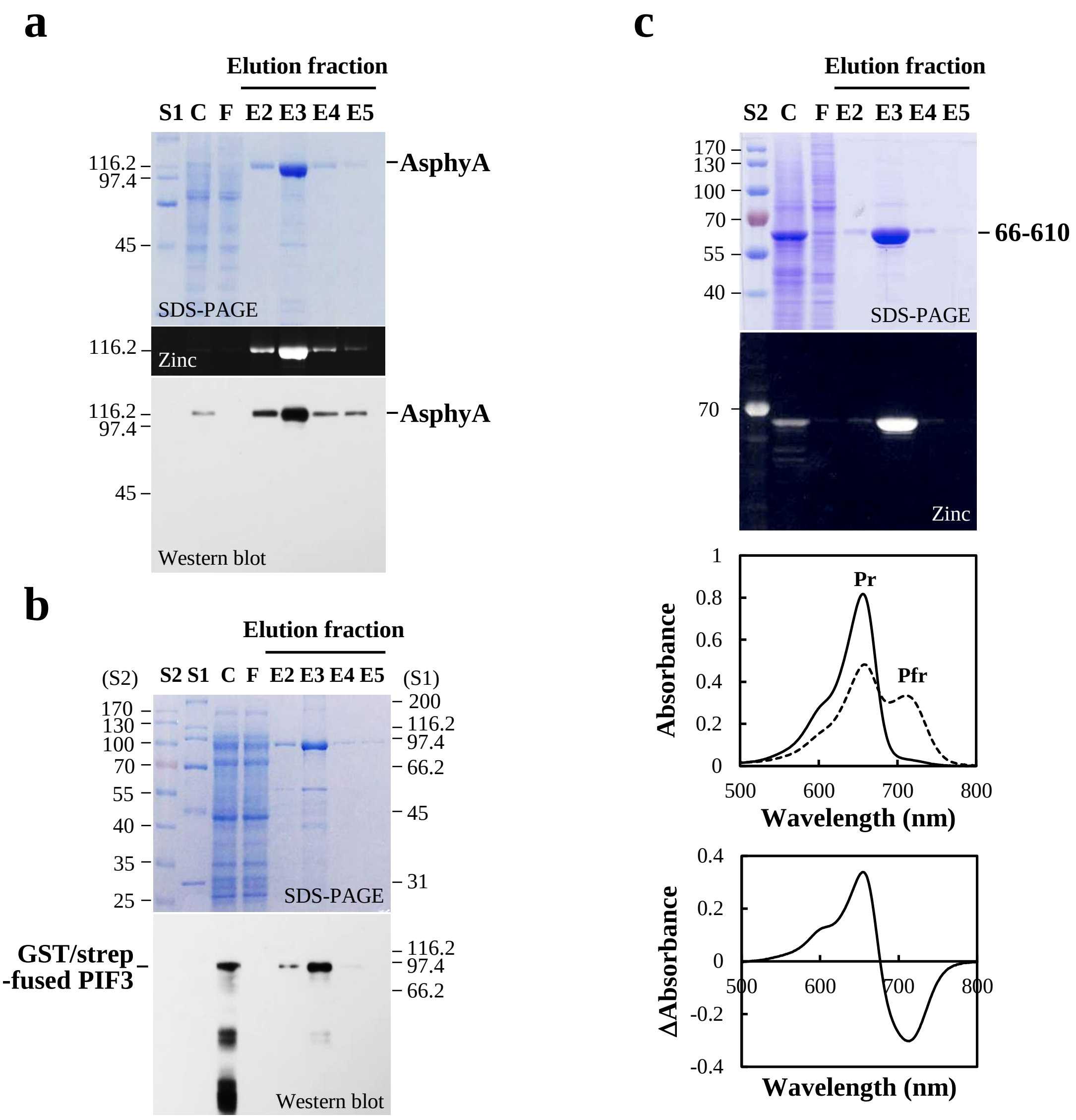

a
Elution fraction
S1 C F E2 E3 E4 E5
116.2
97.4
45
AsphyA
SDS-PAGE
116.2
Zinc
116.2
97.4
45
AsphyA
Western blot
b
Elution fraction
(S2) S2 S1 C F E2 E3 E4 E5 (S1)
170
130
100
70
55
40
35
25
200
116.2
97.4
66.2
45
31
SDS-PAGE
GST/strep
-fused PIF3
116.2
97.4
66.2
Western blot
c
Elution fraction
S2 C F E2 E3 E4 E5
170
130
100
70
55
40
66-610
SDS-PAGE
70
Zinc
Pr
Pfr
Absorbance
Wavelength (nm)
ΔAbsorbance
Wavelength (nm)

Supplementary Figure 2. Purified recombinant AsphyA and PIF3 proteins used in this study. (**a**) Recombinant full-length AsphyA protein purified from *P. pastoris*. Recombinant AsphyA protein with a ten-amino acid streptavidin affinity tag (126.6 kDa) was purified by streptavidin affinity chromatography. Lane S1, Bio-Rad SDS-PAGE standards; lane C, crude extract precipitated with 0.23 g/mL ammonium sulfate; lane F, flow-through of the affinity column; lanes E2-E5, elution fractions of purified AsphyA. Chromophore ligation was confirmed using zinc fluorescence (zinc), and AsphyA protein was detected by western blots with AsphyA-specific (oat22) monoclonal antibody. (**b**) Recombinant GST/strep-fused PIF3 protein purified from *E. coli*. Glutathione *S*-transferase and streptavidin affinity tags (GST/strep) were fused to N- and C-termini of PIF3, respectively. The GST/strep-fused PIF3 proteins (84.3 kDa) were purified by the streptavidin affinity chromatography and detected using western blots with anti-GST antibody. Lane S2, PageRuler prestained protein ladder (Fermentas); Lane C, *E. coli* crude extract containing GST/strep-fused PIF3 proteins; lanes E2-E5, elution fractions of purified GST/strep-fused PIF3. (**c**) Recombinant photosensory core protein of AsphyA (66-610) purified from *P. pastoris*. The absorption (middle) and difference (bottom) spectra of strep-tagged 66-610aa fragments (61.8 kDa) assembled with PCB were included to exhibit phytochrome photoconversion between red-light absorbing Pr and far red-light absorbing Pfr forms. The difference spectrum was obtained by subtracting the Pfr spectrum from Pr spectrum (i.e., ΔAbsorbance).

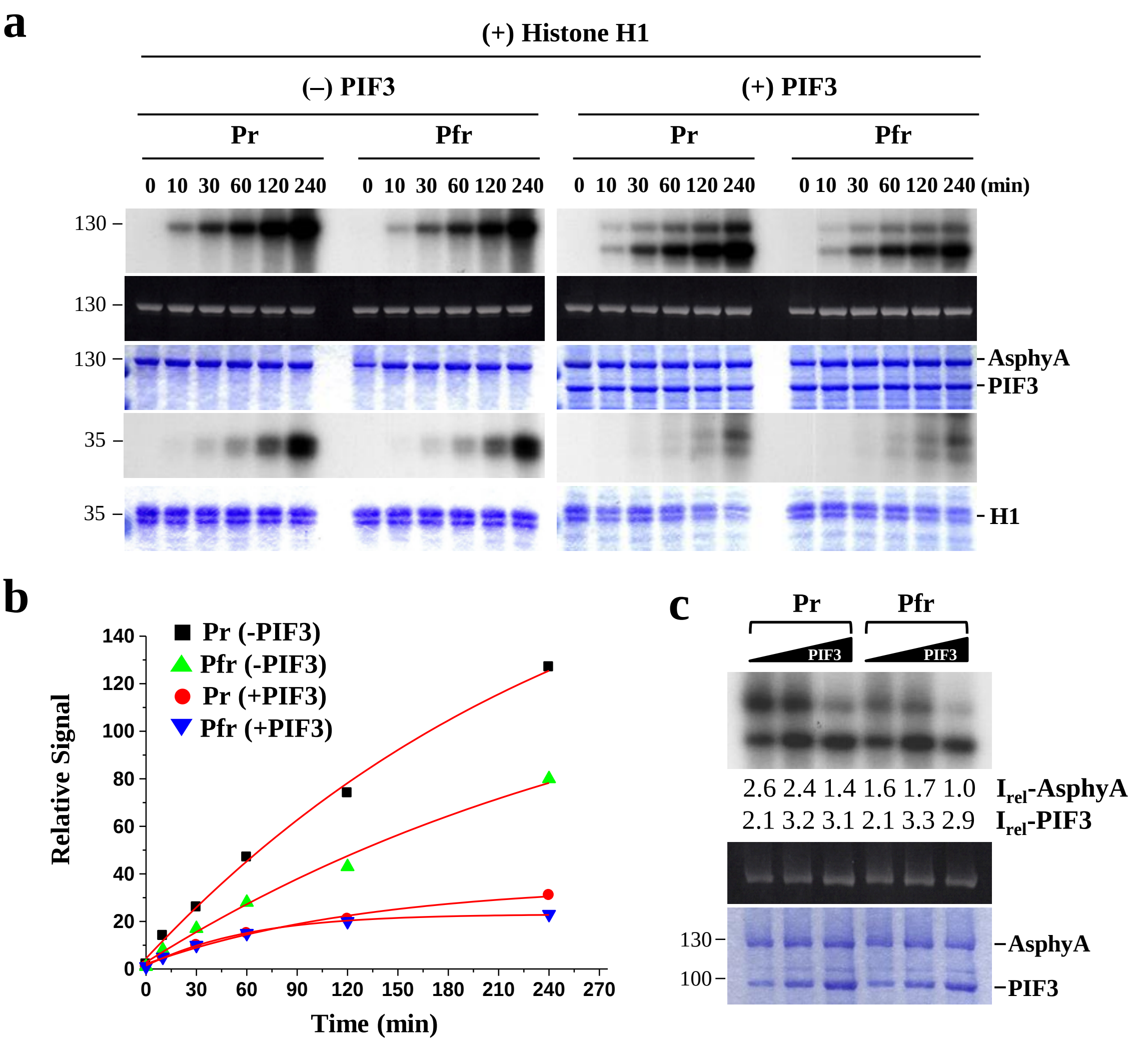

Supplementary Figure 3. *In vitro* phosphorylation of PIF3 by AsphyA. (**a**) Time-dependent kinase activity assays of AsphyA on PIF3. Autophosphorylation and PIF3 phosphorylation by AsphyA in the presence of histone H1 were observed for 240 minutes. (**b**) Comparison of AsphyA autophosphorylation in the absence or presence of PIF3. Relative signals of AsphyA autophosphorylation on (a) were quantified using a Typhoon™ FLA 7000 phosphor-imager and expressed relative to lane 7 (i.e., the Pfr form of phyA in the absence of PIF3). (**c**) Comparison of AsphyA autophosphorylation with increasing concentrations of PIF3. 500 ng of AsphyA reacted with different concentrations of PIF3 (125, 250, and 500 ng) in the presence of 1.5 µg histone H1 for 30 min at room temperature. Intensities of AsphyA autophosphorylation (I_{rel}-phyA) and PIF3 phosphorylation by AsphyA (I_{rel}-PIF3) are expressed relative to the last lane (i.e., the Pfr form of AsphyA in the presence of 500 ng PIF3). Both histone H1 phosphorylation and histone H1-stimulated AsphyA autophosphorylation were reduced in the presence of PIF3 (a,b), and AsphyA autophosphorylation decreased as the concentration of PIF3 was increased (c).

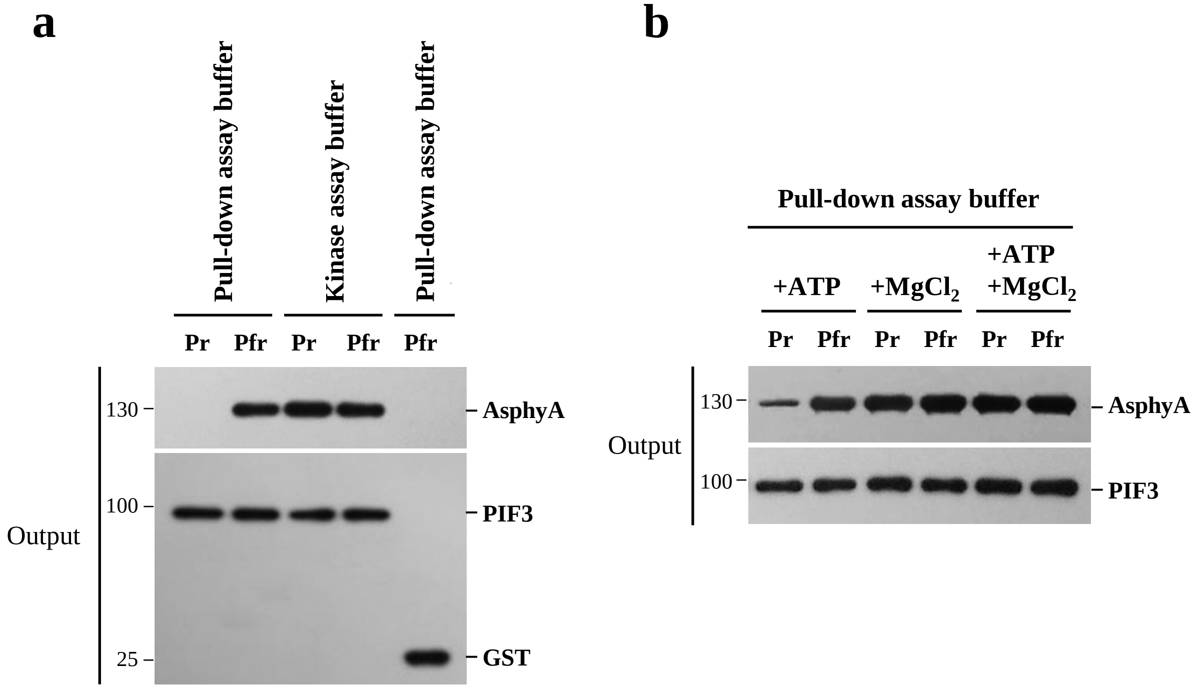

Supplementary Figure 4. Protein-protein interaction analysis between AsphyA and PIF3. (**a**) Interaction analysis of AsphyA with PIF3 in different buffer conditions. Full-length AsphyA protein was incubated with GST/strep-fused PIF3 in protein-protein interaction assay buffer (i.e., pull-down assay buffer; 100 mM Tris-HCl, pH 7.8, 1 mM EDTA, 150 mM NaCl, and 100 μg/ml BSA) or kinase assay buffer (25 mM Tris-HCl, pH 7.8, 0.2 mM EDTA, 5 mM $MgCl_2$, 4 mM DTT, and 150 μM ATP) at 4 °C for 90 min. Glutathione sepharose bead-bound proteins were then pelleted and analyzed by western blot analysis with AsphyA-specific (oat22) or GST-specific antibody. GST was used as a negative control. AsphyA interacted with PIF3 in a Pfr-specific manner in the pull-down buffer condition, whereas the Pr- and Pfr-indifferent interaction was observed in the kinase assay buffer. (**b**) Interaction analysis of AsphyA with PIF3 in the presence of ATP or $MgCl_2$. Pull-down assays were performed with component(s) present in the kinase assay buffer, 150 μM ATP and 5 mM $MgCl_2$. Except for the buffer conditions, the experiments were conducted under the same conditions in (a). Both Pr and Pfr forms of AsphyA interacted with PIF3 in the presence of $MgCl_2$.

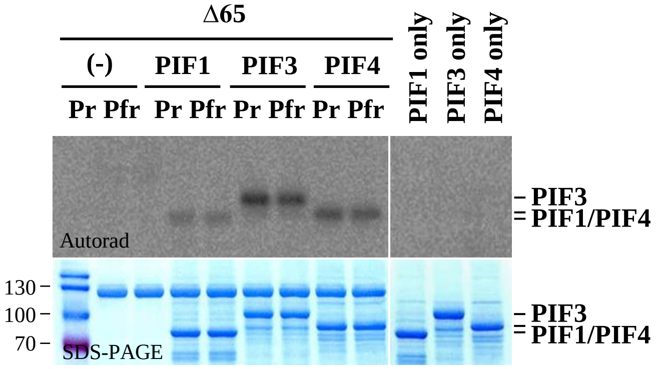

Supplementary Figure 5. Protein kinase activities of NTE-deleted AsphyA (Δ65) on PIFs. 1.0 μg of PIF1, PIF3, or PIF4 was added as substrates to reaction mixtures with 1.0 μg of Δ65 AsphyA. Samples with PIFs only were included as controls, in order to exclude the possibility that contaminating proteins were responsible for phosphorylating the PIFs.

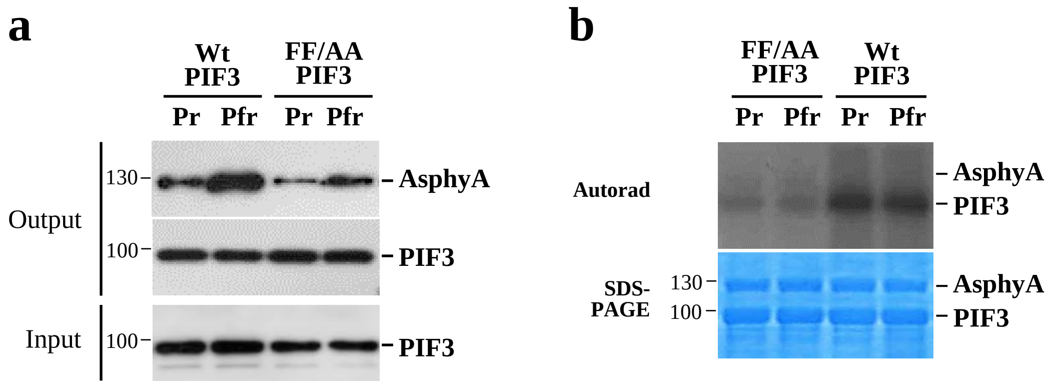

Supplementary Figure 6. Protein kinase activity of AsphyA on APA-mutant of PIF3. An active phyA-binding motif (APA) mutant of PIF3 was generated by site-directed mutagenesis of F203 and F209 to alanines (FF/AA). Compared with wild-type (Wt) PIF3, the interaction of FF/AA PIF3 with AsphyA was significantly reduced (**a**), and its phosphorylation by AsphyA was barely detectable (**b**).

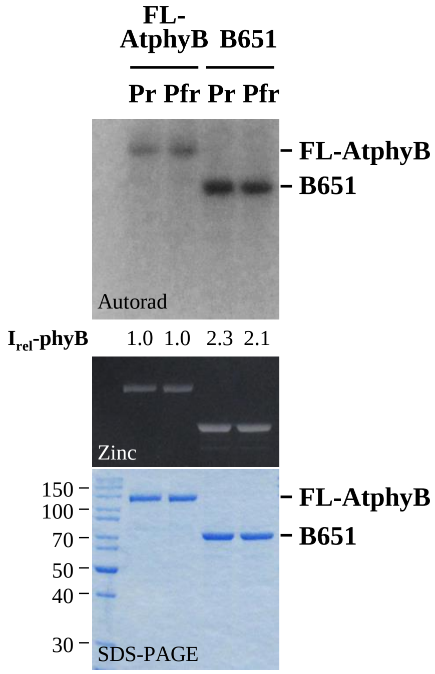

Supplementary Figure 7. Autophosphorylation of N-terminal domain of AtphyB. A reverse primer, 5′-GCAGCGCTTGCACCTAACTCATCAATCCCCT-3′ was used for the cloning of B651. Full-length (FL-AtphyB) and the N-terminal domain of AtphyB (1-651aa; B651) were expressed in *P. pastoris* and purified by streptavidin affinity chromatography. 1.0 μg of FL-AtphyB or B651 was used in the autophosphorylation analysis. Intensities of autophosphorylation are expressed relative to lane 1 (i.e., the Pr form of FL-AtphyB). These results indicate that the N-terminal domain of AtphyB possess stronger autophosphorylation activity than FL-AtphyB, which is consistent with the results obtained for AsphyA (i.e., A610).

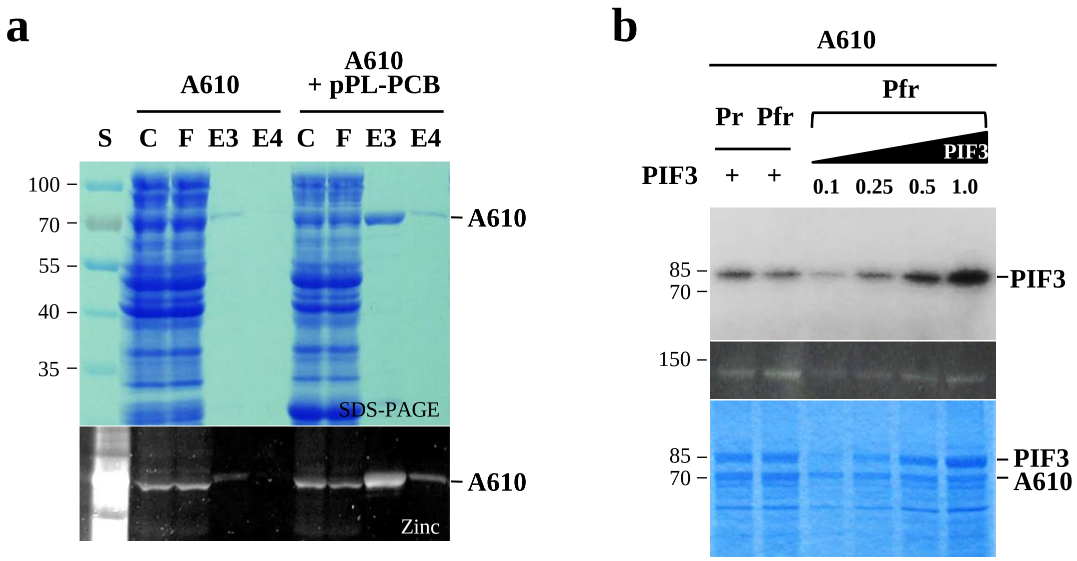

Supplementary Figure 8. PIF3 phosphorylation by A610 proteins purified from *E. coli*. (**a**) Expression and purification of recombinant A610 proteins from *E. coli*. The PCB-assembled A610 protein was expressed in *E. coli* that had been transformed with pBAD-A610/strep or *E. coli* that had been co-transformed with pBAD-A610/strep and pPL-PCB, and purified using streptavidin affinity chromatography. Lane S, PageRuler prestained protein ladder (Fermentas); lane C, crude extract (10 μL each); lane F, flow-through from the affinity column; lanes E3 & E4, elution fractions of purified holo-phyA (30 μL each). (**b**) PIF3 phosphorylation. 0.5 μg of A610, either Pr or Pfr (lanes 1 and 2), was used to investigate PIF3 phosphorylation. For concentration-dependent phosphorylation (lanes 3-6), 0.3 μg of A610 in Pfr form was allowed to react with increasing concentrations of PIF3 (0.1, 0.25, 0.5, and 1.0 μg) for 1.5 h at 30°C. Autoradiogram (Autorad), zinc florescence (Zinc) and SDS-PAGE gel are shown.

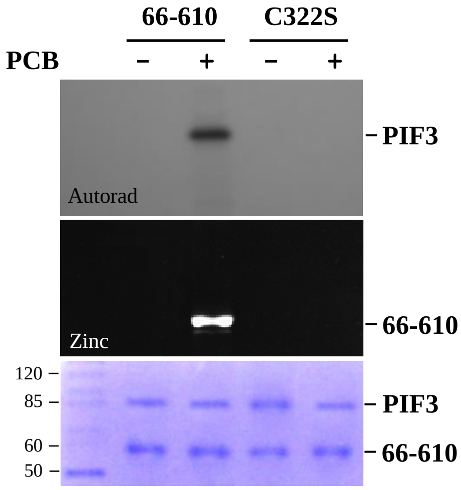

Supplementary Figure 9. PIF3 phosphorylation analysis by the photosensory core of C322S mutant. To exclude the possibility of protein kinase contamination from the PCB sample, a chromophore binding site mutant (Cys322 to Ser, C322S) of AsphyA was generated using site-directed mutagenesis with the following primers, 5'-GGCACCACACAGCAGCCACCTTCAGTATATGGAGAAC-3' (forward) and 5'-CATATACTGAAGGTGGCTGCTGTGTGGTGCCCTGAGTGC-3' (reverse). Then, the photosensory core proteins of wild-type and C322S mutant were purified with or without the addition of PCB, and used for PIF3 phosphorylation analysis. Our results showed no PIF3 phosphorylation by the C322S mutant, regardless of the PCB addition. Only PCB-assembled photosensory core of wild-type AsphyA phosphorylated PIF3. These results confirmed that no contaminating protein kinases were present in the PCB sample, and suggested that the chromophore-assembled photosensory core is required for PIF3 phosphorylation.

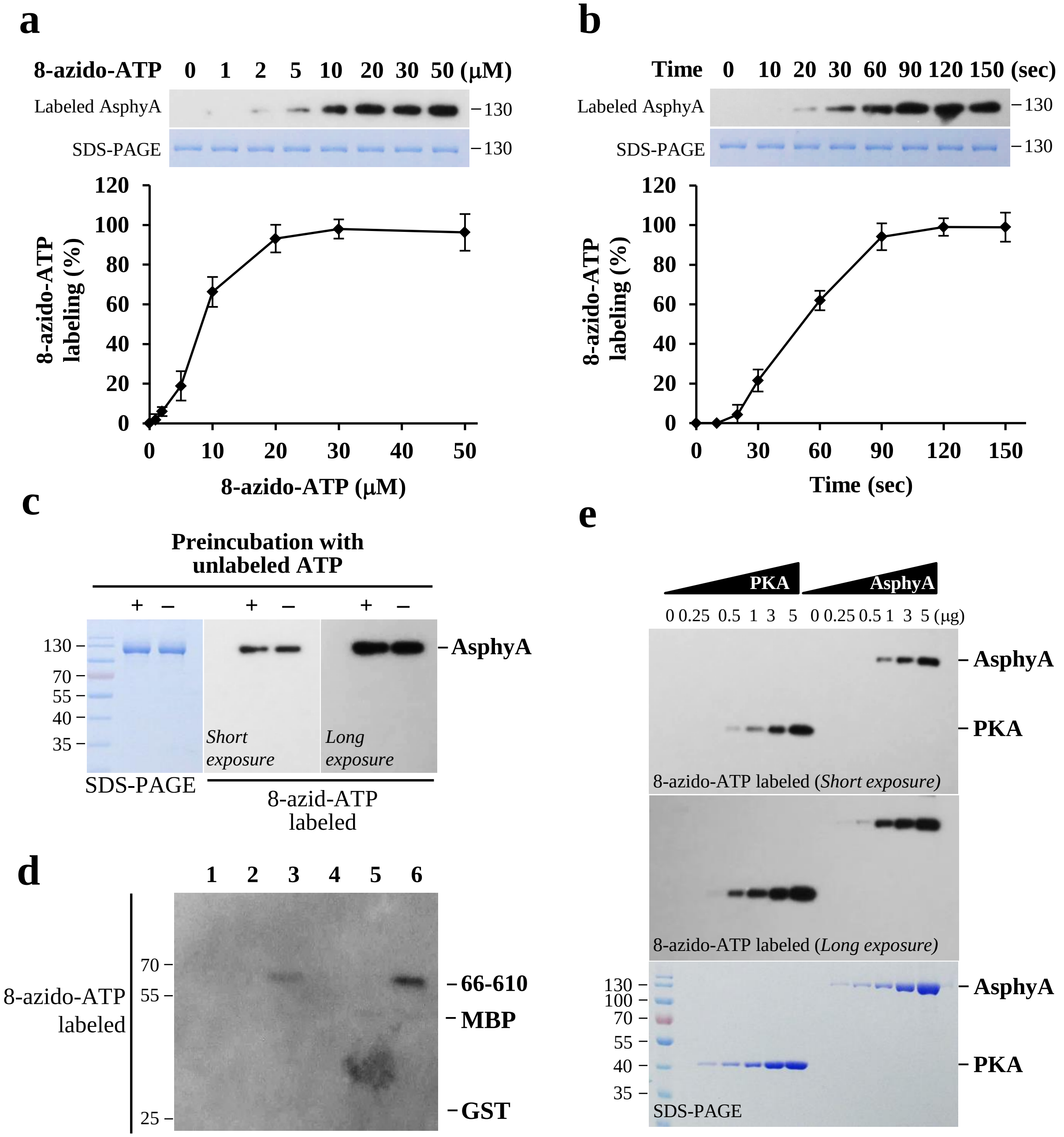
a
8-azido-ATP 0 1 2 5 10 20 30 50 (μM)
Labeled AsphyA
SDS-PAGE
130
8-azido-ATP labeling (%)
8-azido-ATP (μM)
b
Time 0 10 20 30 60 90 120 150 (sec)
Labeled AsphyA
SDS-PAGE
130
8-azido-ATP labeling (%)
Time (sec)
c
Preincubation with unlabeled ATP
+ –
AsphyA
Short exposure
Long exposure
SDS-PAGE
8-azid-ATP labeled
d
1 2 3 4 5 6
8-azido-ATP labeled
66-610
MBP
GST
e
PKA
AsphyA
0 0.25 0.5 1 3 5 0 0.25 0.5 1 3 5 (μg)
AsphyA
PKA
8-azido-ATP labeled (Short exposure)
8-azido-ATP labeled (Long exposure)
SDS-PAGE

Supplementary Figure 10. Photoaffinity labeling of AsphyA with 8-N_3-ATP-biotin-long chain-hydrazone (8-azido-ATP). (**a**) The concentration-dependent photoaffinity labeling of AsphyA with 8-azido-ATP. 1.0 μg of purified FL-AsphyA (Pfr form) was pre-incubated in a photoaffinity labeling buffer containing unlabeled-ATP for 30 min on ice, and 8-azido-ATP was applied at the indicated concentrations. After incubation for an additional 5 min on ice, UV light at 254 nm was irradiated for 90 s. 8-azido-ATP labeled proteins were then detected using an ECL western blotting analysis system (Thermo Scientific) with avidin-HRP. The Coomassie-stained SDS-PAGE gel shows the loading controls. (**b**) UV irradiation time-dependent photoaffinity labeling of AsphyA. 10 μM 8-azido-ATP was applied to reaction mixtures with 1.0 μg of FL-AsphyA (Pfr form), which were then irradiated with UV light for the indicated times. Error bars in (a) and (b) represent s.d. from three measurements. (**c**) Effect of preincubation with unlabeled-ATP on photoaffinity labeling. To examine the possible existence of contaminating protein kinase(s) in the AsphyA samples prepared from *P. pastoris*, the 8-azido-ATP labeling was performed without the unlabeled-ATP preincubation step, and azido-ATP labeled proteins were detected with long exposure (*ca.* 45 s) in addition to the normal short exposure condition (*ca.* 15 s). In our experimental conditions, no other 8-azido-ATP-labeled protein band except AsphyA was detected in the applied conditions, even in the conditions such as no unlabeled-ATP preincubation and long exposure time. (**d**) 8-azido-ATP labeling of AsphyA in the presence of negative control proteins. The photosensory core of AsphyA (66-610) was used for this assay. 1.0 μg of 66-610 AsphyA was allowed to react with 10 μM 8-azido-ATP in the presence of 2.0 μg of GST or MBP (maltose-binding protein). As controls, 8-azido-ATP labeling of GST or MBP only was also included. Lane 1, GST only; lane 2, GST reacted with 10 μM 8-azido-ATP; lane 3, 66-610 AsphyA reacted with 10 μM 8-azido-ATP in the presence of 2.0 μg GST; lane 4, MBP only; lane 5, MBP reacted with 10 μM 8-azido-ATP; lane 6, 66-610 AsphyA reacted with 10 μM 8-azido-ATP in the presence of 2.0 μg MBP. While GST and MBP were not labeled with 8-azido-ATP, AsphyA was labeled even in the presence of other proteins such as GST and MBP. (**e**) Comparison of 8-azido-ATP labeling of AsphyA with a known protein kinase. PKA (protein kinase A purchased from NEB) was included in the 8-azido-ATP photoaffinity labeling reactions as a positive control. Increasing amounts of PKA and AsphyA (0, 0.25, 0.5, 1.0, 3.0, and 5.0 μg) were used for the photoaffinity labeling reactions with 10 μM 8-azido-ATP and UV irradiation of 90 s. In our experimental conditions, PKA was specifically labeled with 8-azido-ATP.

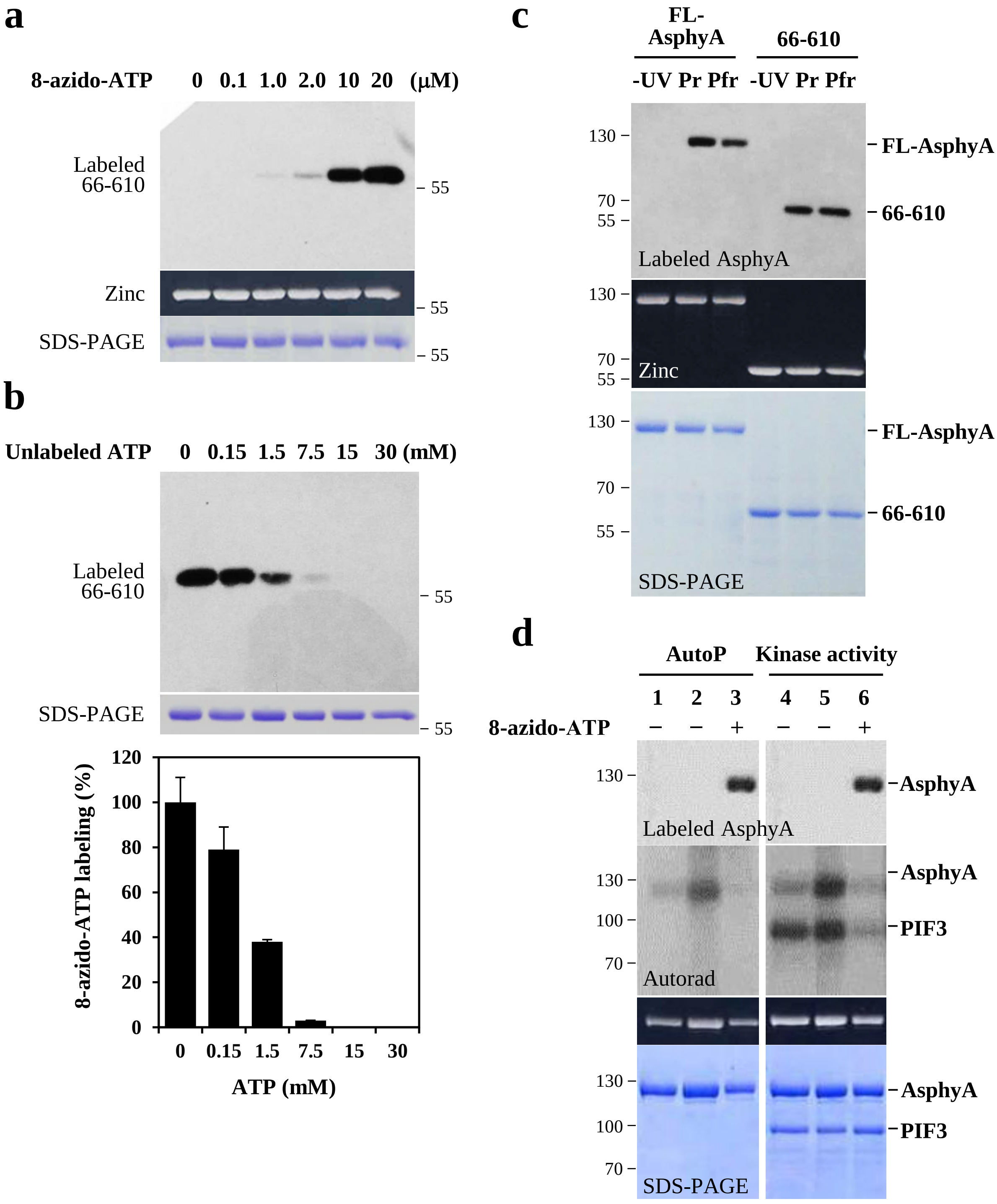

a
8-azido-ATP 0 0.1 1.0 2.0 10 20 (μM)
Labeled 66-610
Zinc
SDS-PAGE
55
b
Unlabeled ATP 0 0.15 1.5 7.5 15 30 (mM)
Labeled 66-610
SDS-PAGE
55
8-azido-ATP labeling (%)
0 20 40 60 80 100 120
0 0.15 1.5 7.5 15 30
ATP (mM)
c
FL-AsphyA
66-610
-UV Pr Pfr -UV Pr Pfr
130
70
55
FL-AsphyA
66-610
Labeled AsphyA
Zinc
SDS-PAGE
d
AutoP
Kinase activity
1 2 3 4 5 6
8-azido-ATP − − + − − +
AsphyA
Labeled AsphyA
PIF3
100
Autorad
SDS-PAGE

Supplementary Figure 11. Photoaffinity labeling of the photosensory core and the effect of azido-ATP labeling on AsphyA kinase activity. (**a**) 8-azido-ATP labeling of the photosensory core (i.e., 66-610). 1.0 μg of 66-610 protein (Pfr form) was allowed to react with the indicated concentrations of 8-azido-ATP and UV irradiation for 90 s. 8-azido-ATP labeled proteins were then detected using avidin-HRP. (**b**) Specificity analysis of photoaffinity labeling of the photosensory core. The reaction of the photosensory core with 10 μM 8-azido-ATP was carried out using increasing amounts of unlabeled ATP (0-30 mM). The band intensities were quantified using ImageJ software, and the percentages of 8-azido-ATP labeling were normalized to the 66-610 protein signals. Error bars represent s.d. from three measurements. 8-azido-ATP photoaffinity labeling on 66-610 was inhibited by the treatment of approximately 7.5 mM unlabeled ATP. (**c**) Photoaffinity labeling of the Pr and Pfr forms with 8-azido-ATP. 1.0 μg of FL- or 66-610 AsphyA was photoaffinity-labeled with 10 μM 8-azido-ATP. Reaction without UV illumination (-UV) was shown as a negative control. (**d**) Autophosphorylation and kinase activity assays of 8-azido-ATP-labeled AsphyA. The Pfr forms of FL-AsphyA were used for these assays. Lanes 1 and 4, phosphorylation of AsphyA in the kinase assay buffer; lanes 2 and 5, phosphorylation of AsphyA in the 8-azido-ATP labeling buffer; lanes 3 and 6, phosphorylation of 8-azido-ATP-labeled AsphyA in the 8-azido-ATP labeling buffer.

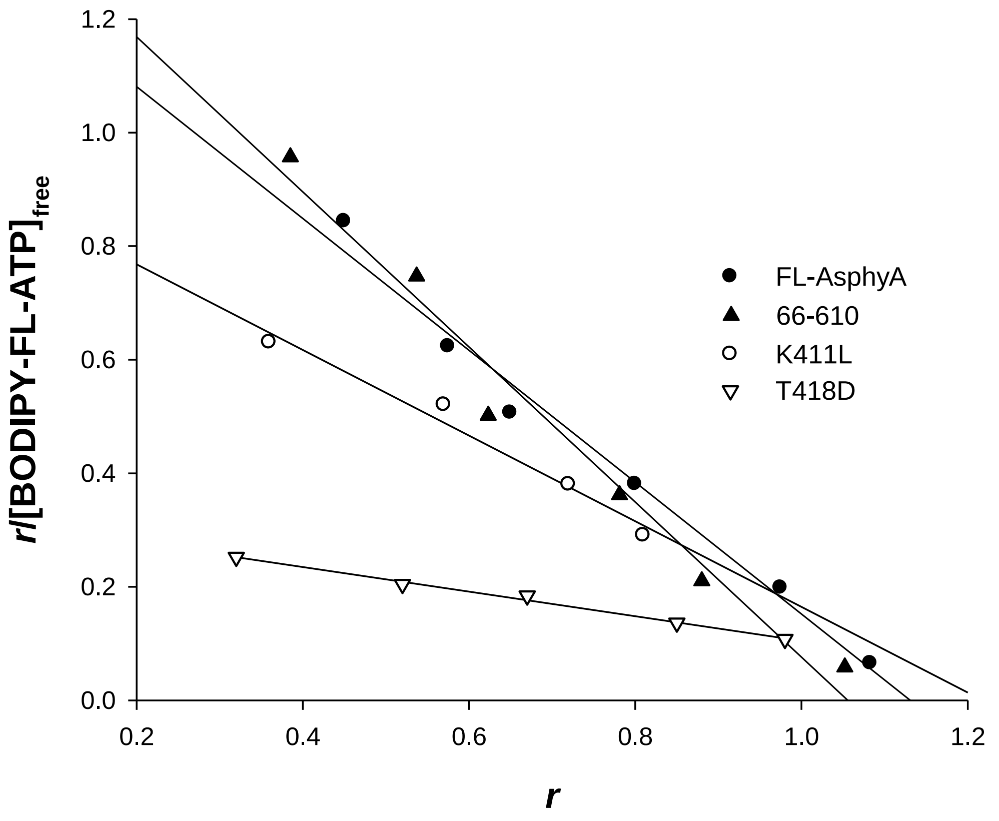

Supplementary Figure 12. Determination of binding parameters of ATP in AsphyA proteins. The number of binding sites (*n*) and dissociation constant (K_d) of ATP in AsphyA proteins were determined by Scatchard plots using an ATP analogue, BODIPY-FL-ATP (BODIPY® FL 2′-(or-3′)-O-(*N*-(2-aminoethyl)urethane) adenosine triphosphate). The *n* and K_d values were calculated from the slopes ($-1/K_d$) and x-axis intercepts (*n*) of the Scatchard plot of r/[BODIPY-FL-ATP]$_{free}$ versus *r*, where *r* was the molar ratio of [BODIPY-FL-ATP]$_{bound}$ to [AsphyA]$_{total}$. The *n* values of full-length (FL) AsphyA and photosensory core (66-610) were both calculated at around one per polypeptide, and the K_d values were calculated at approximately 0.87×10^{-6} M for FL-AsphyA, 0.72×10^{-6} M for 66-610, 1.32×10^{-6} M and 4.76×10^{-6} M for the K411L and T418D mutants, respectively. In the case of the D422R mutant, we were unable to obtain the K_d value at micromolar level, possibly due to too low ATP binding.

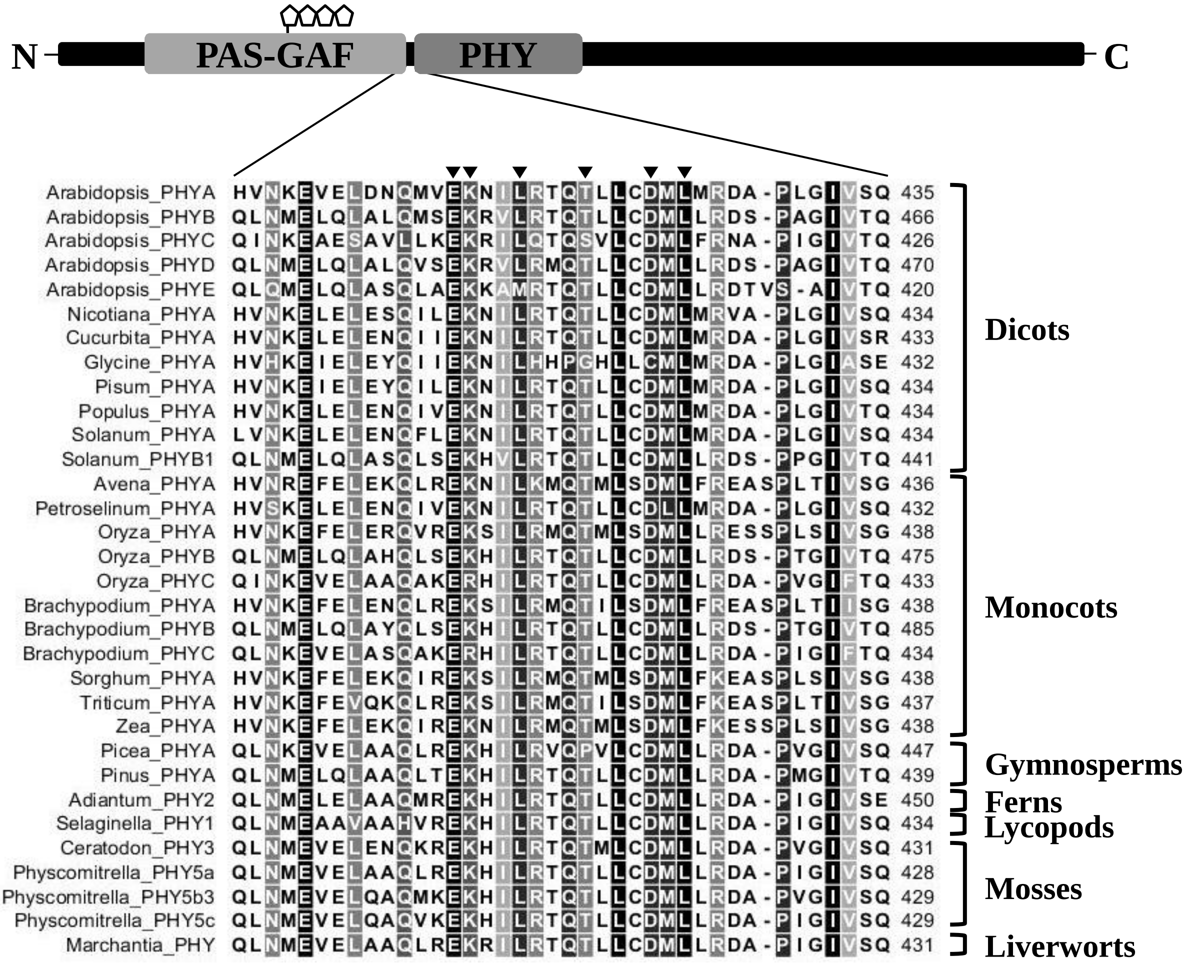

Supplementary Figure 13. Amino acid sequence alignment of the junction region of GAF and PHY domain. The sequences of various phytochromes from diverse taxa were used for the alignment of the junction sequences of the GAF (217-401aa) and PHY (413-593aa) domains of AsphyA. Black and gray boxes denote identical and similar residues, respectively. Filled arrowheads (E410, K411, L414, T418, D422, and L424) indicate the sites for mutagenesis that were targeted during mutagenesis to obtain mutants with altered kinase activities. The sequences were aligned using CLC Main Workbench.

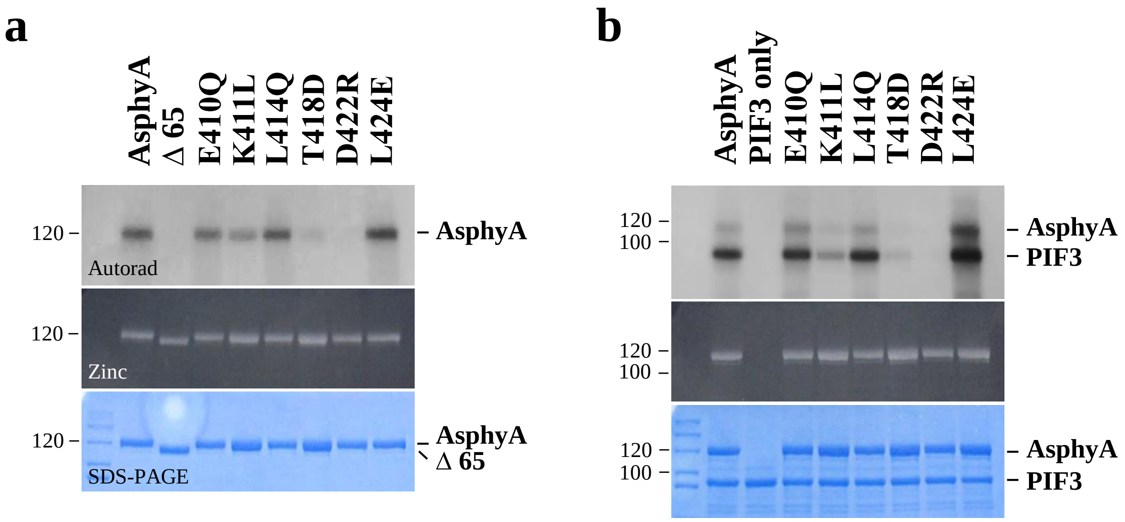

Supplementary Figure 14. Phosphorylation assays of representative AsphyA mutants. (**a**) Autophosphorylation assays of the site-specific AsphyA mutants. Since the autophosphorylation sites of AsphyA are located in the N-terminal extension (NTE), the NTE-deleted AsphyA (Δ65) was included as a negative control. (**b**) Protein kinase activity assays of the site-specific mutants. PIF3 was used as the substrates, and PIF3 only (i.e., without AsphyA) was included as a negative control. All the phosphorylation reactions were performed in the absence of histone H1.

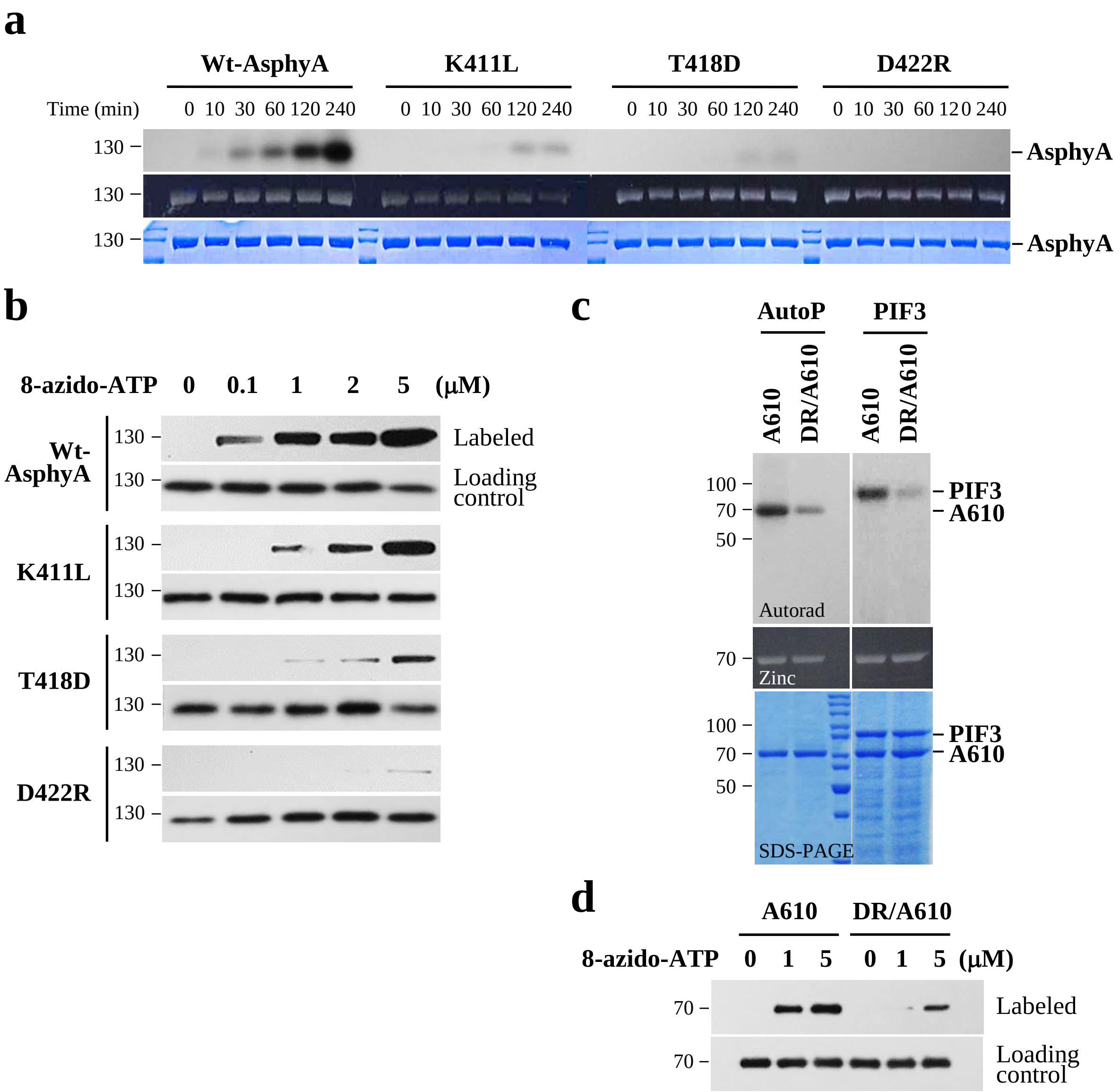
a
Wt-AsphyA
K411L
T418D
D422R
Time (min)
0 10 30 60 120 240
0 10 30 60 120 240
0 10 30 60 120 240
0 10 30 60 120 240
130
130
130
AsphyA
AsphyA
b
8-azido-ATP 0 0.1 1 2 5 (μM)
Wt-AsphyA
K411L
T418D
D422R
130
Labeled
Loading control
c
AutoP
PIF3
A610
DR/A610
A610
DR/A610
100
70
50
Autorad
PIF3
A610
70
Zinc
100
70
50
SDS-PAGE
PIF3
A610
d
A610
DR/A610
8-azido-ATP 0 1 5 0 1 5 (μM)
70
70
Labeled
Loading control

Supplementary Figure 15. Phosphorylation and photoaffinity labeling analysis of AsphyA mutants with reduced kinase activity. (**a**) Autophosphorylation assays of the kinase mutants. 1.0 μg of purified full-length wild-type (Wt) and mutant (K411L, T418D and D422R) AsphyA proteins were used. (**b**) ATP-binding affinity analysis of AsphyA kinase mutants by photoaffinity labeling with 8-azido-ATP. Full-length wild-type and mutant AsphyA proteins were labeled with the indicated concentrations of 8-azido-ATP, and the azido-ATP-labeled proteins were detected with avidin-HRP (upper panels). Amounts of AsphyA proteins (*ca.* 1.0 μg) used in these reactions are shown as loading controls in lower panels, which were analyzed using western blots with oat22 antibody. These results were quantitatively analyzed using ImageJ and shown in Fig. 4c. (**c**) Phosphorylation analysis of the D422R mutant of A610 (DR/A610). Recombinant DR/A610 proteins were prepared using the *Pichia* protein expression system and streptavidin affinity chromatography. 1.0 μg of purified wild-type A610 and DR/A610 mutant proteins were used for autophosphorylation analysis (left panel), and 1.0 μg of GST/strep-fused PIF3 protein was used for the kinase activity analysis (right panel). (**d**) ATP-binding affinity analysis of DR/A610. Wild-type (A610) and DR/A610 mutant proteins were labeled with the indicated concentrations of 8-azido-ATP, and the 8-azido-ATP labeled proteins were detected with avidin-HRP (upper panel).

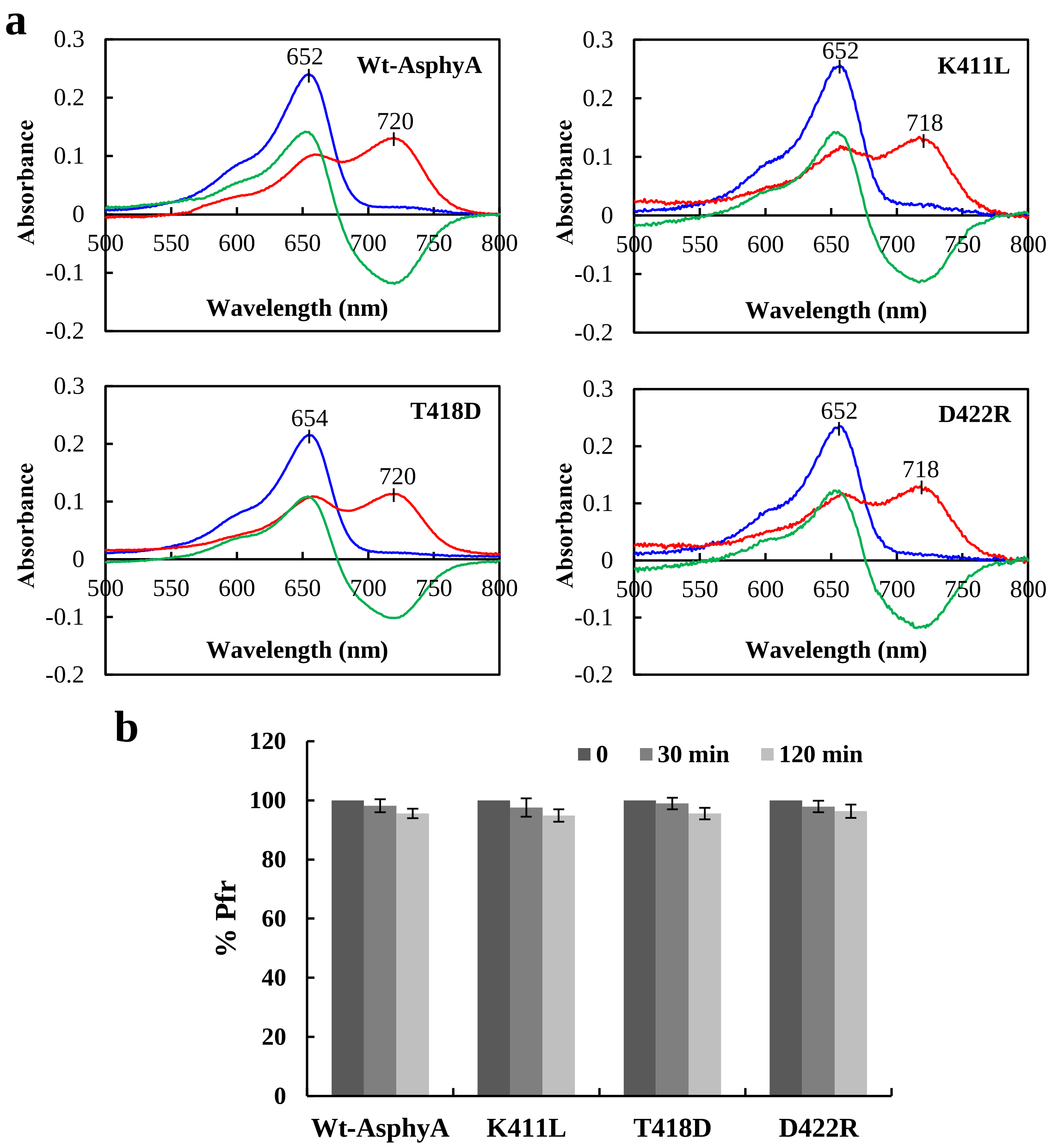

Supplementary Figure 16. Photochemical analyses of AsphyA kinase mutants. (**a**) Analysis of absorption and difference spectra. Pr (blue)/Pfr (red) absorption spectra and difference spectra (green) of purified FL-AsphyA mutants are shown. The three mutants exhibited similar absorption wavelength maxima of Pr and Pfr (λ_{pr} and λ_{pfr}), and the ratios of the heights of Pr and Pfr peaks (A_{max}/A_{min}) were also similar to that of wild-type (Wt) AsphyA. (**b**) Analysis of dark reversion. AsphyA proteins were phototransformed to Pfr forms by irradiation with red light for 15 min, and the amounts of Pfr were then estimated during 120 min in the dark. Error bars represent s.d. from three measurements. There was no significant difference in dark reversions of wild-type and the kinase mutants.

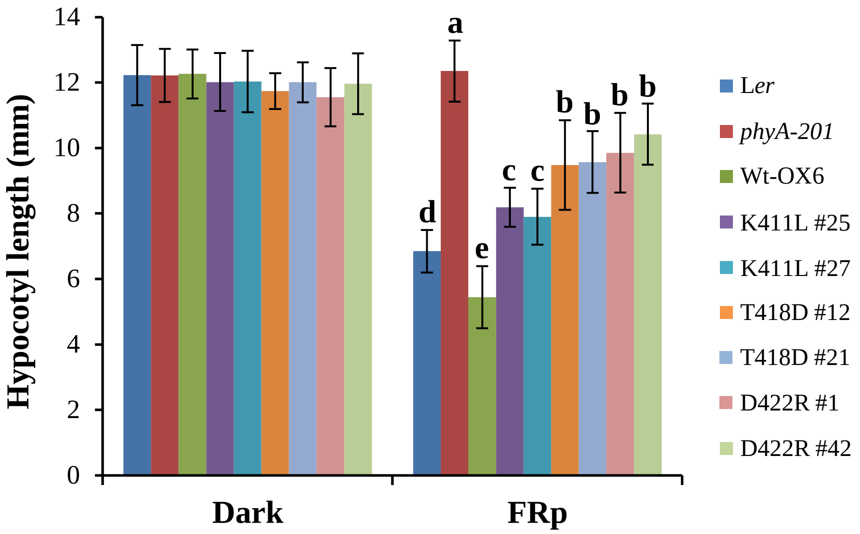

Supplementary Figure 17. Hypocotyl de-etiolation of transgenic *phyA-201* plants overexpressing AsphyA kinase mutants with pulsed FR. To investigate a far-red very low fluence response (FR-VLFR), 1-d-old dark-grown seedlings were grown with hourly 5-min pulses of FR (0.5 μmol·m^{-2}·s^{-1}) or in the dark for 3 d. Error bars represent standard deviations (n = 26). Means with different letters are significantly different at $P < 0.001$, using Tukey's test.

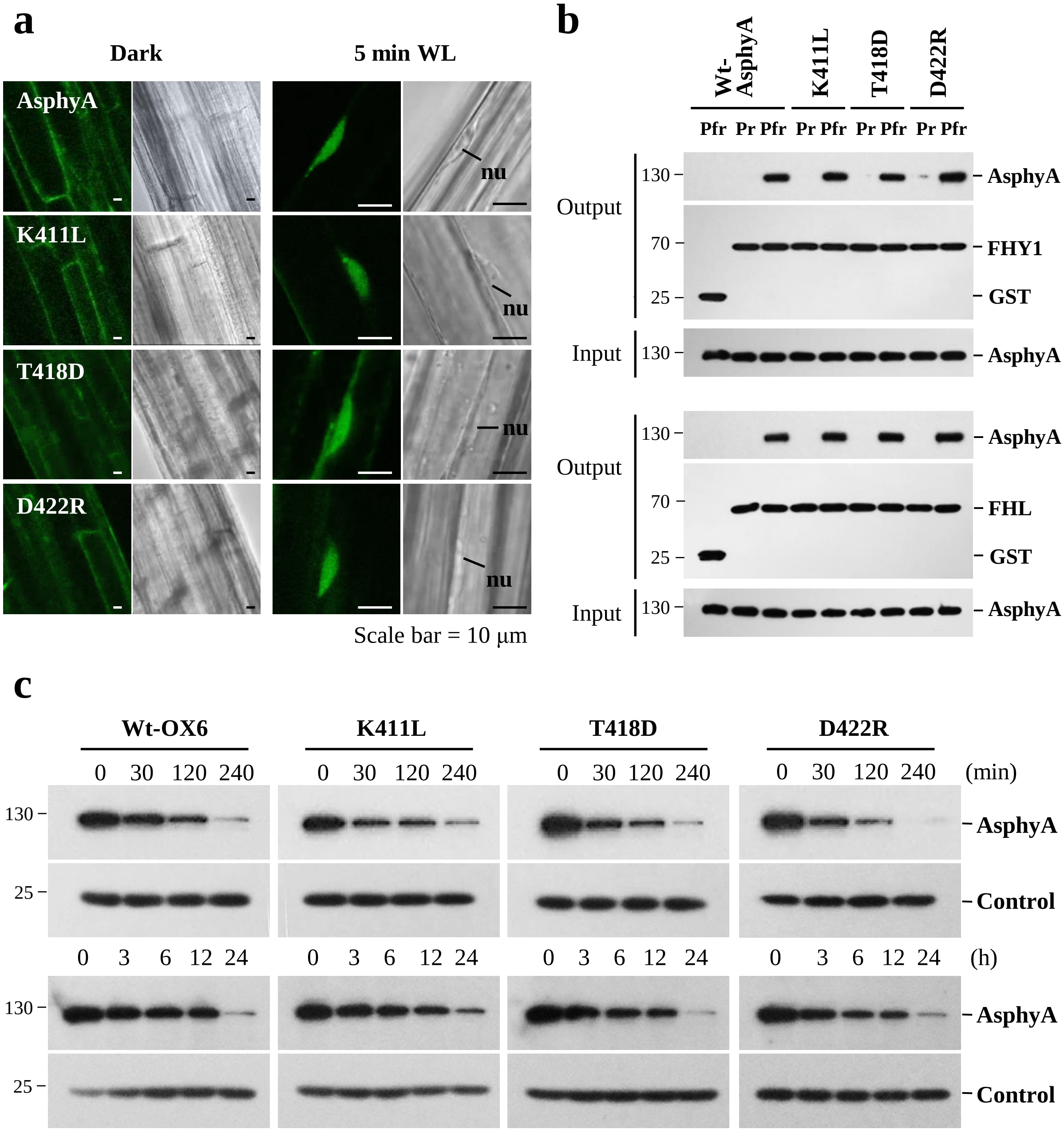
a
Dark
5 min WL
AsphyA
K411L
T418D
D422R
nu
Scale bar = 10 μm
b
Wt-AsphyA
K411L
T418D
D422R
Pfr
Pr
Output
Input
130
70
25
AsphyA
FHY1
GST
FHL
c
Wt-OX6
0 30 120 240 (min)
0 3 6 12 24 (h)
Control

Supplementary Figure 18. Light-induced nuclear localization and protein degradation of AsphyA kinase mutants. (**a**) Light-induced nuclear localization of AsphyA kinase mutants. Transgenic *phyA-201* plants with eGFP-fused K411L, T418D, or D422R mutant were generated and used for this analysis. 4-d-old dark-grown seedlings were used directly (Dark) or subjected to white light treatment (5 min WL) prior to confocal microscopic analysis using a Laser Scanning Confocal Microscope (Leica TCS SP5 AOBS/Tandem). GFP fluorescence was clearly detectable in hypocotyl nucleus (nu) of all seedlings under white light. (**b**) Protein-protein interaction of AsphyA kinase mutants with FHY1 and FHL. Full-length wild-type and three kinase mutant AsphyA proteins were incubated with GST/strep-fused FHY1 (top) or FHL (bottom) at 4°C, and glutathione sepharose bead-bound proteins were pelleted and analyzed using western blots with oat22 or GST-specific antibody. GST was included as a negative control. All of the kinase mutants, as well as the wild-type AsphyA, interacted with both FHY1 and FHL in a Pfr-specific manner. (**c**) Light-induced degradation of AsphyA proteins. 4-d-old dark-grown seedlings of each transgenic plant were exposed to continuous white light (top; 150 $\mu mol \cdot m^{-2} \cdot s^{-1}$) or far-red light (bottom; 5 $\mu mol \cdot m^{-2} \cdot s^{-1}$) for the indicated periods. 40 μg of total protein extracts was used for western blot analysis to detect AsphyA with oat22 antibody. Loading controls using polyclonal antibody against *Arabidopsis* translationally controlled tumor protein (AtTCTP; At3g16640) are shown in the lower panels.

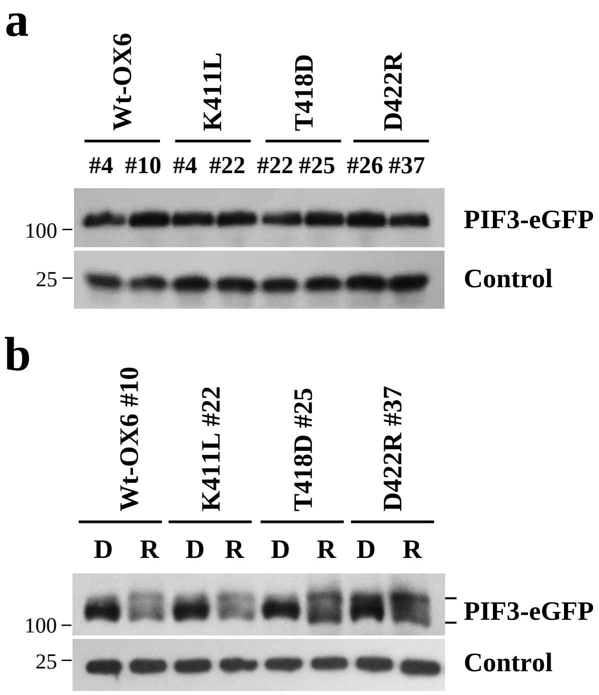

Supplementary Figure 19. Red light-induced phosphorylation of PIF3 in transgenic plants co-expressing PIF3-eGFP and AsphyA kinase mutants. (**a**) Levels of PIF3 proteins in the transgenic *phyA-201* plants co-expressing AsphyA kinase mutants and eGFP-PIF3. Protein extracts from 5-d-old dark-grown seedlings were probed with anti-GFP antibody. Loading control as shown in the lower panel. (**b**) R-induced phosphorylation of PIF3. 4-d-old dark-grown seedlings were exposed to R light (10 $\mu mol \cdot m^{-2} \cdot s^{-1}$) for 20 min before harvesting for protein extraction.

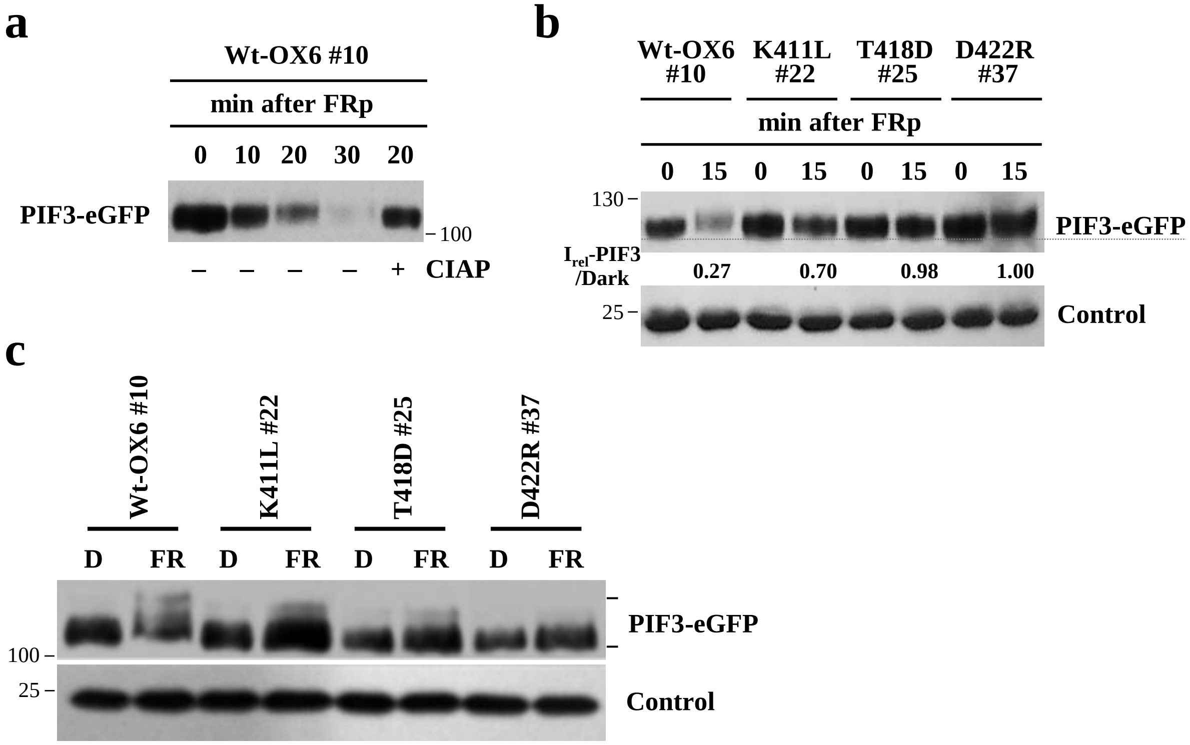

Supplementary Figure 20. FR-induced mobility shift analyses of PIF3 with MG132 treatment. (**a**) Phosphatase treatment of PIF3 protein samples extracted from FRp-exposed seedlings. 4-d-old dark-grown seedlings of the transgenic PIF3/Wt-OX6 plants were pretreated with 50 μM MG132 for 4 h, then exposed to FRp (25 $\mu mol \cdot m^{-2} \cdot s^{-1}$ for 5 min; 7500 $\mu mol \cdot m^{-2}$), and incubated in the dark for the time indicated before harvesting for protein extraction. For phosphatase treatment, calf intestinal alkaline phosphatase (CIAP) was added to the PIF3 protein sample that had been prepared from FRp-exposed seedling extracts by immunoprecipitation with anti-GFP antibody. The mobility-shifted bands of PIF3 could be eliminated in the presence of CIAP. (**b**) PIF3 phosphorylation and degradation in FRp-exposed seedlings with MG132 pretreatment. 4-d-old dark-grown seedlings were pretreated with 50 μM MG132 for 4 h, then exposed to FRp (7500 $\mu mol \cdot m^{-2}$), and incubated in the dark for 15 min before harvesting for protein extraction. Intensities of PIF3 proteins are expressed relative to the PIF3 protein levels of each dark-grown seedling sample (i.e., extracts at 0 min after FRp). A line is included to show the bottom of the PIF3 protein bands. (**c**) Mobility shift analysis of PIF3 with the treatment of higher MG132 concentration for longer time. 4-d-old dark-grown seedlings were treated with 80 μM MG132 in half strength MS liquid medium for 12 h, then exposed to FRp (7500 $\mu mol \cdot m^{-2}$), and incubated under dark for 10 min in the same liquid medium with 80 μM MG132 before harvesting for protein extraction. The extracted proteins were separated on 6.5% SDS-PAGE gels for western blot analysis. Even in the different conditions of MG132 treatment, we observed consistently little mobility shift and degradation of PIF3 proteins in both PIF3/T418D and PIF3/D422R plants, whereas higher molecular weight PIF3 was clearly detected in the PIF3/Wt-OX6 plant. In the case of the PIF3/K411L plant, a mobility shift of PIF3 was observed, but it was significantly reduced compared with that of the PIF3/Wt-OX6 plant.

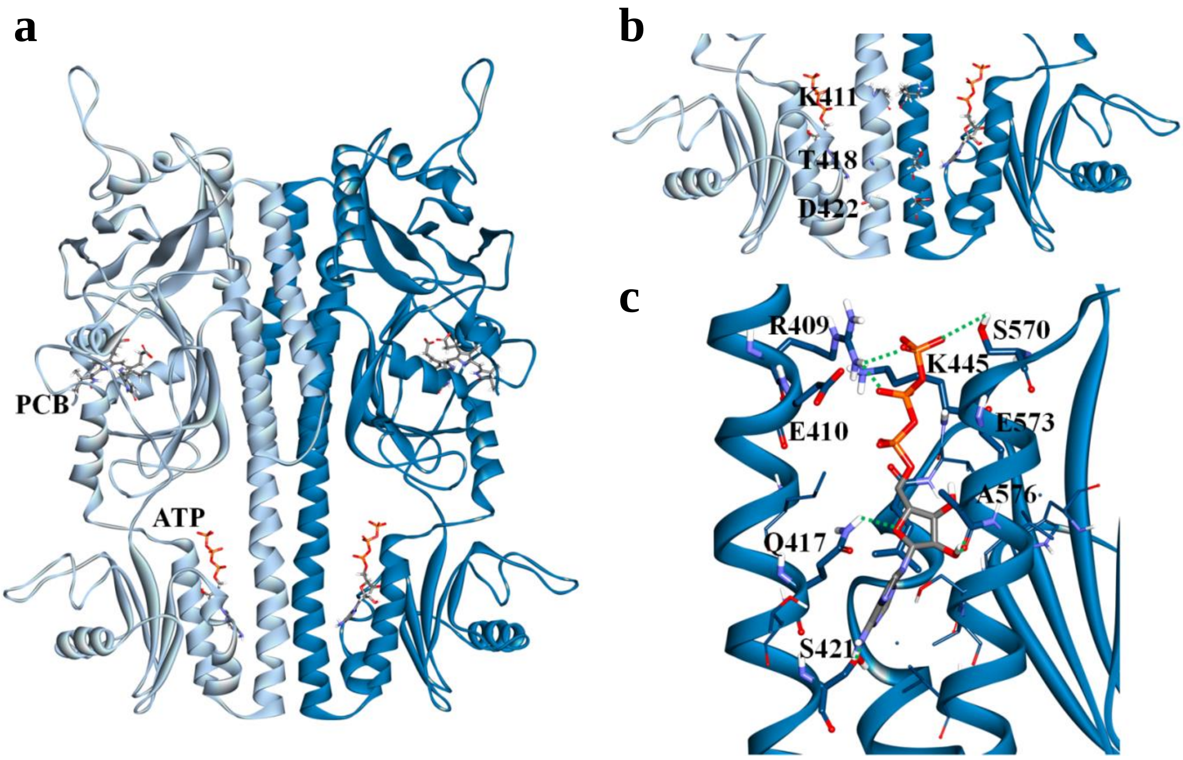

Supplementary Figure 21. Predicted ATP binding to a homology-modeled structure of the photosensory core of AsphyA. (**a**) Predicted three-dimensional structure of the Pfr form of the AsphyA photosensory core (V66 to T587), as a dimer. Subunit A (light blue) and subunit B (blue) are shown as cartoon models. ATP and phycocyanobilin (PCB) molecules are labeled and represented as stick models. The ATP molecule was simulated to bind to a putative binding pocket in PHY domain. (**b**) Mutation positions used to generate the kinase mutants of AsphyA. The residues for the kinase mutants are labeled and represented as sticks. K411, T418, and D422 are located in the interface between α9 helices. (**c**) The detailed binding mode of ATP in wild-type AsphyA. Hydrogen bonding residues are labeled and represented as stick models and hydrogen bonds are represented as dotted green lines. Hydrophobic interacting residues are shown as line models (see also Supplementary Table 1).

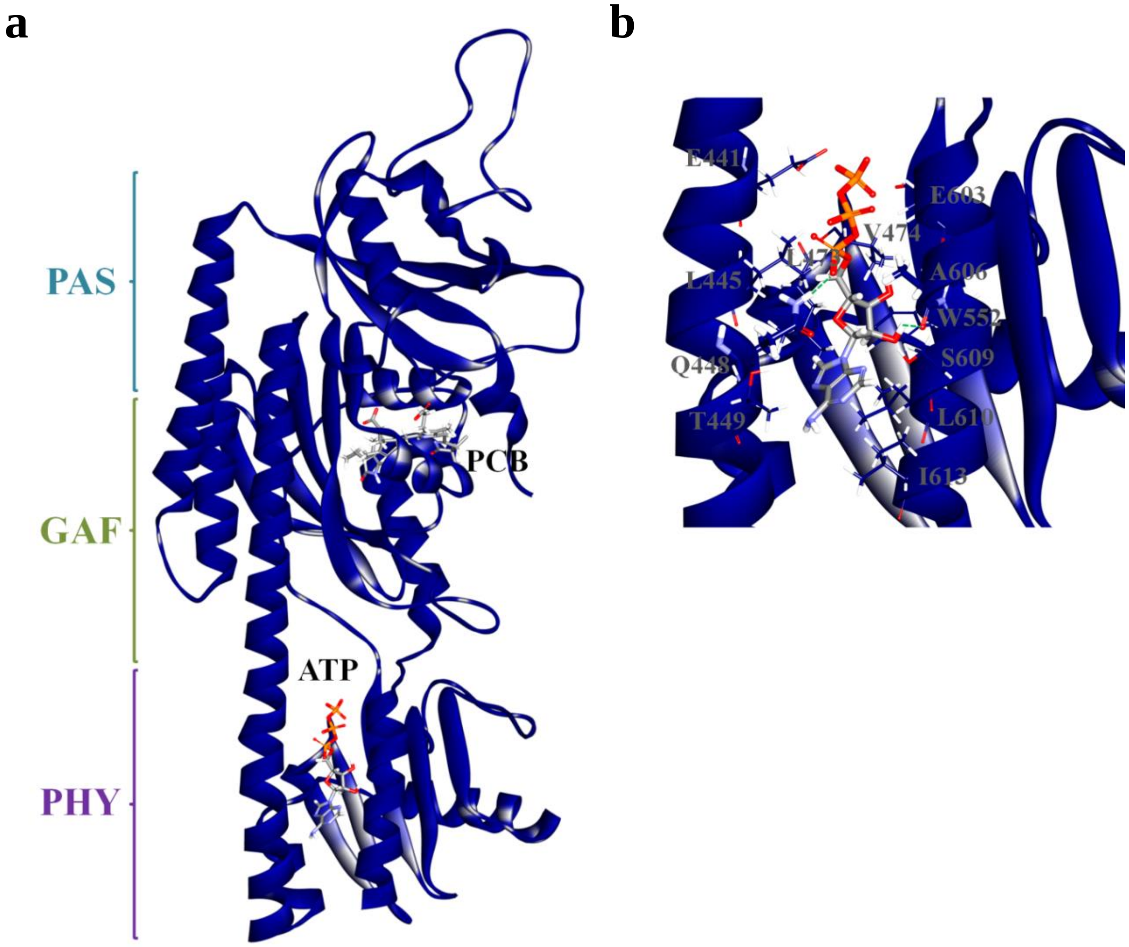

Supplementary Figure 22. Prediction of an ATP-binding site in the photosensory core of AtphyB. (**a**) The ATP-bound AtphyB structure built by homology modeling and molecular docking simulation. Since the AtphyB template (PDB code: 4OUR) includes missing parts in the structure of the PHY domain, we built the structure for the missing parts using MODELLER, and the ATP-binding mode in AtphyB was then predicted with energy minimization. The modeled photosensory core structure of AtphyB is shown as a cartoon model. ATP and PCB molecules are labeled and represented as stick models. Overall, the backbone structure of AtphyB is almost identical to that of AsphyA, and the ATP molecule was predicted to bind in the PHY domain. (**b**) ATP binding mode in the photosensory core of AtphyB. Conserved amino acid residues that interact with ATP are labeled and represented as stick models, and hydrogen bonds are represented as dotted green lines. It is notable that the R group of Q448 (which is corresponding to Gln417 of AsphyA) is involved in the hydrogen bonding with ATP.

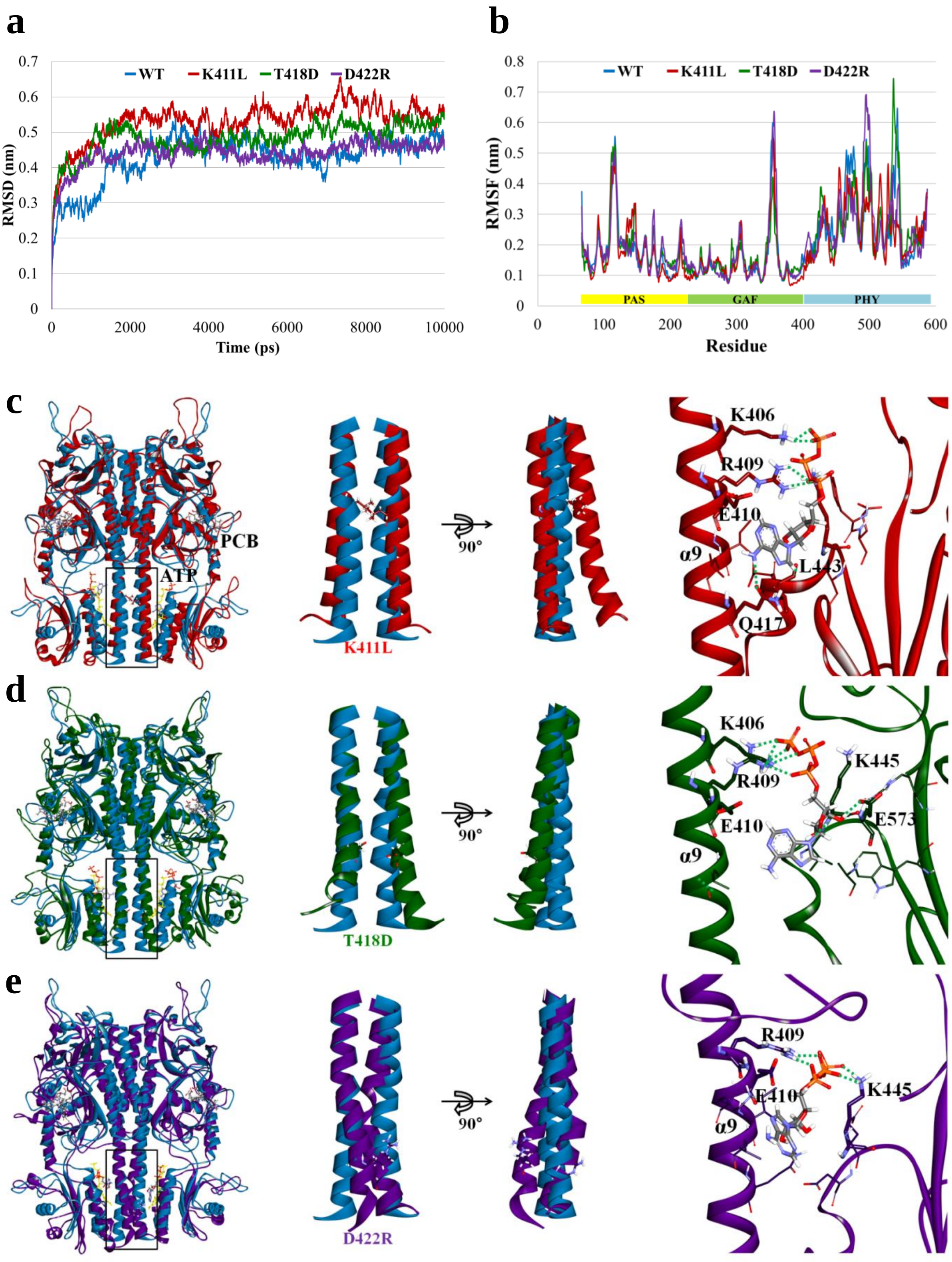
a
b
WT
K411L
T418D
D422R
RMSD (nm)
Time (ps)
RMSF (nm)
Residue
PAS
GAF
PHY
c
d
e
PCB
ATP
90°
K411L
T418D
D422R
K406
R409
E410
α9
L443
Q417
K445
E573

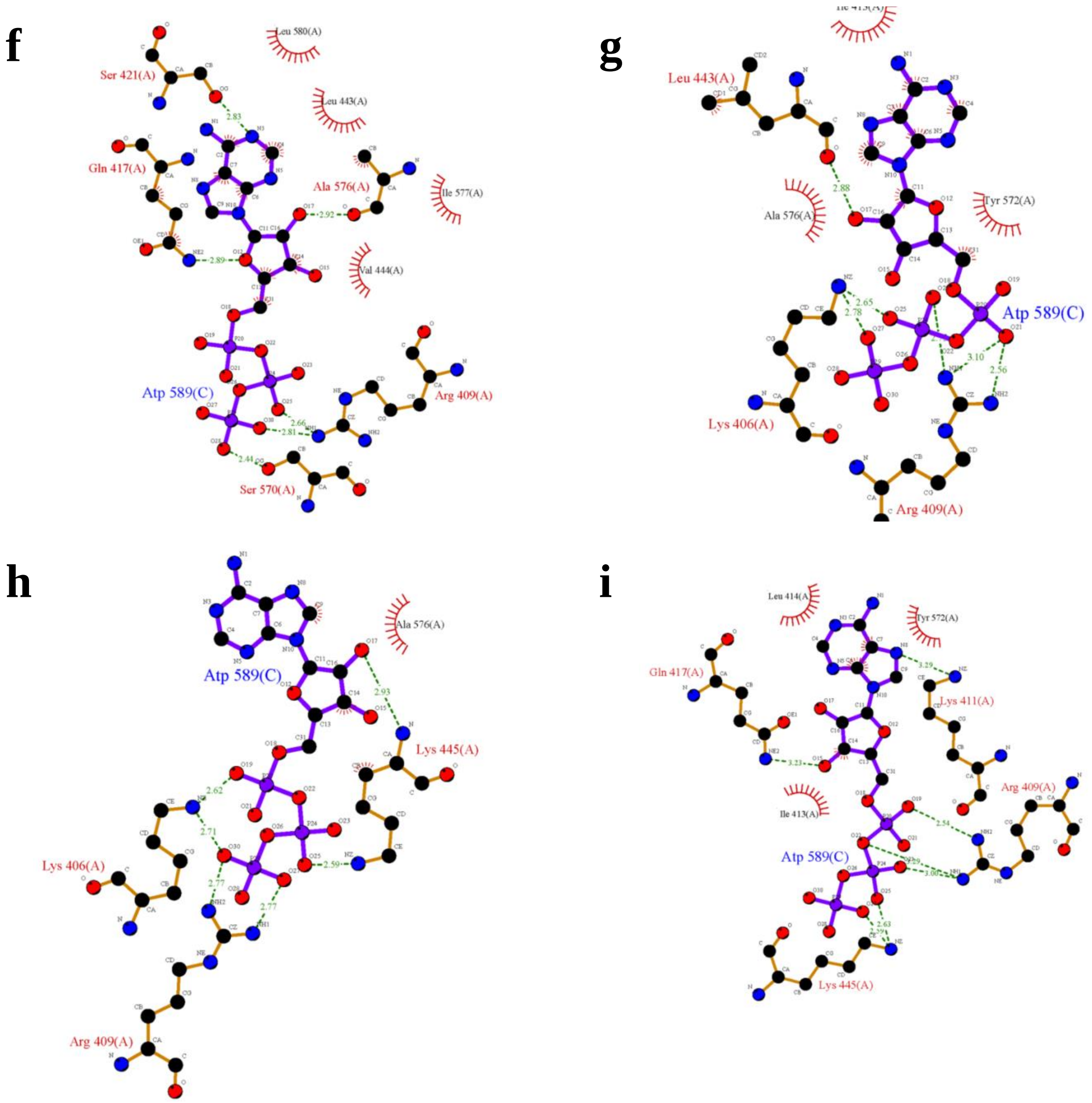

Supplementary Figure 23. Structural comparisons between wild-type and the kinase mutants of AsphyA. (**a**) Root mean square deviation (RMSD) plots of the backbone atoms of the WT and the kinase mutants of AsphyA during 10 ns. (**b**) Root mean square fluctuation (RMSF) plots showing the atomic fluctuations for the WT and the kinase mutants of AsphyA. (**c-e**) Superimpositions between the WT and each kinase mutant using C_α atoms of the photosensory core. Superimposed models between WT (blue) and K411L (red)/T418D (green)/D422R (violet) are shown (left panel). ATP molecules binding to the WT proteins are depicted as yellow stick models. Part of the α9 helix (E401 to P430, black box) is shown as a cartoon model (middle panel), and is also shown after rotating the model 90° to show the structural differences clearly. The detailed binding modes of ATP in the mutant systems were simulated in the putative ATP-binding region (right panel). Hydrogen bonding interactions with ATP are represented as dotted green lines, and hydrophobic interacting residues are displayed as line models. (**f-i**) LigPlot diagrams for possible interactions in the putative ATP-binding pocket of the WT (f) and the K411L (g), T418D (h), and D422R mutants (i). The ATP molecule is shown in purple, nitrogen atoms in blue, oxygen atoms in red, and carbon atoms in black. Hydrogen bonds are shown as dashed green lines with bonding lengths.

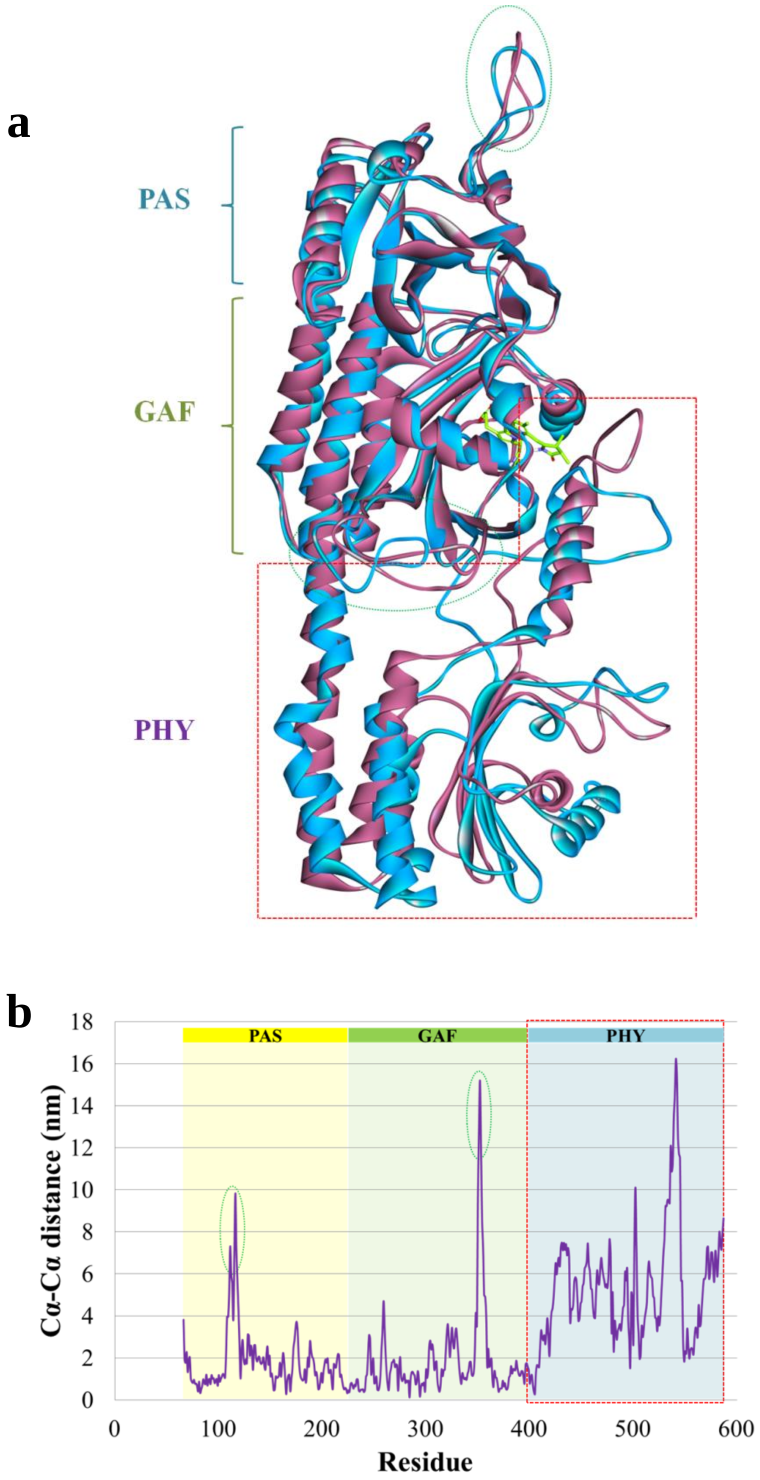

Supplementary Figure 24. Structural comparison between apo- and holo-proteins of the photosensory core of AsphyA. (**a**) Superposition between apo-AsphyA (blue) and holo-AsphyA (magenta) shown as cartoon models. PCB in holo-AsphyA is shown as green sticks. (**b**) C_α-C_α distance calculations between apo- and holo-proteins. There were significant differences in the PHY domain (red box). In the graph, high peaks in the PAS and GAF domains were related to the flexible loop region (green circles in (a) and (b)).

a

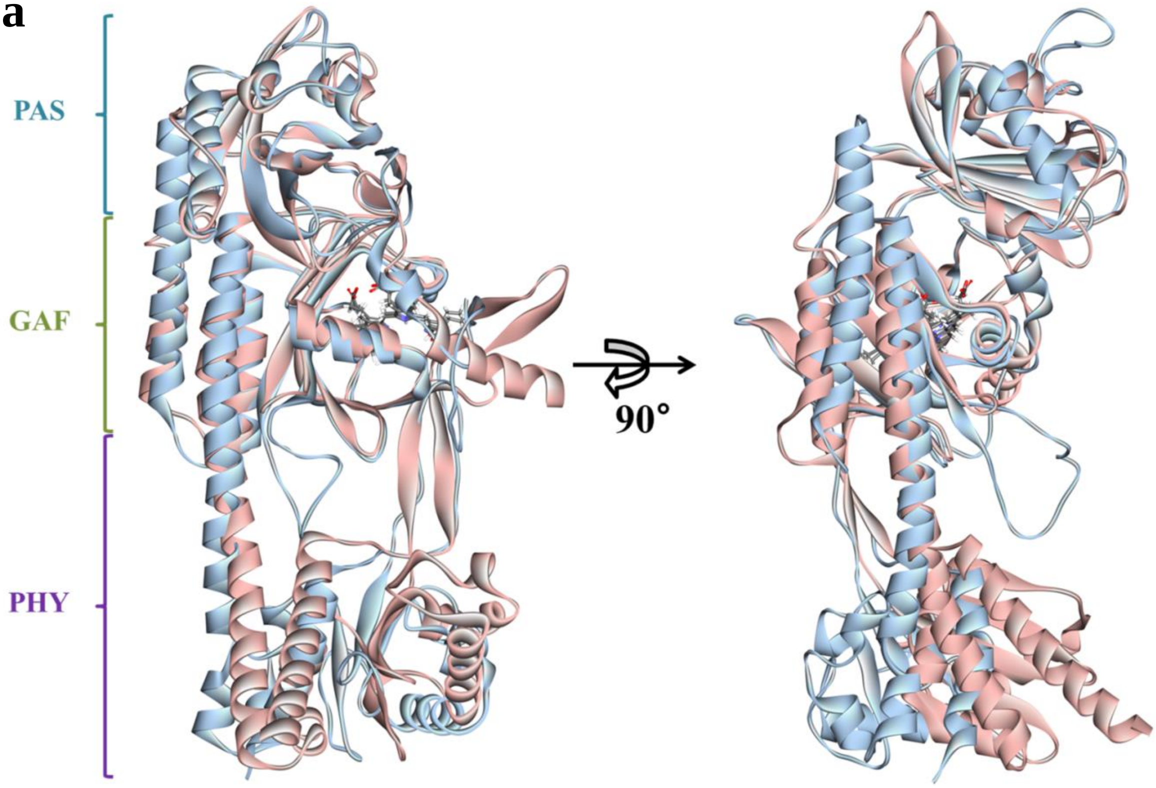

b

```
AsphyA  407  QLREKNILKMQTMLSDMLFREASPLTIVSGNPN-IMDLVKCDGAALLYGGKVWRLRNAPT  465
AtphyB  438  QMSEKRVLRTQTLLCDMLLRDS-PAGIVTQSPS-IMDLVKCDGAAFLYHGKYYPLGVAPS  495
  Cph1  325  FDYRVQLAEHEAVLLDKMTTAADFVEGLTNHPDRLLGLTGSQGAAICFGEKLILVGETPD  384

        466  ESQIHDIAFWLSDVHRDSTGLSTDSLHDAGYPGAAALGDMICGMAVAKINSKDILFWFRS  525
        496  EVQIKDVVEWLLANHADSTGLSTDSLGDAGYPGAAALGDAVCGMAVAYITKRDFLFWFRS  555
        385  EKAVQYLLQWLENREVQDVFFTSS--LSQIYPDAVNFKSVASGLLAIPIARHNFLLWFRP  442

        526  HTAAEIRWGGAKNDPSDMDDSR---RMHPRLSFKAFLEVVKMKSLPWSDYEMDAIHSLQ-  581
        556  HTAKEIKWGGAKHHPEDKDDGQ---RMHPRSSFQAFLEVVKSRSQPWETAEMDAIHSLQ-  611
        443  EVLQTVNWGGDPNHAYEATQEDGKIELHPRQSFDLWKEIVRLQSLPWQSVEIQSALALKK  504

        582  ----LILRGT--  587
        612  ----LILRDS--  617
        505  AIVNLILRQAEE  514
```

Supplementary Figure 25. Structural comparison between the photosensory cores of AtphyB and Cph1. (**a**) Superposition between AtphyB (light blue) and Cph1 (pink) shown as cartoon models. Molecular dynamics simulations were performed to determine the structural differences between AtphyB (PDB code: 4OUR) and Cph1 (PDB code: 2VEA) during the 5 ns simulation time. The closest conformation to average structure during the last 1 ns was selected as the representative structure of each system for structural comparison. The structural comparison showed that the most significant difference was in the PHY domain. (**b**) Multiple sequence alignment with the PHY domains of AsphyA, AtphyB, and Cph1. The sequences were aligned to match the sequences of AsphyA using ClustalW2, which indicated 66.9% identity and 78.5% similarity between AsphyA and AtphyB, and 23.7% identity and 50.5% similarity between AsphyA and Cph1.

a

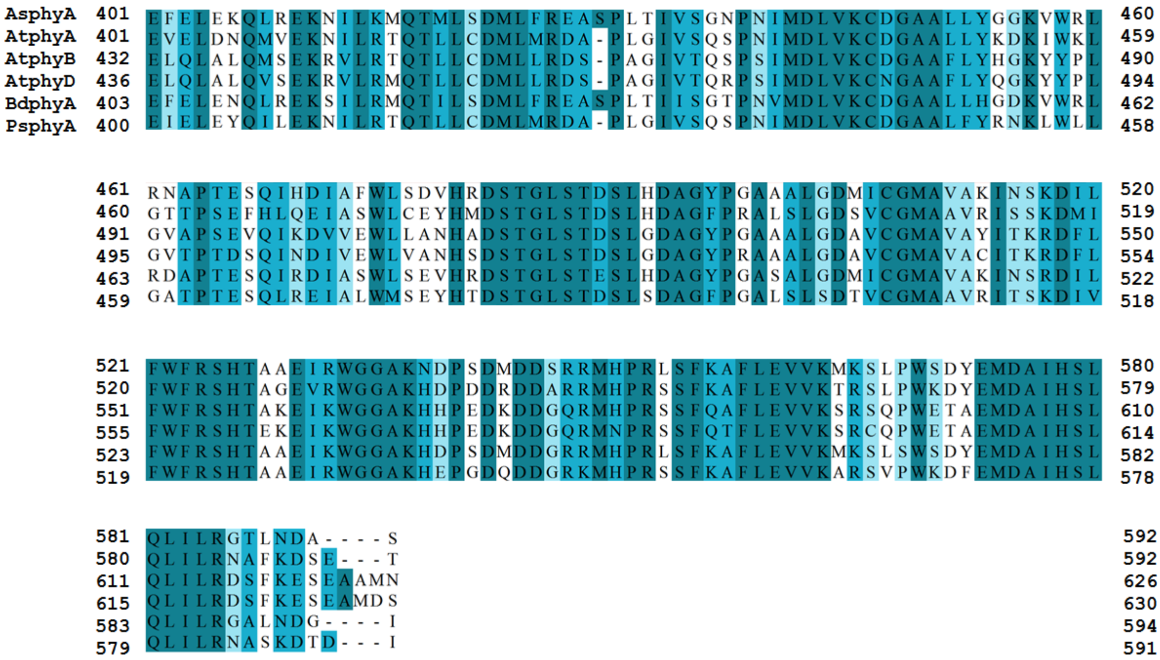

b

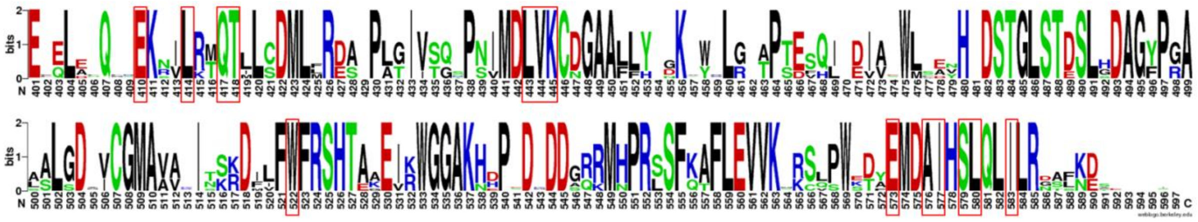

Supplementary Figure 26. Multiple sequence alignment for the PHY domains of phytochromes. (**a**) Multiple sequence alignment for the PHY domains was constructed with AsphyA (E401 to K593), AtphyA (E401 to T592), AtphyB (E432 to N626), AtphyD (E436 to S630), BdphyA (E403 to I594), and PsphyA (E400 to I591). (**b**) Consensus sequences in the PHY domains were determined using WebLOGO with the multiple sequence alignment. In the putative ATP-binding region predicted by 3D structure modeling, conserved amino acid residues that might be important for ATP binding are indicated using red boxes. Amino acids are colored according to their chemical properties: polar amino acids (S, T, Y, C, Q, N) in green, basic amino acids (K, R, H) in blue, acidic amino acids (D, E) in red, and hydrophobic (G, A, V, L, I, P, W, F, M) amino acids in black.

Fig. 1c

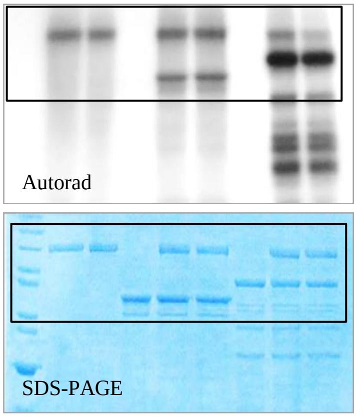

Fig. 1d

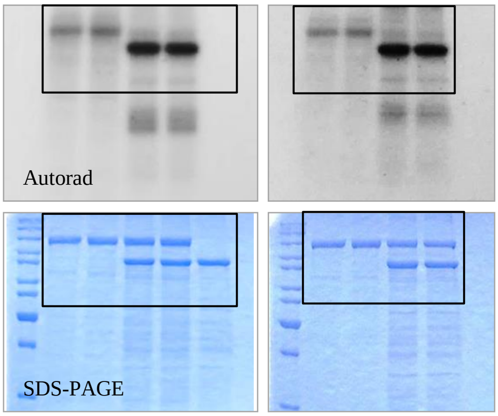

Fig. 2a

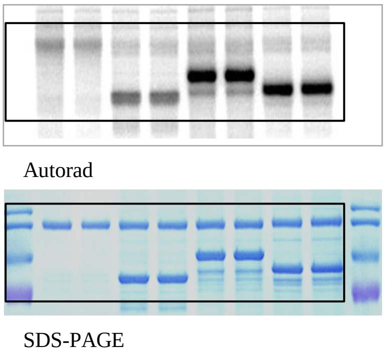

Fig. 2b

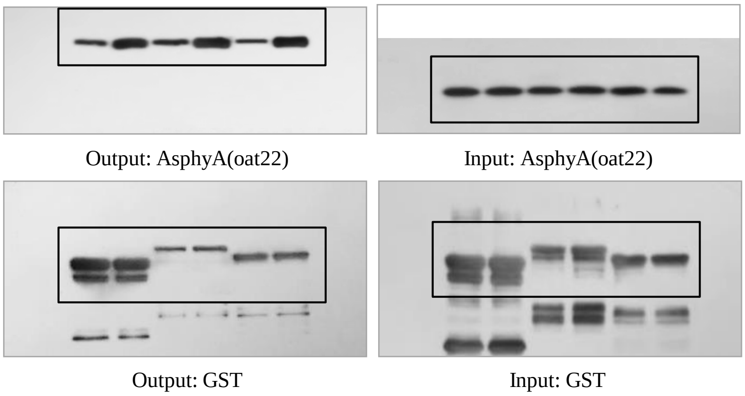

Fig. 2c

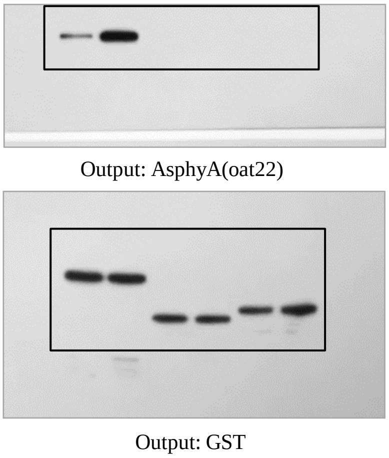

Fig. 2d

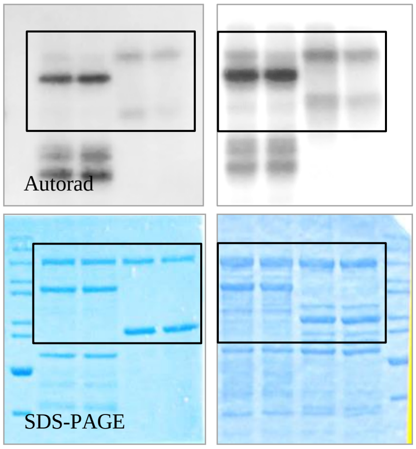

Supplementary Figure 27. Original images of autoradiograms, western blots (WB), and gels.

Fig. 3d

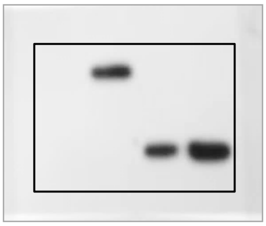

Output: AsphyA(oat22)

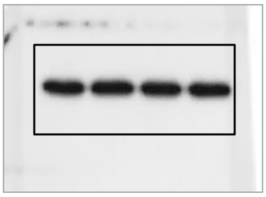

Output: GST

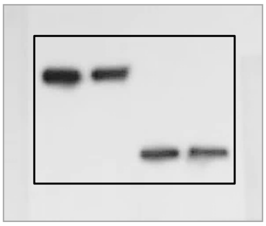

Input: AsphyA(oat22)

Fig. 4a

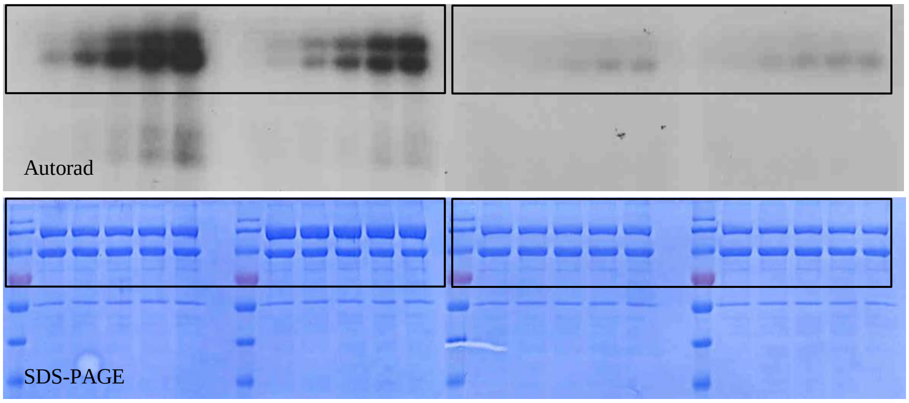

Fig. 3e

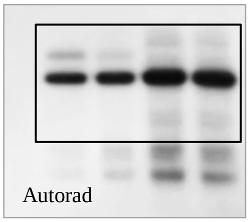

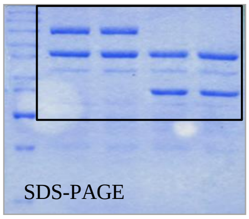

Fig. 3f

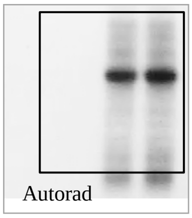

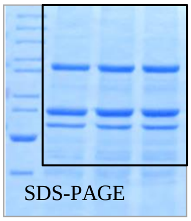

Fig. 4b

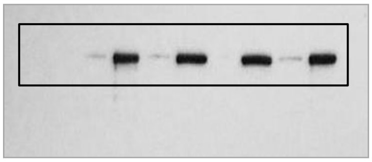

Output: AsphyA(oat22)

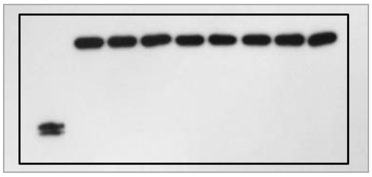

Output: GST

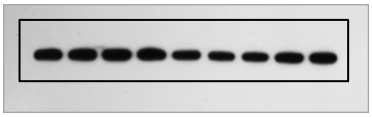

Input: AsphyA(oat22)

Fig. 5a

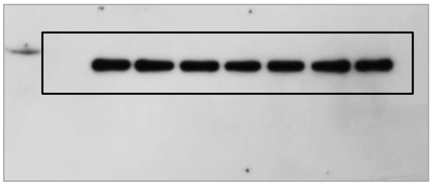

WB: AsphyA(oat22)

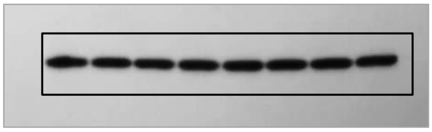

WB: AtTCTP

Supplementary Figure 27. (continued)

Fig. 6a

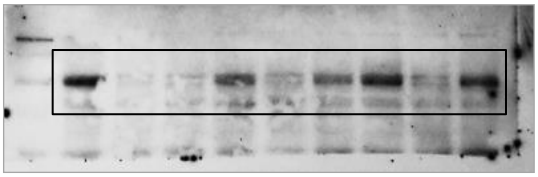

WB: GFP

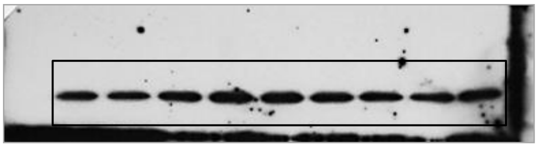

WB: AtTCTP

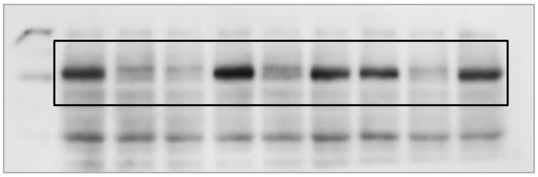

WB: GFP

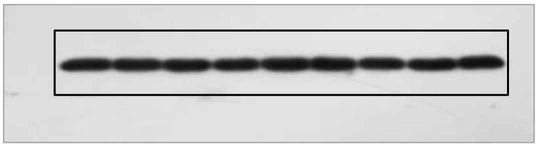

WB: AtTCTP

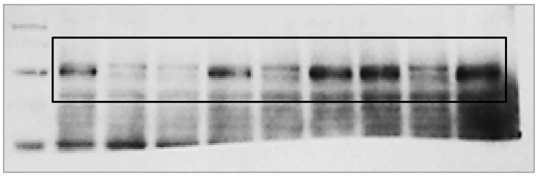

WB: GFP

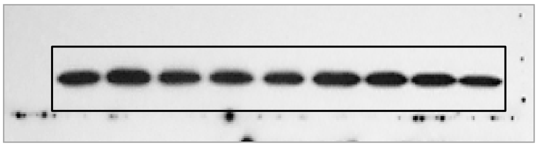

WB: AtTCTP

Fig. 6b

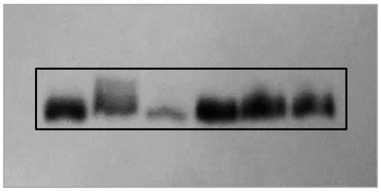

WB: GFP

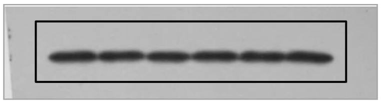

WB: AtTCTP

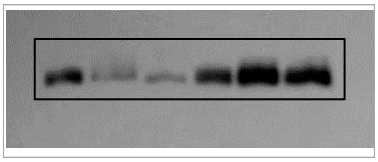

WB: GFP

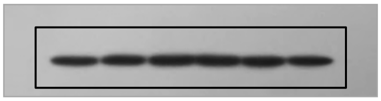

WB: AtTCTP

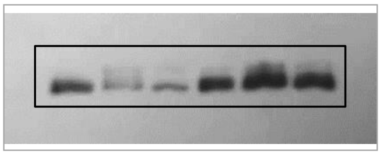

WB: GFP

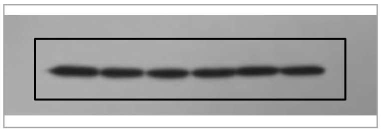

WB: AtTCTP

Supplementary Figure 27. (continued)

Supplementary Fig. 3a

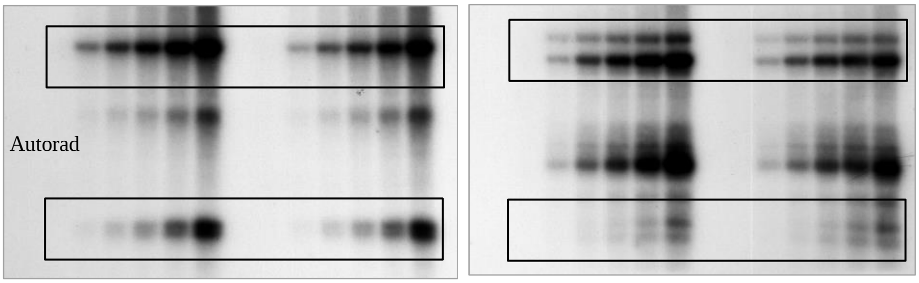

Supplementary Fig. 3c

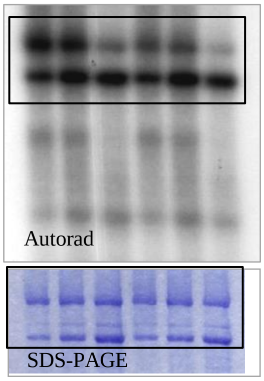

Supplementary Fig. 4a

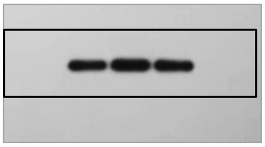

Output: AsphyA(oat22)

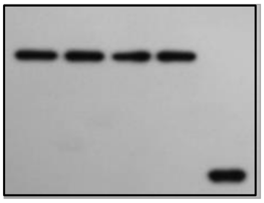

Output: GST

Supplementary Fig. 4b

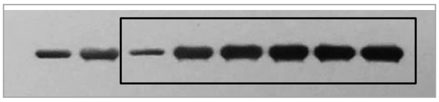

Output: AsphyA(oat22)

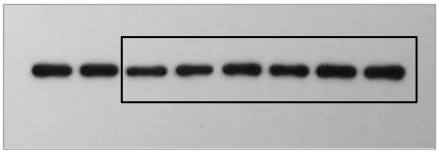

Output: GST

Supplementary Fig. 5

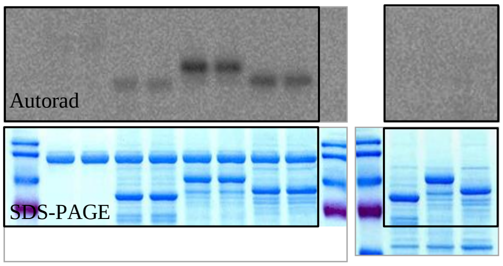

Supplementary Fig. 6a

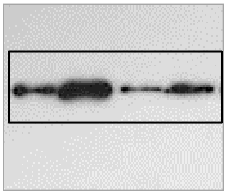

Output: AsphyA(oat22)

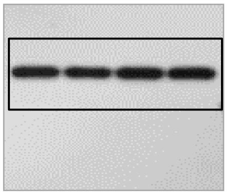

Output: GST

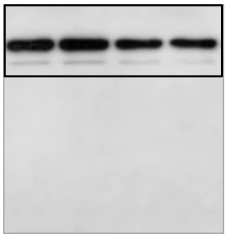

Output: GST

Supplementary Fig. 6b

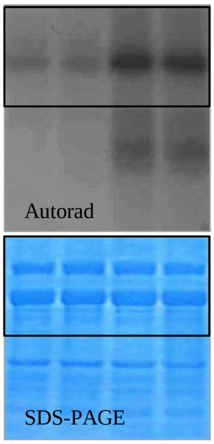

Supplementary Fig. 9

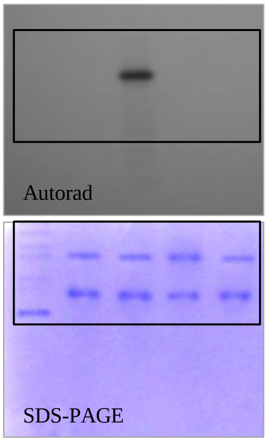

Supplementary Figure 27. (continued)

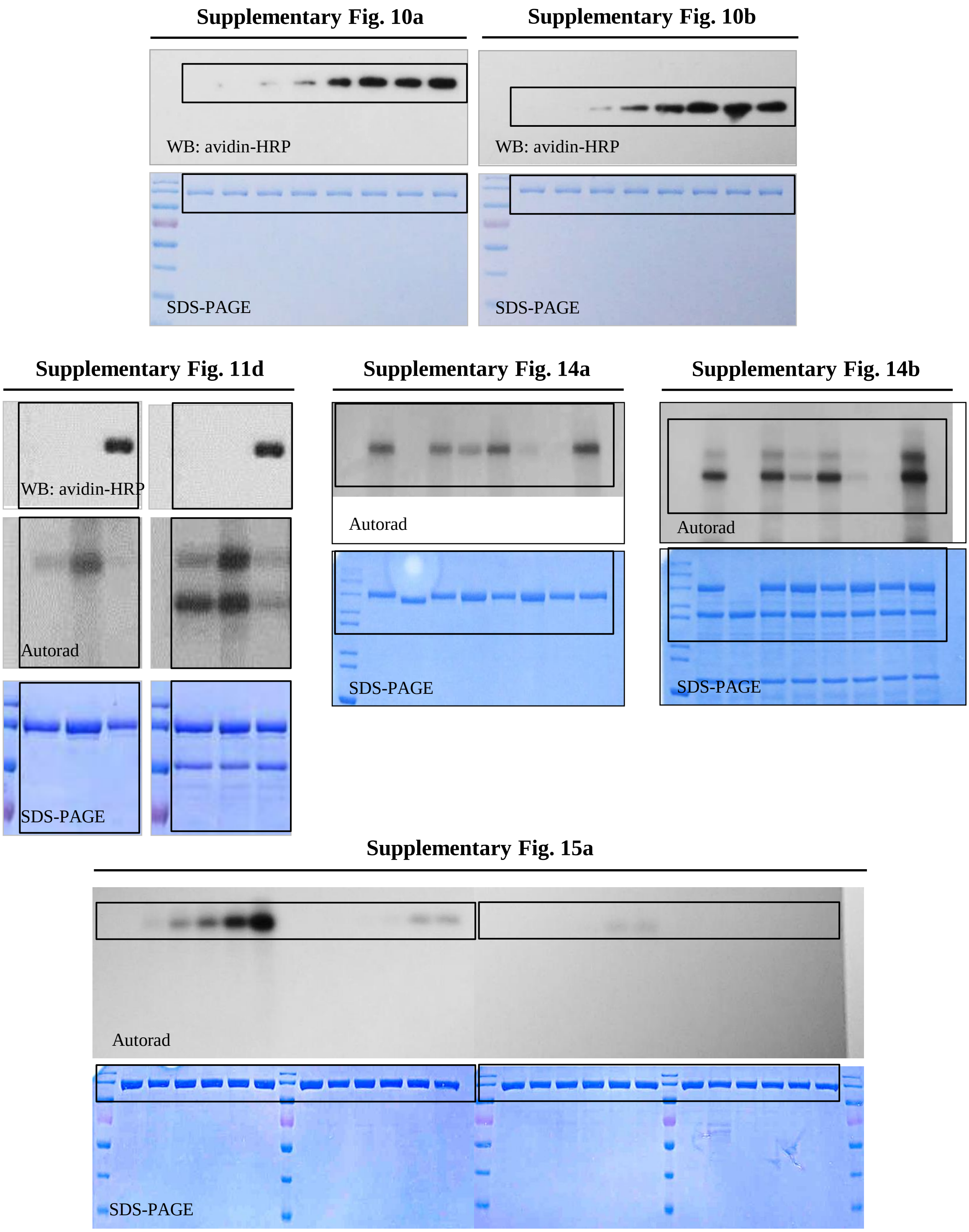

Supplementary Figure 27. (continued)

Supplementary Fig. 15b

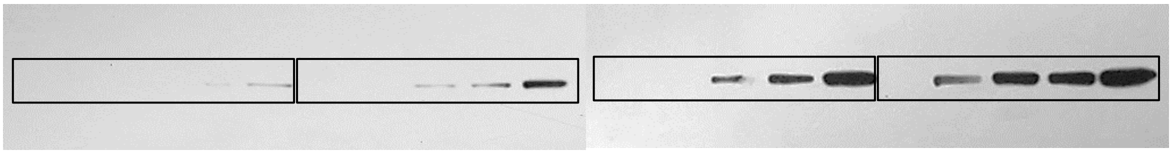

WB: avidin-HRP

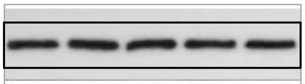

WB: AsphyA(oat22)

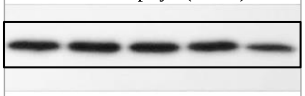

WB: AsphyA(oat22)

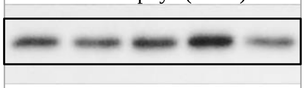

WB: AsphyA(oat22)

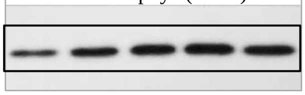

WB: AsphyA(oat22)

Supplementary Fig. 15d

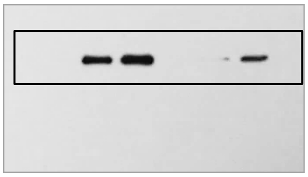

WB: avidin-HRP

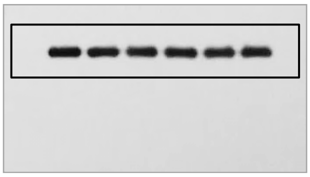

WB: AsphyA(oat22)

Supplementary Fig. 18b

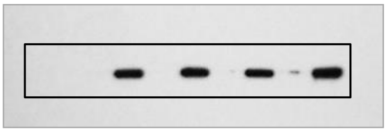

Output: AsphyA(oat22)

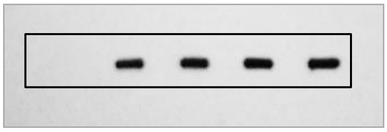

Output: AsphyA(oat22)

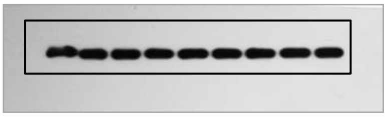

Input: AsphyA(oat22)

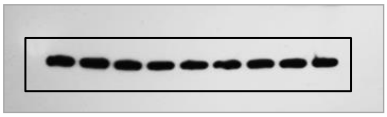

Input: AsphyA(oat22)

Supplementary Figure 27. (continued)

Supplementary Fig. 18c

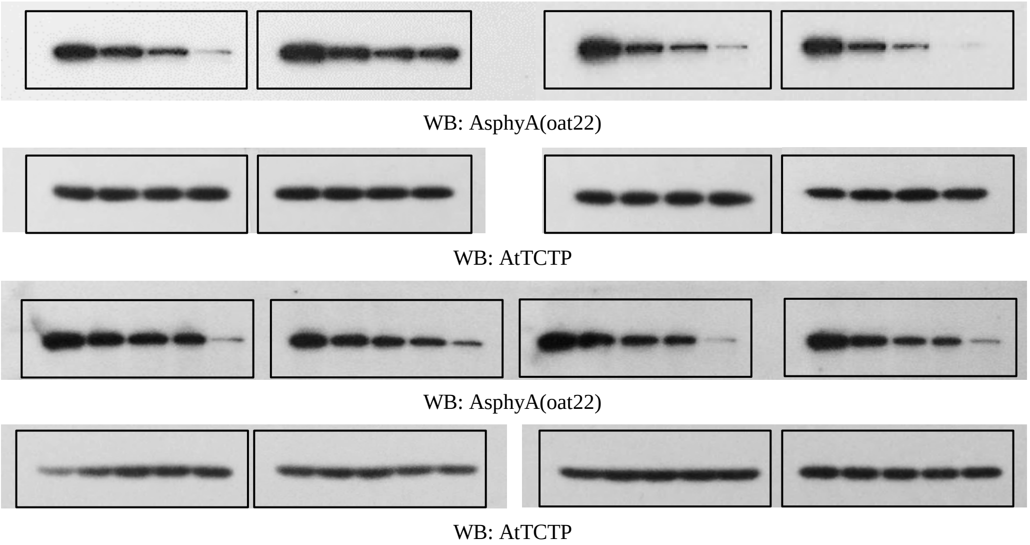

Supplementary Fig. 19a

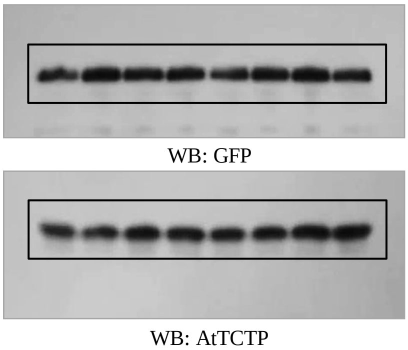

Supplementary Fig. 19b

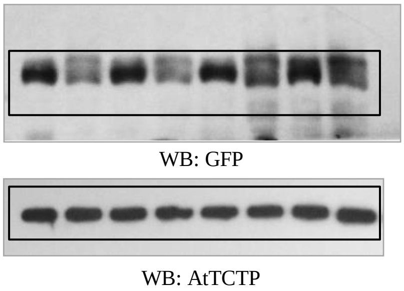

Supplementary Fig. 20a

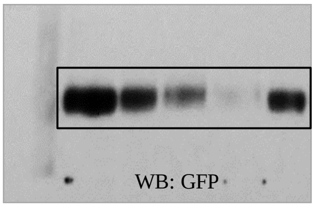

Supplementary Fig. 20b

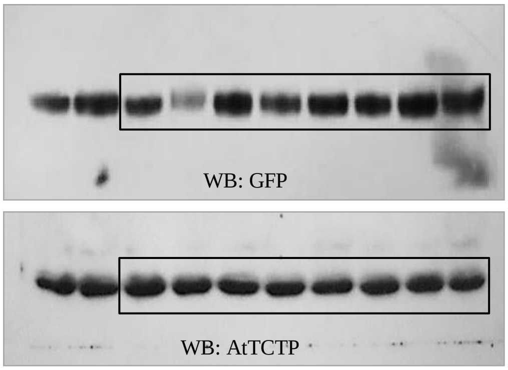

Supplementary Figure 27. (continued)

Supplementary Table 1. Molecular interaction between ATP and AsphyA in four model systems predicted by molecular dynamics (MD) simulations.

System	Hydrogen bonds	Hydrophobic interactions	π-interaction
WT	R409, Q417, S421, S570, A576	E410, I413, L414, T418, N439, L443, V444, K445, W522,Y572, E573, I577, S579, L580, I583	L580 (π-sigma)
K411L	K406, R409, Q417, L443	E245, E410, I413, L414, D442, K445, Y572, E573, A576	-
T418D	K406, R409, K445, E573	E245, E410, L414, V444, W522, R524, A576	-
D422R	R409, K445	E410, K411, I413, L414, Q417, Y572, E573, D575	L414 (π-sigma)

Supplementary Table 2. Primers used for the present study.

Oligo Name	DNA Sequence
For full-length phytochromes	
AsphyA	5′-CGGGATCCACCATGGCTTCCTCAAGGCCTGCTTCC-3′ (Forward)<br>5′-TCGCGTCGACTTGTCCCATTGCTGTTGGAGC-3′ (Reverse)
BdphyA	5′-GCGGATCCACCATGGCTTCCAGGCCTACTCAGTCTTCCAG-3′ (Forward)<br>5′-GCTAGCGCTTTGTCCAACTGCTGTTGGAGCCGAAGCAAGTTC-3′ (Reverse)
PsphyA	5′-CGGGATCCACCATGGCAACCACGAGGCCTAGCC-3′ (Forward)<br>5′-TCGCGTCGACCTTTCAACTTATGAGCTGC-3′ (Reverse)
AtphyA	5′-CTCTGGATCCACCATGGCAGGCTCTAGGCCGACTCAG-3′ (Forward)<br>5′-TCGCGTCGACCTTGTTTGCTGCACGGAGTTC-3′ (Reverse)
AtphyB	5′-CTCCCCGGGTACGTAACCATGGTTTCCGGAGTCGGGG-3′ (Forward)<br>5′-TCGCAGCGCTATATGGCATCATCAGCATCATG-3′ (Reverse)
AtphyD	5′-CTCGGTACCTACGTACCATGGTCTCCGGAGGTGGTAG-3′ (Forward)<br>5′-CTCCCCGGGTGAAGAGGGCATCATCATCATTAG-3′ (Reverse)
For the domain-deleted mutants of AsphyA	
A875	5′-TCGCGTCGACAGCAACATGAATAAAACAGAATAC-3′ (Reverse)
A610	5′-TCGCGTCGACATCAAGTTTTAGATCACCAATCTG-3′ (Reverse)
A407	5′-TCGCGTCGACCTGTTTCTCTAATTCAAACTCCCT-3′ (Reverse)
Δ65	5′-CGGGATCCACCATGGTCATAGCCTACTTACAGCAC-3′ (Forward)
AC	5′-CGGGATCCATGTATGAAATGGATGCTATTCATTCA-3′ (Forward)
For the eGFP-fused constructs	
AsphyA	5′-CGGAATTCATGGCTTCCTCAAGGCCTGCTTCC-3′ (Forward)<br>5′-TCGCGGATCCTTGTCCCATTGCTGTTGGAGC-3′ (Reverse)
PIF3	5′-GGGGTACCATGCCTCTGTTTGAGCTTTTC-3′ (Forward)<br>5′-CGGGATCCCGACGATCCACAAAACTGATC-3′ (Reverse)
For the APA mutants of PIF3	
F203A	5′-GCAGCGCTTGCACCTAACTCATCAATCCCCT-3′ (Reverse)<br>5′-GTCAACTTTTCACATGCCTTACGCCCTGCAAC-3′ (Forward)
F209A	5′-CATTTCTTACGCCCTGCAACTGCTGCGAAGACTACTAATAATAAC-3′ (Forward)<br>5′-GTTATTATTAGTAGTCTTCGCAGCAGTTGCAGGGCGTAAGAAATG-3′ (Reverse)

Supplementary Table 2. (continued)

Oligo Name	DNA Sequence
For the site-mutants of AsphyA	
E410Q	5′-GAGAAACAGTTGCGTCAGAAGAACATACTGAAG-3′ (Forward)<br>5′-CTTCAGTATGTTCTTCTGACGCAACTGTTTCTC-3′ (Reverse)
K411L	5′-GAGAAACAGTTGCGTGAGCTCAACATACTGAAGGATGCAA-3′ (Forward)<br>5′-TTGCATCTTCAGTATGTTGAGCTCACGCAACTGTTTCTC-3′ (Reverse)
L414Q	5′-CGTGAGAAGAACATACAGAAGATGCAAACAATG-3′ (Forward)<br>5′-CATTGTTTGCATCTTCTGTATGTTCTTCTCACG-3′ (Reverse)
T418D	5′-AACATACTGAAGATGCAAGATATGCTCTCTGATATGTTG-3′ (Forward)<br>5′-CAACATATCAGAGAGCATATCTTGCATCTTCAGTATGTT-3′ (Reverse)
D422R	5′-CAAACAATGCTCTCTAGAATGTTGTTCCGAGAAGCC-3′ (Forward)<br>5′-GGCTTCTCGGAACAACATTCTAGAGAGCATTGTTTG-3′ (Reverse)
L424E	5′-ATGCTCTCTGATATGGAATTCCGAGAAGCCTCTCCC-3′ (Forward)<br>5′-GGGAGAGGCTTCTCGGAATTCCATATCAGAGAGCAT-3′ (Reverse)
For PIFs, PKS1, FHY1, and FHL	
PIF1	5′-CGAATTCATGGATCCTCAGCAGCAACCTTC-3′ (Forward)<br>5′-GCCCGGGACCTGTTGTGTGGTTTCCGTG-3′ (Reverse)
PIF3	5′-CGGATCCATGCCTCTGTTTGAGCTTTTCAG-3′ (Forward)<br>5′-GCCCGGGCGACGATCCACAAAACTGATC-3′ (Reverse)
∆210-PIF3	5′-CGGGATCCATGAAGACTACTAATAATAACCTT-3′ (Forward)<br>5′-GCCCGGGCGACGATCCACAAAACTGATC-3′ (Reverse)
PIF4	5′-CGAATTCATGGAACACCAAGGTTGGAGTTTTGAG-3′ (Forward)<br>5′-GCCCGGGGTGGTCCAAACGAGAACCGTCG-3′ (Reverse)
PIF7	5′-CGGGATCCATGTCGAATTATGGAGTTAAAGAG-3′ (Forward)<br>5′-GGAGCGCTATCTCTTTTCTCATGATTCGAAGAAC-3′ (Reverse)
PKS1	5′-CGGGATCCATGGTGACACTAACACCATCTTC-3′ (Forward)<br>5′-GGAGCGCTCTGACTATAAAGAAGAGATGATTG-3′ (Reverse)
FHY1	5′- CGGGATCCATGCCTGAAGTGGAAGTGGAT-3′ (Forward)<br>5′-GGAGCGCTCAGCATTAGCGTTGAGAAGTA-3′ (Reverse)
FHL	5′- CGGGATCCATGGATGATGCAGATAAG-3′ (Forward)<br>5′-GGAGCGCTCATCATGAGTGTAGAAAAGTA-3′ (Reverse)

Supplementary Note 1. Preparation of recombinant phytochrome proteins.

There are five isoforms of plant phytochromes (phyA-phyE), and these can be classified as light-labile type I (phyA) and light-stable type II (phyB-E) phytochromes. In this study, we initially tried to express five isoforms of *Arabidopsis thaliana* phytochromes (AtphyA to AtphyE) using the *Pichia pastoris* protein expression system. Type II AtphyB and AtphyD recombinant proteins were expressed well and purified sufficiently, while AtphyC and AtphyE proteins were not expressed in this system. In the case of AtphyA, the expression level was very low, but we could get purified proteins for phosphorylation analysis. In addition, we obtained recombinant type I phytochromes from other plant species, including *Brachypodium distachyon* phyA (BdphyA) and *Pisum sativum* phyA (PsphyA) as well as *A. sativa* phyA (AsphyA). We then performed phosphorylation assays of these purified recombinant phytochrome proteins in the absence or presence of histone H1.

Supplementary Note 2. Domain structure of phytochrome.

The phytochrome molecule is composed of two major regions, the globular N-terminal chromophore-binding region (~65 kDa) and the structurally extended C-terminal region (~55 kDa). The N-terminal region consists of an N-terminal extension (NTE) and the photosensory core (i.e., PAS-GAF-PHY tri-domain). The NTE is dispensable for chromophore binding but necessary for the biological activity[1]. The PAS (PER/ARNT/SIM) and GAF (cGMP phosphodiesterase/Adenylate cyclase/FhlA) domains in the region are known to be necessary for the bilin lyase activity to attach chromophore, and the PHY domain is known as a phytochrome-specific GAF-related domain[2,3]. The C-terminal region contains a repeat of PAS-related domains (PRD) and the histidine kinase-related domain (HKRD). The PRD is known to have regulatory roles, such as phytochrome dimerization and nuclear localization[4,5].

Supplementary Note 3. Prediction of putative ATP-binding region in the photosensory core of AsphyA.

To identify the ATP-binding sites in AsphyA, we initially tried to determine the 8-azido-ATP-labeled peptide by proteomics using LC-MS/MS analyses of trypsin-digested fragments with the photosensory core, but failed to identify the labeled peptide precisely. However, we found that two tryptic peptides, "^{318}APHS*C*HLQYMENMNSIASLVMAVVVNENEEDDEAESEQPAQQQ^{360}" (containing chromophore-binding cysteine residue in italics) and "^{416}MQTMLSDMLFR^{426}", were not detected from the analysis of trypsin-digested fragments with the 8-azido-ATP-labeled photosensory core. On the other hand, the 416-426 peptide was detected from the analysis of the photosensory core without 8-azido-ATP labeling, while the 318-360 peptide was not detected. Since the 318-360 peptide has a chromophore, we speculated that the 416-426 peptide might be labeled with azido-ATP. Coincidentally, a previous report suggested the peptide sequence "^{403}ELEKQLREKNILK^{415}" as a putative nucleoside triphosphate binding site in phyA[6]. Since the 403-415 peptide contained several positive amino acid residues (KxxRxKxxxK) that can interact with the phosphate group of ATP, it is speculated that the 416-426aa peptide might be labeled with the photoactive azido (-N_3) group of the adenine ring in 8-azido-ATP. Thus, we performed site-directed mutagenesis of the conserved amino acid residues in those peptides (i.e., 403-415 and 416-426) to obtain AsphyA mutants with reduced kinase activity.

Supplementary Note 4. Predicted ATP binding site in the photosensory core.

In the present study, we identified the PAS-GAF-PHY photosensory core in the N-terminal region of AsphyA as the protein kinase domain for PIF3 phosphorylation. This is somewhat surprising because the photosensory core shows no homology to the sequence of a known kinase domain. Accordingly, we expanded our study to include computational approaches to predict the ATP-binding site in the photosensory core. To accomplish this, we produced a three-dimensional structure by homology modeling using the crystal structures of AtphyB (PDB code: 4OUR) and PaBphP (PDB code: 3C2W) as templates, and a putative ATP-binding region in the photosensory core was predicted by molecular docking and molecular dynamics (MD) simulations. The modeled structure with PCB showed that an ATP molecule was bound in the PHY domain of the photosensory core (Supplementary Fig. 21a), and the mutation positions (K411, T418 and D422) used to generate AsphyA kinase mutants were located in the interface between the α9 helices, which are involved in the formation of the putative ATP-binding pocket (Supplementary Fig. 21b). The ATP-interacting amino acid residues in the pocket were involved in hydrogen bond interaction, hydrophobic interaction, and π-interaction with the ATP molecule (Supplementary Fig. 21c, Supplementary Table 1). We also produced a homology-modeled structure of AtphyB, which predicted that the ATP molecule would bind in the PHY domain (Supplementary Fig. 22). Therefore, our modeling data suggested a putative ATP-binding site in the PHY domain of phytochromes.

Our results also showed that the kinase mutants of AsphyA had significantly reduced affinity to ATP with higher K_d values (Fig. 4c, Supplementary Fig. 12). Accordingly, we further compared differences in the structure and ATP-binding mode between wild-type and kinase mutants by MD simulations. The simulations for the four systems were performed stable during 10 ns simulation time, showing root-mean-square deviation (RMSD) values of 0.4 ~ 0.5 nm (Supplementary Fig. 23a). In addition, root mean square fluctuation (RMSF) plots showed similar profiles for the four systems (Supplementary Fig. 23b), indicating that the models could be used for further analysis. The results of superposition between WT and each kinase mutant showed that the RMSD values were 0.48 nm, 0.41 nm, and 0.43 nm for K411L, T418D, and D422R, respectively (Supplementary Fig. 23c-e, left panels). These relatively high RMSD values imply that there are structural differences between the WT and the kinase mutants, in which conformations of the PHY domain including the α9 helices have been significantly altered (Supplementary Fig. 23c-e, middle panels). The molecular docking results also indicated that the ATP molecule in WT is bound to the ATP-binding pocket much deeper than that in the kinase mutants. Compared with the WT, the positions of the ATP molecule in the binding pocket of the kinase mutants were quite different, among which the T418D and D422R models showed completely different ATP-binding modes with the changes of ATP-interacting residues in the binding pocket (Supplementary Fig. 23f-i, Supplementary Table 1). Therefore, our modeling results suggest that the reduced ATP-binding of the kinase mutants used in this study is caused by the changes in the ATP-binding modes, which might increase K_d values compared with wild-type AsphyA.

Our results also showed that the protein structure assembled with chromophore is required for the observed phytochrome kinase activity on PIF3 (Fig. 3f, Supplementary Fig 9). Thus, we compared the homology-modeled structures with or without PCB, and found that C_α-C_α distances for the MD snapshot structures between the apo- and holo-proteins were significantly different in the PHY domain (Supplementary Fig. 24), suggesting that chromophore binding induces conformational changes in the PHY domain. Moreover, the structural comparison between AtphyB (PDB code: 4OUR) and Cph1 (PDB code: 2VEA) indicated that the orientations of PAS-GAF domains are similar, but that of the PHY domain

is quite different (Supplementary Fig. 25a). A multiple sequence alignment with the PHY domains of AsphyA, AtphyB, and Cph1 also revealed 66.9% identity and 78.5% similarity between the plant phytochromes (i.e., AsphyA and AtphyB), but lower identity (23.7%) and similarity (50.5%) between AsphyA and Cph1 (Supplementary Fig. 25b). Thus, the kinase activity of the AsphyA photosensory core may be attributed to the structural and sequence differences between the PHY domains of plant and bacterial phytochromes.

Our modeling studies also allowed us to predict which amino acids were important for ATP binding and kinase activity (Supplementary Fig. 21, Supplementary Table 1). It is speculated that the hydrogen bond interaction between Gln417 and the adenine ring of ATP might play an important role in ensuring stable ATP-binding, because this hydrogen bonding disappeared in the T418D and D422R mutants (Supplementary Fig. 23). In addition, we also found that Arg409, Glu410, Lys445, Ser570 and Glu573 interacted with the phosphate group of ATP, which might contribute to maintaining a correct ATP-binding mode in the PHY domain. Especially, when we compared the ATP-binding patterns of AsphyA with known serine/threonine protein kinases such as human p21-activated kinase (PDB ID: 3Q53) and CDK9 (PDB ID: 3BLQ), it is speculated that Lys445 might play an important role in the interaction of the PHY domain with the α-phosphate group of ATP and that Glu573 may potentially act as a catalytic base. Finally, from multiple sequence alignments of the PHY domains of six phytochromes used in this study (see Fig. 1a,b), we determined the consensus sequences in the PHY domains using WebLOGO (Supplementary Fig. 26). Among the consensus sequences, amino acid residues, including Glu410, Leu414, Gln417, Thr418, Leu443, Val444, Lys445, Trp522, Glu573, Ala576, Ile577, Ser579, Leu580, and Ile583, are predicted to be involved in the ATP binding. Therefore, these residues might play important roles in both ATP binding and kinase activity in the AsphyA photosensory core.

Supplementary References

1. Cherry, J. R., Hondred, D., Walker, J. M. & Vierstra, R.D. Phytochrome requires the 6-kDa N-terminal domain for full biological activity. *Proc. Natl. Acad. Sci. USA* **89**, 5039-5043 (1992).

2. Wagner, J. R., Brunzelle, J. S., Forest, K. T. & Vierstra, R.D. A light-sensing knot revealed by the structure of the chromophore-binding domain of phytochrome. *Nature* **438**, 325-331 (2005).

3. Essen, L. O., Mailliet, J. & Hughes, J. The structure of a complete phytochrome sensory module in the Pr ground state. *Proc. Natl. Acad. Sci. USA* **105**, 14709-14714, 2008.

4. Quail, P. H., Boylan, M. T., Parks, B. M., Short, T. W., Xu, Y. & Wagner, D. Phytochromes: photosensory perception and signal transduction. *Science* **268**, 675-680 (1995).

5. Chen, M., Tao, Y., Lim, J., Shaw, A. & Chory, J. Regulation of phytochrome B nuclear localization through light-dependent unmasking of nuclear-localization signals. *Curr. Biol.* **15**, 637-642 (2005).

6. Wong, Y. S. & Lagarias, J. C. Affinity labeling of Avena phytochrome with ATP analogs. *Proc. Natl. Acad. Sci. USA* **86,** 3469-3473 (1989).
